# Synthesis and Pharmacological Evaluation of Fluorinated Quinoxaline‐Based κ‐Opioid Receptor (KOR) Agonists Designed for PET Studies

**DOI:** 10.1002/cmdc.202000502

**Published:** 2020-09-01

**Authors:** Giovanni Tangherlini, Frederik Börgel, Dirk Schepmann, Samuel Slocum, Tao Che, Stefan Wagner, Katrin Schwegmann, Sven Hermann, Nadine Mykicki, Karin Loser, Bernhard Wünsch

**Affiliations:** ^1^ Institut für Pharmazeutische und Medizinische Chemie Universität Münster Corrensstraße 48 48149 Münster Germany; ^2^ Cells-in-Motion Cluster of Excellence (EXC 1003-CiM) Westfälische Wilhelms-Universität Münster 48149 Münster Germany; ^3^ Department of Pharmacology University of North Carolina at Chapel Hill School of Medicine Chapel Hill NC 27599 USA; ^4^ Department of Anesthesiology Washington University School of Medicine 660 S. Euclid Ave. St. Louis MO 63110 USA; ^5^ Department of Nuclear Medicine University Hospital Münster Albert-Schweitzer-Campus 1, Building A1 48149 Münster Germany; ^6^ European Institute for Molecular Imaging (EIMI) University of Münster Waldeyerstraße 15 48149 Münster Germany; ^7^ Department of Dermatology University of Münster von-Esmarch-Str. 58 48149 Münster Germany; ^8^ CRC1009 Breaking Barriers and CRC-TR 128 Multiple Sclerosis University of Münster von-Esmarch-Str. 58 48149 Münster Germany

**Keywords:** anti-inflammatory activity, effector cells, fluorine, opioid receptor agonists, perhydroquinoxaline, PET tracers

## Abstract

κ‐Opioid receptors (KORs) play a predominant role in pain alleviation, itching skin diseases, depression and neurodegenerative disorders such as multiple sclerosis. Therefore, imaging of KOR by a fluorinated PET tracer was envisaged. Two strategies were followed to introduce a F atom into the very potent class of cis,trans‐configured perhydroquinoxalines. Whereas the synthesis of fluoroethyltriazole **2** has already been reported, fluoropyrrolidines **14** (1‐[2‐(3,4‐dichlorophenyl)acetyl]‐8‐[(R)‐3‐fluoropyrrolidin‐1‐yl]‐perhydroquinoxalines) were prepared by S_N_2 substitution of a cyclic sulfuric acid derivative with hydroxypyrrolidine and subsequent transformation of the OH moiety into a F substituent. Fluoropyrrolidines **14** showed similar low‐nanomolar KOR affinity and selectivity to the corresponding pyrrolidines, but the corresponding alcohols were slightly less active. In the cAMP and β‐arrestin assay, **14b** (proton at the 4‐position) exhibited similar KOR agonistic activity as U‐50,488. The fluoro derivatives **14b** and **14c** (CO_2_CH_3_ at the 4‐position) revealed KOR‐mediated anti‐inflammatory activity as CD11c and the IFN‐γ production were reduced significantly in mouse and human dendritic cells. Compounds **14b** and **14‐c** also displayed anti‐inflammatory and immunomodulatory activity in mouse and human T cells. The PET tracer [^18^F]‐**2** was prepared by 1,3‐dipolar cycloaddition. In vivo, [^18^F]‐**2** did not label KOR due to very fast elimination kinetics. Nucleophilic substitution of a mesylate precursor provided [^18^F]‐**14c**. Unfortunately, defluorination of [^18^F]‐**14c** occurred in vivo, which was analyzed in detail by *in vitro* studies.

## Graphical abstract

κ‐Opioid receptors (KORs) play a prominent role in pain alleviation, skin diseases, depression and neurodegenerative disorders. Two strategies were pursued to prepare fluorinated tracers for positron emission tomography based on the perhydroquinoxaline class of KOR agonists. The agonists showed high KOR affinity, selectivity, agonistic activity and KOR‐mediated anti‐inflammatory and immunomodulatory activity. One tracer was quickly eliminated, another was defluorinated.

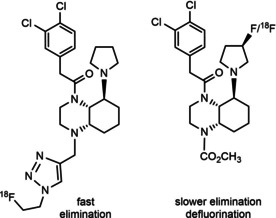

## 1 Introduction

The analgesic activity of opium had already been reported by Theophrastus in the third century BC, but it took more than 2000 years until the alkaloids of opium responsible for the pharmacological effects as well as the opioid system consisting of opioid receptors and their endogenous ligands were identified. Today, three opioid receptors that alleviate pain upon activation are known: the μ‐opioid receptor (MOR), the δ‐opioid receptor (DOR) and the κ‐opioid receptor (KOR). According to sequence homology, the nociceptive opioid receptor (NOR) also belongs to the class of opioid receptors, but it behaves differently as it induces pain upon activation.^1^, ^2^, ^3^

The clinically used strong analgesics activate predominantly MOR, which is associated with the development of physical and psychical dependence, obstipation and dangerous respiratory depression. In this project, we focus on KOR, as activation of KOR also leads to strong analgesia, but to a modified side‐effect profile without respiratory depression and reduced addiction potential. However, sedation, strong diuresis and dysphoria have been observed after application of KOR agonists.^4^, ^5^

**Figure 1:**
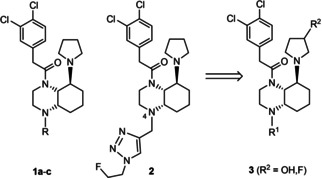
Potent KOR agonists **1b** (R=H) and **2** ameliorating MS‐like symptoms in MOG induced EAE. The effect of higher polarity of compounds **3** (R^2^=OH) with an additional OH moiety at the pyrrolidine ring will be studied. Compounds **2** and **3** (R^2^=F) will be developed into fluorinated PET tracers for imaging of KOR.

Whereas centrally and peripherally acting KOR agonists can be used for the treatment of severe pain, visceral pain and itching skin diseases, KOR antagonists have antidepressant, anxiolytic, antiaddictive, anticonvulsant and anti‐seizure potential.^5^, ^6^, ^7^, ^8^, ^9^, ^10^ Recent studies showed the central role of KOR in the pathogenesis of multiple sclerosis (MS), an inflammatory neurodegenerative disease.^11^, ^12^, ^13^ A decreased level of KOR mRNA was found in Theiler's encephalomyelitis mouse model mimicking parts of the MS pathology.^11^ In the myelin oligodendrocyte glycoprotein (MOG) induced experimental autoimmune encephalomyelitis (EAE) mouse model of MS activation of KOR was able to prevent neuronal damage and promote remyelination.^12^ Very recently, we showed that KOR agonists **1b** (R=H) and **2** exhibit potent anti‐inflammatory and immunomodulatory activity in primary mouse and human immune cells. Moreover, both KOR agonists led to almost complete protection of mice from developing MS‐like symptoms in the MOG induced EAE^13^ (Figure 1).

**Figure 2:**
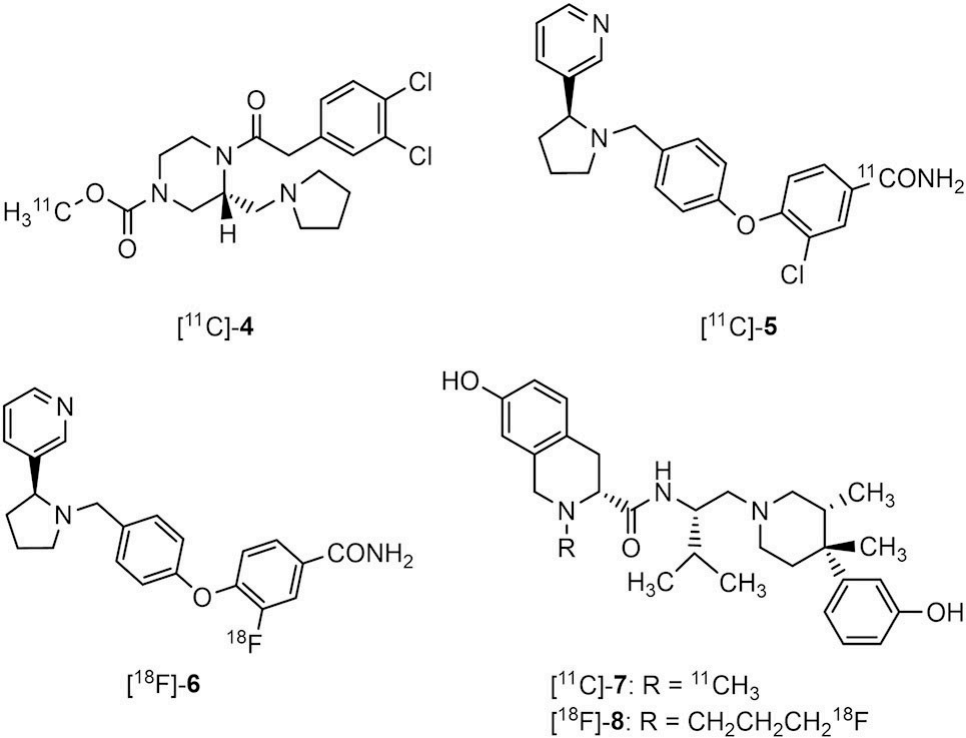
PET tracers for imaging of KORs. Whereas **4** represents a KOR agonist, compounds **5**–**8** are KOR antagonists. [^11^C]‐**4**, [^11^C]‐**5** and [^11^C]‐**7** are labeled with short‐lived carbon‐11 and [^18^F]‐**6** and [^18^F]‐**8**, are labeled with fluorine‐18 with a half‐life of 110 min.

Due to their prominent role in pain alleviation, itching skin diseases, depression, anxiety, epilepsy and neurodegenerative disorders such as MS, the localization, expression level, activity and density of KOR is of high interest. Positron emission tomography (PET) represents a non‐invasive modality, which allows imaging of biological targets *in vivo* upon using appropriate radioligands. In 2005, the first PET tracer addressing KOR, the carbon‐11‐labeled KOR agonist [^11^C]‐**4** ([^11^C]GR103545) was reported (Figure 2). Although **4** showed high KOR affinity, activity and selectivity and [^11^C]‐**4** led to promising results in various animal models (mice, primates), human studies were not satisfying.^14^, ^15^, ^16^, ^17^, ^18^, ^19^, ^20^, ^21^ The ^11^C‐labeled KOR antagonist [^11^C]‐**5** ([^11^C]LY2795050) was used successfully in several human studies.^22^, ^23^, ^24^, ^25^, ^26^ Due to the short half‐life of carbon‐11 (*t*_1/2_=20 min), fluorine‐18 (*t*_1/2_=110 min) labeled PET tracers were developed as well. In 2017 the synthesis of KOR antagonist [^18^F]‐**6** ([^18^F]LY2459989) via a iodonium ylide was reported. In first in‐man studies (4 healthy male subjects), [^18^F]‐**6** displayed favorable kinetics and binding characeteristics.^27^, ^28^, ^29^

In addition to the diphenyl ether based KOR antagonists **5** and **6**, JDTic derived PET tracers [^11^C]‐**7** and [^18^F]‐**8** were synthesized and biologically evaluated. In mice, carbon‐11‐labeled KOR antagonist [^11^C]‐**7** was able to cross the blood‐brain‐barrier and accumulate in regions with high KOR density. Binding of [^11^C]‐**7** was reduced by potent KOR ligands, such as U‐50,488 and naltrindole.^30^ Although the distribution of fluoropropyl JDTic [^18^F]‐**8** correlates nicely with known KOR localization, a significant specific binding of [^18^F]‐**8** at KOR was not observed. Moreover, [^18^F]‐**8** was rapidly metabolized^31^, ^32^ (Figure 2).

In a recent project,^13^ we demonstrated the promising activity of **1b** and the corresponding fluoroethyltriazole derivative **2**, even *in vivo* in EAE mouse model. Therefore, the biological properties of the compound class of cis,trans‐configured perhydroquinoxalines should be further exploited. Since it was postulated that immune cells were activated predominantly in the periphery, KOR agonists with higher polarity, e. g. bearing an additional hydroxy moiety in the pyrrolidine ring (**3**, R^2^=OH) should be synthesized and pharmacologically evaluated. Additionally, fluorinated PET tracers will be developed. It was planned to introduce fluorine‐18 either in the side chain at 4‐position as in compound **2** or at the pyrrolidine ring as shown in compound **3** (R^2^=F; Figure 1).

## 2 Synthesis

**Scheme 1:**
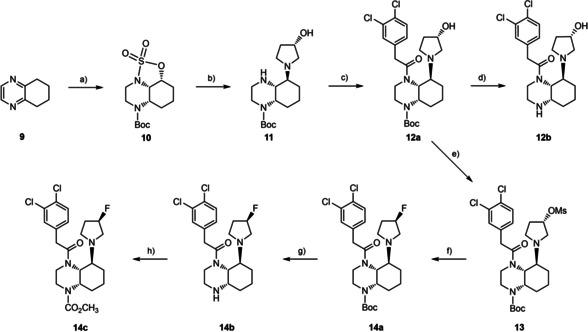
Synthesis of hydroxylated and fluorinated pyrrolidine derivatives **12a**,**b** and **14a**–**c**. a) 7 steps;^13^ b) (*S*)‐3‐hydroxpyrrolidine, K_2_CO_3_, DMF, 80 °C, 16 h, 81 %; c) 2‐(3,4‐dichlorophenyl)acetyl chloride, *i*Pr_2_NEt, CH_2_Cl_2_, 0 °C→RT, 16 h, 85 %; d) F_3_CCO_2_H, CH_2_Cl_2_, RT, 16 h, 87 %; e) H_3_CSO_2_Cl (MsCl), NEt_3_, DMAP, CH_2_Cl_2_, 0 °C→RT, 17 h, 81 %; f) Bu_4_NF, H_2_O, 45 °C, 16 h, 50 %; g) F_3_CCO_2_H, CH_2_Cl_2_, RT, 17 h, 77 %; h) ClCO_2_CH_3_, pyridine, CH_2_Cl_2_, 0 °C→RT, 1 h, 18 %; Only one enantiomer of the racemic mixture **10** and only one diastereomer of diastereomeric mixtures **11** ‐ **14** are shown. The configuration of C‐3 of the pyrrolidine ring is defined: *S* configuration of hydroxy and mesyloxy derivatives **11**–**13** and *R* configuration of fluoro derivatives **14a**–**c**.

For the synthesis of hydroxy and fluoro substituted KOR agonists of type **3**, the synthetic route previously reported by us to obtain *cis*,*trans*‐configured perhydroquinoxalines was pursued.^13^, ^33^, ^34^ At first, the cyclic sulfuric acid ester amide **10** was prepared in seven steps starting from tetrahydroquinoxaline **9**.^13^ Nucleophilic ring opening of **10** by (*S*)‐3‐hydroxypyrrolidine led to secondary amine **11**, which was acylated with 2‐(3,4‐dichlorophenyl)acetyl chloride to afford the amide **12a**. Removal of the Boc‐protective group with trifluoroacetic acid (TFA) provided the secondary amine **12b** (Scheme 1).

In order to obtain fluorinated derivatives, the alcohol **12a** was transformed into the mesylate **13**. Nucleophilic substitution of mesylate **13** with fluoride (Bu_4_NF) provided the fluoropyrrolidine derivative **14a**. Cleavage of the Boc‐protective group with TFA led to secondary amine **14b**, which was acylated with methyl chloroformate to obtain the carbamate **14c** (Scheme 1).

**Scheme 2:**
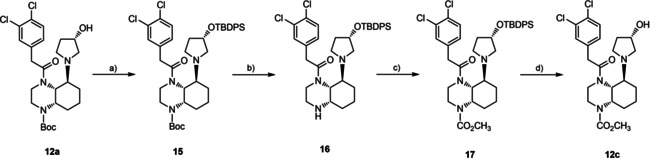
Synthesis hydroxypyrrolidine derivative **12c**. a) TBDPSCl (*tert*‐butyl‐diphenyl‐silyl chloride), imidazole, DMAP, CH_2_Cl_2_, RT, 16 h, 74 %; b) F_3_CCO_2_H, CH_2_Cl_2_, RT, 16 h, 89 %; c) ClCO_2_CH_3_, pyridine, CH_2_Cl_2_, 0 °C→RT, 1 h, 78 %; d) Bu_4_NF, THF, RT, overnight, 64 %. Only one diastereomer of diastereomeric mixtures **12** and **15**–**17** is shown. The *S* configuration of C‐3 of the pyrrolidine ring is defined by the reagent (*S*)‐3‐hydroxypyrrolidine.

For the synthesis of hydroxypyrrolidine derivative **12c** with a methoxycarbonyl moiety the OH moiety of **12a** was protected as TBDPS ether **15**. Removal of the Boc‐protective group with TFA and subsequent acylation of **16** with methyl chloroformate afforded the methoxycarbonyl derivative **17**, which was reacted with Bu_4_NF to remove the silyl protective group. The desired alcohol **12c**, which should also serve as precursor in the radiosynthesis, was obtained in 33 % yield starting from **12a** (Scheme 2).

## 3 Receptor Affinity, Selectivity and Activity

### 3.1 KOR affinity

The KOR affinity of the hydroxylated and fluorinated perhydroquinoxalines **12** and **14** was studied in competitive receptor binding assays. In brief, the test compounds compete with the potent and KOR‐selective radioligand [^3^H]U‐69,593 for a limited number of KORs in a membrane preparation from guinea pig brains. The radioactivity bound by the receptor correlates with the KOR affinity of the test compounds.^35^, ^36^, ^37^, ^38^ The KOR affinity of the hydroxylated and fluorinated ligands **12** and **14** as well as the unsubstituted ligands **1** and some reference compounds is summarized in Table 1.

**Table 1:** Affinities of hydroxylated and fluorinated perhydroquinoxalines and reference compounds towards KOR and related receptors.

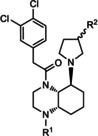							
Compd.	R^1^	R^2^	K_i_±SEM [nM]/% inhibition at 1 μM^[a,b]^				
			KOR	MOR	DOR	σ_1_	σ_2_
			[^3^H]U‐69,593	[^3^H]DAMGO	[^3^H]DPDPE	(+)‐[^3^H]pentazocine	[^3^H]DTG
**1a^13^**	CO_2_C(CH_3_)_3_	H	3.8±1.2	260	438	12 %	16 %
**1b^13^**	H	H	0.54±0.15	28 %	205	0 %	5 %
**1c^13^**	CO_2_CH_3_	H	1.3±0.4	0 %	250	0 %	8 %
**12 a**	CO_2_C(CH_3_)_3_	(*S*)−OH	16±2	9 %	5 %	0 %	0 %
**12 b**	H	(*S*)−OH	2.0±0.5	29 %	246	2 %	0 %
**12 c**	CO_2_CH_3_	(*S*)−OH	9.2±6.0	5 %	693	0 %	5 %
**14 b**	H	(*R*)−F	1.5±0.4	2 %	251	0 %	0 %
**14 c**	CO_2_CH_3_	(*R*)−F	0.82±0.69	0 %	12 %	8 %	962
U‐50,488			0.34±0.07	–	–	–	–
Naloxone			7.3±0.40	2.3±1.1	103	–	–
Morphine			–	5.2±1.6	–	–	–
SNC80			–	–	1.2±0.5	–	–
(+)‐pentazocine			–	–	–	5.4±0.5	–
Haloperidol			–	–	–	6.6±0.9	78±2.3

[a] A value in % reflects the inhibition of the radioligand binding at a test compound concentration of 1 μM. *K*_i_ values without SEM values represent the mean of two experiments (*n*=2) and *K*_i_ values with SEM values represent the mean of three experiments (*n*=3). [b] Guinea pig brain membrane preparations were used in the KOR, MOR and σ_1_ assay. In the DOR assay rat brain and in the *σ*_2_ assay rat liver membrane preparations were used.

The KOR affinity of the hydroxylated and fluorinated pyrrolidines **12** and **14** is in the low‐nanomolar range. However, a Boc group at the quinoxaline ring system (**12 a**: *K*_i_=16 nM) seems to be less tolerated by KOR than a methoxycarbonyl moiety or a proton. Although the *K*_i_ values are below 10 nM, the alcohols **12b** and **12c** are less active than the fluoro derivatives **14b** and **14c** and the unsubstituted analogs **1b** and **1c**. A F atom at the pyrrolidine ring is well tolerated as indicated by the comparable KOR affinities of H/F pairs (e. g., *K*_i_ *(* **14c**)=0.82 nM, *K*_i_ *(* **1c**)=1.3 nM). Altogether, KOR agonists **1** without a substituent at the pyrrolidine ring show the same KOR affinity as the corresponding F derivatives **14**, but the OH derivatives **12** are slightly less active. With exception of **14 b**/**c**, the secondary amines (**1b**,**12b**) show higher KOR affinity than the methoxycarbonyl derivatives **1c** and **12c**.

### 3.2 Selectivity over MOR, DOR and σ receptors

The affinity towards related opioid receptors MOR and DOR was analyzed in receptor binding studies. In order to broaden the selectivity profile, the affinity to σ receptors was also investigated. In particular, in the class of ethylenediamine KOR agonists variation of the stereochemistry or reduction of the phenylacetamide to a phenylethylamine structural element can change the receptor profile towards σ affinity.^39^, ^40^ Membrane preparations from guinea pig brain were used in the MOR and σ_1_ assay, whereas preparations from rat brain were employed in the DOR assay. For investigation of σ_2_ affinity rat liver homogenates were used. The following radioligands were employed for selective labeling of the respective receptor: [^3^H]DAMGO (MOR), [^3^H]DPDPE (DOR), (+)‐[^3^H]pentazocine (σ_1_ receptor) and [^3^H]di‐*o*‐tolylguanidine+(+)‐pentazocine (σ_2_ receptor).^35^, ^36^, ^37^, ^38^, ^41^, ^42^, ^43^

In Table 1 the interaction of the hydroxy and fluoro derivatives **12** and **14** with related receptors is summarized. The complete competition curves were only recorded, when the specific binding of the radioligand at a test compound concentration of 1 μM was reduced by more than 50 %. In case the specific binding of the radioligand was not reduced by more than 50 %, the inhibition of radioligand binding [%] at a test compound concentration of 1 μM is given in Table 1.

The hydroxy‐ and fluoropyrrolidines **12** and **14** did not show remarkable affinity towards MOR, σ_1_ and σ_2_ receptors indicating high selectivity for KOR over MOR, σ_1_ and σ_2_ receptors. A slight DOR affinity was recorded for the secondary amines **12b** (*K*_i_=246 nM) and **14b** (*K*_i_=251 nM). However, both compounds exhibit a KOR:DOR selectivity of more than 100‐fold. The fluoro derivative **14c**, a non‐radioactive counterpart of a potential fluorine‐18‐labeled PET tracer, displays a more than 1000‐fold selectivity over the related MOR, DOR and σ receptors.

### 3.3 Functional activity

In order to further explore the pharmacological potential of quinoxaline‐based KOR agonists **12** and **14**, the agonistic activity at KOR was investigated. The inhibition of cAMP production by the agonists was investigated in a cAMP assay (human HEK293T cells).^44^ A Tango assay (human HTLA cells)^45^ was used to study β‐arrestin‐2 recruitment. Both assays are complementary, since they address two different pathways activated by KOR agonists. A potential bias for one of these pathways could be detected. The results are summarized in Table 2.

**Table 2:** Activity of selected KOR agonists correlated with KOR affinity.

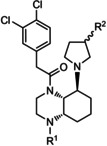							
compd.	R^1^	R^2^	KOR^[a]^	cAMP^[b]^		β‐Arrestin‐2^[c]^	
			[^3^H]U‐69,593				
			K_i_±SEM [nM]	EC_50_ [nM]	*E*_max_ [%]^[d]^	EC_50_ [nM]	*E*_max_ [%]^[d]^
**2^13^**	CH_2_‐triazole‐CH_2_CH_2_F	H	5.6±0.6	2.8	103	43	86
**12 b**	H	(*S*)−OH	2.0±0.5	0.20	102	16	97
**12 c**	CO_2_CH_3_	(*S*)−OH	9.2±6.0	0.19	100	8.8	82
**14 b**	H	(*R*)−F	1.5±0.4	0.033	100	6.9	91
U‐50,488			0.97±0.40	0.013	100	8.0	100

[a] Guinea pig brain membrane preparations. [b] Human HEK 293T cells. [c] Human HTLA cells. [d] The *E*_max_ values refer to U‐50,488 (100 % intrinsic activity).

In the cAMP assay, the perhydroquinoxalines **12b** and **12c** with OH moiety were approximately ten times less active than U‐50,488, whereas the fluoro derivative **14b** shows almost the same EC_50_ value as U‐50,488. All three test compounds represent full agonists with intrinsic activity of 100 % (referred to U‐50,488).

In the β‐arrestin‐2 recruitment assay, the test compounds **12b**, **12c** and **14b** revealed almost the same EC_50_ values as U‐50,488. However, **12c** and **14b** reached only 82 and 91 % of the full agonistic activity of U‐50,488.

Referring the activity of the test compounds **12b**, **12c** and **14b** to the activity of the full agonist U‐50,488 in both assays does not indicate any preference for one of the two pathways, that is, no bias for the G protein or β‐arrestin recruitment pathway could be detected.

## 4 Anti‐inflammatory Activity of the Quinoxaline‐Based KOR Agonists in Vitro

Anti‐inflammatory effects of KOR agonists are mainly based on the inhibition of immune cell activation or the downregulation of cell proliferation. Accordingly, it is well known that KOR agonists prevented the maturation of antigen‐presenting cells resulting in an impaired effector T cell priming.^46^ We were able to demonstrate that the newly developed KOR agonist **2** significantly decreased the expression of classical activation markers and the secretion of the pro‐inflammatory cytokines interferon‐gamma (IFN‐γ), tumor‐necrosis‐factor‐alpha (TNF‐α), and interleukin 17 A (IL‐17A) in mouse as well as human immune cell subsets. In addition, fluoroethyltriazole **2** had strong immunomodulatory capacities as besides down‐regulating pro‐inflammatory cytokines it was able to increase the production of IL‐10, suggesting that **2** contributed to the switch of immune cells from an effector towards a suppressor phenotype. In line with this, **2** significantly decreased diseases severity and delayed disease onset in a mouse model of experimental autoimmune encephalomyelitis, which was associated with downregulated numbers of effector T cells and upregulated levels of regulatory T cells in **2** treated mice compared to controls.^13^

Having confirmed the anti‐inflammatory and immunomodulatory properties of *cis*,*trans‐*perhydroquinoxaline‐based KOR agonists in general, we now intended to characterize more polar substances belonging to this class of compounds. For this purpose, we treated primary mouse or human T cells and dendritic cells (DCs) with **12b**, **12c**, **14b** and **14c** after the cells had been activated with phorbol 12‐myristate 13‐acetate (PMA) in combination with ionomycin (PMA/Iono) to assess the anti‐inflammatory properties of the compounds. The onset and perpetuation of many systemic inflammatory diseases usually involve the activation of antigen‐presenting cells like DCs, which get into contact with exogenous or endogenous triggers and upon maturation, migrate to secondary lymphoid organs, where they induce the priming of pathogenic effector cells, mainly T cells.^47^, ^48^ DC activation and maturation is characterized by the upregulation of CD11c, the induction of costimulatory receptors of the TNF‐ and B7 families including CD80, CD86 or CD40 and the secretion of pro‐inflammatory cytokines such as IFN‐γ, IL‐12, IL‐23 or IL‐6.^49^, ^50^

**Figure 3:**
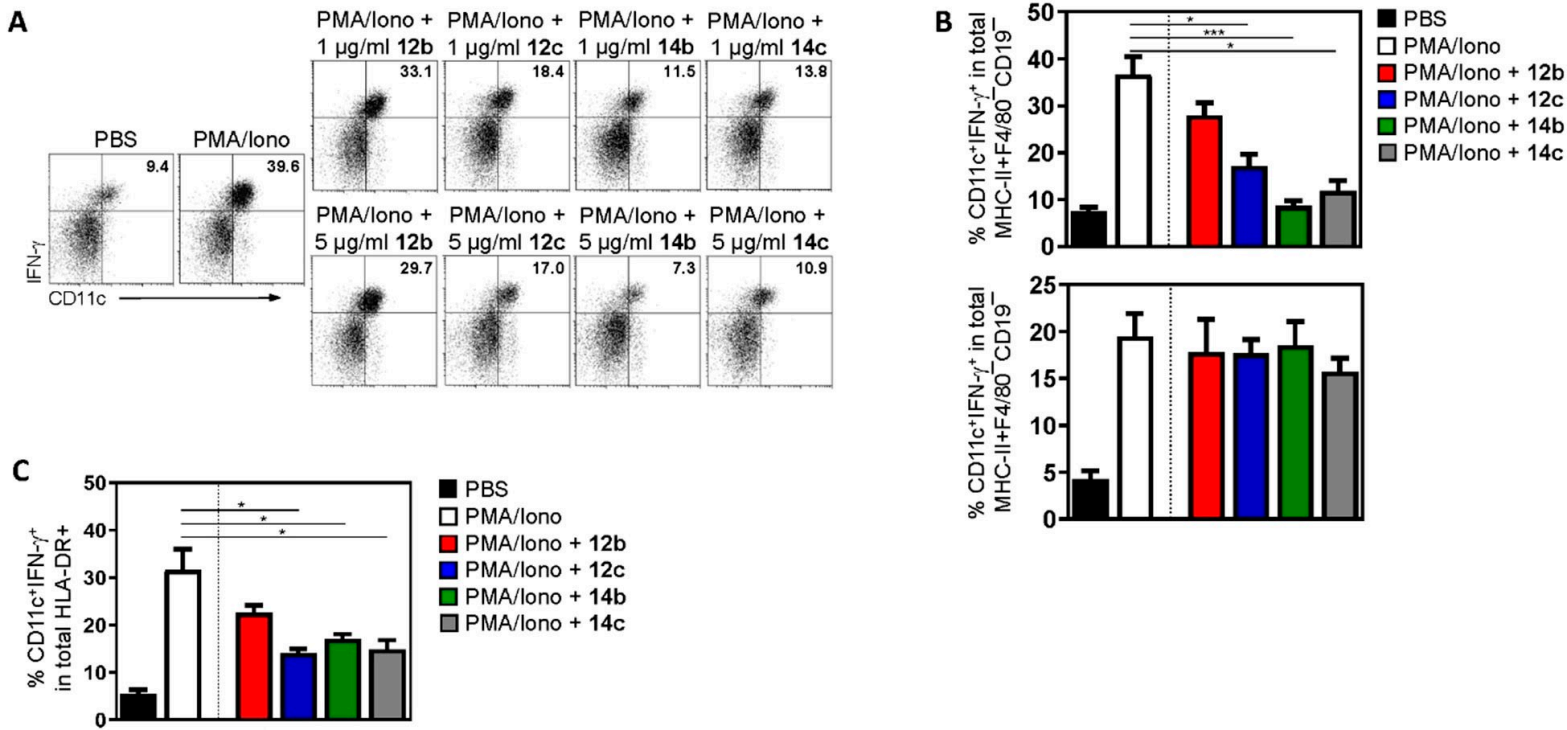
Compounds **12c**, **14b** and **14c** prevented DC maturation and activation. A), B) Mouse DCs were generated from bone marrow precursors in the presence of GM‐CSF and IL‐4, activated with PMA and ionomycin (PMA/Iono) for 12 h, and stimulated with **12b**, **12c**, **14b** or **14c** in a concentration of 1 or 5 μg/mL for an additional 48 h. Control cells received an equal amount of PBS. Representative dot‐plots (A) and percentages of cells expressing the maturation marker CD11c as well as the pro‐inflammatory cytokine IFN‐γ after stimulation with 5 μg/mL of the KOR agonists (B) are shown. Data from *n*=4 wild‐type (top) or *n*=4 KOR‐deficient mice (bottom) are depicted. Cells are gated on MHC−II^+^F4/80^−^CD19^−^ DCs, and IFN‐γ staining was performed after cell permeabilization. Data are presented as mean ±SD; * *p*<0.05 and *** *p*<0.01 vs. PMA/Iono treatment. C) Human DCs were purified from the peripheral blood of healthy donors and stimulated with PMA/Iono for 12 h. Subsequently, cells were incubated with compounds **12b**, **12c**, **14b**, and **14c** (5 μM/mL each) for an additional 48 h or received an equal amount of PBS. The percentages of cells expressing CD11c and IFN‐γ from *n*=4 healthy human donors are shown. Cells are gated on HLA‐DR, and IFN‐γ staining was performed after cell permeabilization. Data are presented as mean ±SD; * *p*<0.05 vs. PMA/Iono stimulation.

Accordingly, we observed markedly increased levels of CD11c and IFN‐γ in PMA/ionomycin‐stimulated mouse DCs compared to non‐activated controls (Figure 3A). However, when the activated DCs were treated with **12c**, **14b** or **14c**, the expression of the maturation marker CD11c and the IFN‐γ production were significantly reduced, indicating an anti‐inflammatory effect of these three compounds, whereas **12b** did non modulate the CD11c or IFN‐γ expression. Worth mentioning that **14b** seemed to be more potent than **12c** or **14c** (Figure 3A and B) and that the effect of all three compounds was concentration‐dependent because a more pronounced reduction in DC activation and maturation was observed when the cells were treated with 5 μg/mL instead of 1 μg/mL of the KOR agonists. Interestingly, the anti‐inflammatory capacity of all three compounds was clearly mediated by binding to KOR since the effect was completely abrogated when DCs from KOR‐deficient mice were stimulated with **12c**, **14b** or **14c** (Figure 3B). Next, we assessed whether the KOR agonists were also able to modulate the activation and maturation of human DCs and therefore, purified HLA‐DR^+^ cells from peripheral blood of healthy human donors. As shown in Figure 3C, **12c**, **14b** and **14c** reduced the expression of CD11c and the production of IFN‐γ in human DCs as well, whereas compound **12b** seemed to have only a minor but not significant anti‐inflammatory effect on human antigen‐presenting cells (Figure 3C).

**Figure 4:**
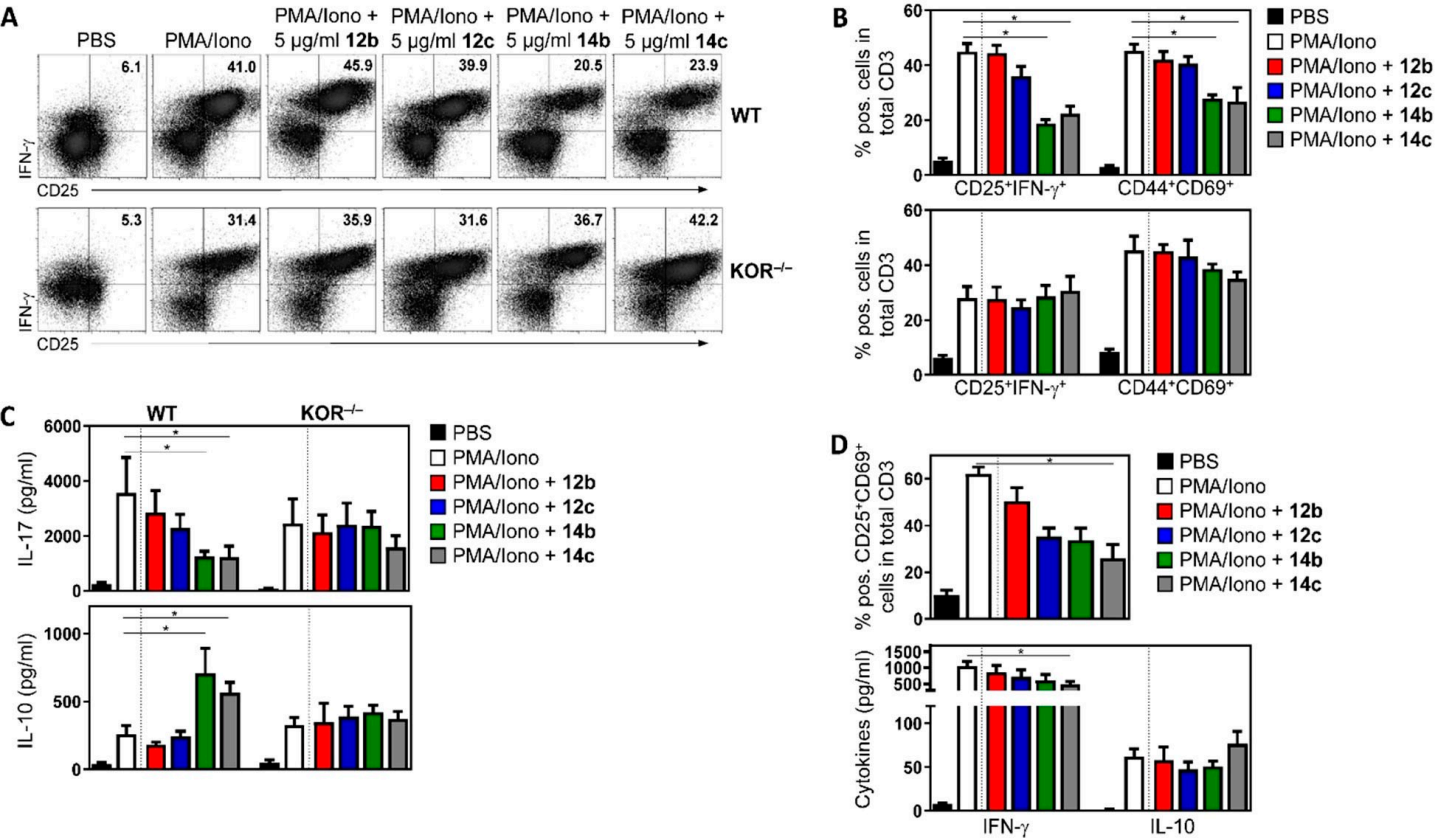
Anti‐inflammatory and immunomodulatory effects of **14b** and **14c** in T cells. A), B) Mouse T cells were purified from the peripheral lymph nodes of wild‐type (WT) or KOR‐deficient mice (KOR^−/−^), activated with PMA and ionomycin (PMA/Iono) for 12 h, and stimulated with **12b**, **12c**, **14b** or **14c** at a concentration of 5 μg/mL for an additional 48 h. Control cells received an equal amount of PBS. Representative dot‐plots (A) and percentages of cells expressing the typical activation markers CD25, CD69, CD44 as well as the pro‐inflammatory cytokine IFN‐γ (B) are shown. Data from *n*=4 WT (B, top) or *n*=4 KOR^−/−^ mice (B, bottom) are depicted. Cells are gated on CD3, and IFN‐γ staining was performed after cell permeabilization. Data are presented as mean ±SD; * *p*<0.05 vs. PMA/Iono treatment. C) Cytokine quantification in cell culture supernatants from T cells of WT and KOR^−/−^ after activation with PMA/Iono and stimulation with compounds **12b**, **12c**, **14b** or **14c**. Data from *n*=3 mice per group are shown (* *p*<0.05 vs. PMA/Iono). D) Percentages of CD25^+^CD69^+^ cells (top) and cytokine expression (bottom) in total CD3^+^ T cells from *n*=4 healthy human donors. IFN‐γ staining (top) was performed after cell permeabilization, and cytokine quantification in cell culture supernatants (bottom) was done using the Legend‐Plex bead‐based immunoassay. Data are presented as means ±SD; * *p*<0.05 vs. PMA/Iono.

After entering secondary lymphoid organs, activated DC get into contact to naive T cells, resulting in the priming of effector cells (T helper or cytotoxic T cells). Effector T cells including Th1 or Th17 cells migrate to the site of inflammation and in case of infection, contribute to the elimination of bacteria or virus‐containing cells. Under sterile inflammatory conditions (e. g., in individuals with multiple sclerosis, psoriasis or rheumatoid arthritis), Th1 and Th17 are thought to mediate tissue destruction.^51^, ^52^, ^53^ Besides the upregulation of surface markers like CD25, CD44 or CD69, these cells are characterized by the expression of pro‐inflammatory cytokines including IFN‐γ, TNF‐α or IL‐17. To quantify the anti‐inflammatory effect of **12b**, **12c**, **14b** and **14c** in T cells, total T cells were purified from mouse peripheral lymph nodes, activated with PMA plus ionomycin, and stimulated with the KOR agonists in a concentration of 5 μg/mL. In contrast to our observations in activated DC, only **14b** and **14c** exhibited a potent anti‐inflammatory capacity by significantly reducing the expression of the surface activation markers CD25, CD44 and CD69 as well as the IFN‐γ production (Figure 4A and B), whereas **12b** and **12c** did not modulate the activation status of mouse T cells. Of note, also in T cells the effect of **14b** and **14c** was mediated by binding to KOR because treatment of T cells from KOR‐deficient mice did not result in an altered expression of activation markers or cytokine release (Figure 4B and C). Interestingly, **14b** and **14c** might not only have anti‐inflammatory properties but also immunomodulatory capacities since besides the downregulated IFN‐γ and IL‐17 levels in cell cultures stimulated with these KOR agonists, we detected a significant upregulation of the anti‐inflammatory cytokine IL‐10 (Figure 3C). This observation could possibly indicate that compounds **14b** and **14c** might have induced a switch from effector to regulatory T cells, which is in line with published data. Liang et al. described that opioid receptor agonists are able to modulate immune cell functions by inhibiting NF‐κB activation resulting in increased IL‐10 levels.^46^ Worth mentioning that the immunomodulatory capacity of compounds **14b** and **14c** was not an off‐target effect but dependent on binding to KOR because we did not observe any impact of these KOR agonists on the cytokine secretion in activated T cells from KOR‐deficient mice (Figure 4C).

Next, we assessed the anti‐inflammatory and immunomodulatory capacity of **14b** and **14c** in human T cells and therefore, purified total T cells from peripheral blood of four healthy donors. As shown in Figure 4D, only compound **14c** was able to significantly reduce the expression of typical surface activation markers such as CD25 and CD69 and moreover, to decrease the IFN‐γ secretion. However, the stimulation of human T cells with **14c** only slightly but not significantly induced IL‐10 (Figure 4D), indicating that at least the anti‐inflammatory effect of the KOR agonist **14c** was not limited to mouse cells but also detectable in human primary T cells.

## 5 Radiosynthesis and Biological Evaluation of KOR‐PET tracers [^18^F]‐2 and [^18^F]‐14c

With the long‐term goal of better understanding the role of KOR in MS, a KOR selective PET tracer should be developed. Some of the most important characteristics for a promising KOR PET tracer are high KOR affinity and high selectivity over related receptors. Moreover, the introduction of the radioactive atom in an easy and fast way compatible with the half‐life of the radionuclide at the end of the synthesis should be possible. According to these requirements the fluorinated KOR agonists **2** (*K*_i_=5.6 nM) and **14c** (*K*_i_=0.82 nM) represent suitable candidates to be developed as PET tracers.

### 5.1 Radiosynthesis, pharmacokinetic properties and *in vivo* biodistribution of [^18^F]‐2

**Scheme 3:**
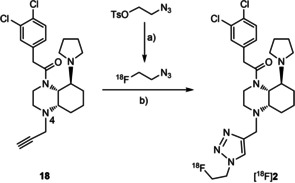
Two‐step radiosynthesis of fluorinated PET tracer [^18^F]‐**2**. a) K[^18^F]F, K222, K_2_CO_3_, CH_3_CN, 110 °C, 3 min; b) CuSO_4_, Na ascorbate, DMF, H_2_O, 40 °C, 30 min. [^18^F]‐**2**: rcy: 49.9±8.6 % (*n*=5, d,c.); total synthesis time: 136±12 min; rcp: 99.3±0.4 %; *A*_m_: 4.6–14.6 GBq/μmol.

The fluorinated PET tracer [^18^F]‐**2** was prepared in a two‐step radiosynthesis. (Scheme 3) In the first step, [^18^F]‐1‐azido‐2‐fluoroethane was prepared by nucleophilic substitution (S_N_2) of 2‐azidoethyl 4‐methylbenzenesulfonate with K[^18^F]F. Subsequent copper(I)‐catalyzed 1,3‐dipolar cycloaddition of the fluorine‐18‐labeled intermediate with the alkyne precursor **18** 13 provided the desired product [^18^F]‐**2**. [^18^F]‐**2** was purified by preparative HPLC and finally formulated as water/EtOH (9 : 1, *v/v*) solution. The triazole [^18^F]‐**2** was obtained in radiochemical yields (rcy) of 49.9±8.6 % (*n*=5, decay‐corrected, based on cyclotron‐derived [^18^F]fluoride ions (d.c.)). The total synthesis time was 136±12 min from the end of radionuclide production up to final formulation. [^18^F]‐**2** was isolated with radiochemical purities (rcp) of 99.3±0.4 % and a molar activities (*A*_m_) of 4.6–14.6 GBq/μmol. (Scheme 3)

The distribution coefficient at pH 7.4 (logD_7.4_) was determined by a shake flask method using the radioligand [^18^F]‐**2**. After distribution of [^18^F]‐**2** between *n*‐octanol and phosphate buffered saline (pH 7.4) layers, the radioactivity in both layers was determined and set in ratio. According to this procedure, a logD_7.4_ value of 1.37±0.06 was determined indicating a moderate lipophilic compound, which should be able to penetrate into the central nervous system.

Enzymes present in blood serum could be able to transform the radioactive PET tracer [^18^F]‐**2**. Therefore, the blood serum stability of [^18^F]‐**2** was investigated before testing the tracer *in vivo*. [^18^F]‐**2** was incubated with mouse and human serum for 90 min at 37 °C. Even after 90 min, radio‐HPLC did not show any additional radioactive compound. Only the parent tracer [^18^F]‐**2** could be detected indicating high stability in mouse and human blood serum (Figure S1 in Supporting Information).

**Figure 5:**
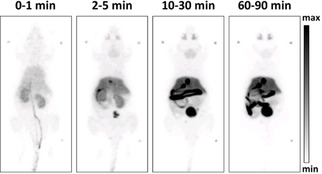
In vivo biodistribution analysis of radioactivity in an adult C57BL/6 mouse after intravenous injection of [^18^F]‐**2**. Maximum intensity projections of the biodistribution at indicated time points post injection.

**Figure 6:**
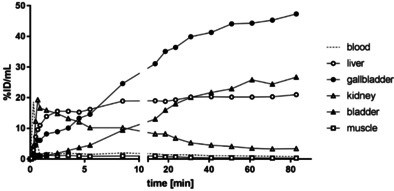
In vivo biodistribution of radioactivity in an adult C57BL/6 mouse after intravenous injection of [^18^F]‐**2**. Time–activity concentration curves illustrate tracer dynamics in selected regions of interest (ROI). Activity is displayed as percentage of injected dose per volume during the whole observation period of 90 min.

Next, the distribution of the PET tracer [^18^F]‐**2** in mice was investigated. For this purpose, [^18^F]‐**2** was injected into 15‐week‐old C57BL/6 mice. Figure 5 displays representative maximum intensity projections at indicated time points after injection of [^18^F]‐**2**. A very fast and efficient clearance of [^18^F]‐**2** from the body was observed following both hepatic and renal pathways. Already after 2 min the radioactivity was almost completely found in liver, kidneys and bladder. In the liver, the radioactivity remained almost constant over the period of 90 min (Figure 6). Besides a transient local increase in radioactivity in the submandibular and harderian glands, no significant accumulation of [^18^F]‐**2** in non‐excreting organs such as heart, lung and muscles as indicator for unspecific binding could be detected. Furthermore, increased radioactivity in the bones was not observed excluding a fast defluorination of [^18^F]‐**2**.

The PET images do not show any enrichment of radioactivity in the brain or spinal cord at any time point. Obviously, [^18^F]‐**2** was not able to target KOR in the central nervous system. This behavior can be attributed to low penetration of the blood‐brain barrier, fast elimination kinetics or a very fast *in vivo* biotransformation of [^18^F]‐**2**.

In order to investigate the *in vivo* biotransformation of [^18^F]‐**2**, the blood of three mice 90 min after injection of [^18^F]‐**2** was pooled and analyzed. The radio‐HPLC showed 90 % of the intact PET tracer [^18^F]‐**2** and 10 % of a more polar radiometabolite (Figure S2).

It can be concluded that a very fast biotransformation is not the reason for missing accumulation of [^18^F]‐**2** in regions with high KOR density, for example, spinal cord. The logD_7.4_ value of 1.37 is in a promising range for penetration the blood brain barrier. Therefore, it is assumed that the very fast elimination *via* liver and kidneys is responsible for the unfavorable imaging properties of [^18^F]‐**2**.

### 5.2 Radiosynthesis, pharmacokinetic properties and *in vivo* biodistribution of [^18^F]‐14c

**Scheme 4:**
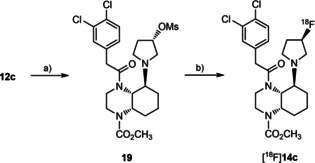
Radiosynthesis of fluorinated PET tracer [^18^F]‐**14c** by direct nucleophilic substitution. a) CH_3_SO_2_Cl, NEt_3_, DMAP, CH_2_Cl_2_, 0 °C, 30 min, then RT, 16 h, 55 %; b) K[^18^F]F, K222, K_2_CO_3_, CH_3_CN, 110 °C, 20 min. [^18^F]‐**14c**: rcy: 20.7±3.4 % (*n*=5, d.c.); total synthesis time: 128±14 min; rcp: 98.9±0.7 %; *A*_m_: 4.0–111.3 GBq/μmol.

In order to obtain a KOR‐PET tracer with better pharmacokinetics the fluoropyrrolidine derivative **14c** showing even higher KOR affinity (*K*_i_=0.82 nM) was considered to be prepared in a radioactive form. For this purpose, the synthesis was changed in order to introduce [^18^F]fluoride at the very end of the synthesis. (Scheme 4). At first, the alcohol **12c** was converted into the mesylate **19** upon reaction with mesyl chloride. Subsequent nucleophilic substitution of **19** with K[^18^F]F in the presence of the cryptand K222 provided the fluorinated PET tracer [^18^F]‐**14c** in a radiochemical yield of 20.7±3.4 % (n=5, decay corrected) within a total synthesis time of 128±14 min up to formulation. The radiochemical purity was 98.9±0.7 %. (Scheme 4)

The logD_7.4_ value of [^18^F]‐**14c** was recorded as described above for [^18^F]‐**2**. The fluoropyrrolidine [^18^F]‐**14c** displayed a higher lipophilicity than [^18^F]‐**2** with a 15‐fold higher concentration of [^18^F]‐**14c** in the *n*‐octanol layer than [^18^F]‐**2** resulting in a logD_7.4_ value of 2.54±0.22. However, this value is still in a promising range for reaching the central nervous system.

In order to analyze the serum stability [^18^F]‐**14c** was incubated with human and mouse serum. After an incubation period of 90 min, radio‐HPLC did not show any new radioactive product indicating high stability in both human and mouse serum.

**Figure 7:**
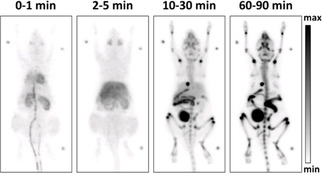
In vivo biodistribution analysis of radioactivity in an adult C57BL/6 mouse after intravenous injection of [^18^F]‐**14c**. Maximum intensity projections of the biodistribution at indicated time points post injection.

The *in vivo* biodistribution of [^18^F]‐**14c** was investigated in 10‐week‐old C57BL/6 mice. As reported for [^18^F]‐**2**, the clearance of [^18^F]‐**14c** from the body was fast following both renal and hepatic pathways. Unfortunately, 10 min after injection, radioactivity in the bones (Ca[^18^F]F_2_) was observed indicating fast defluorination of the compound. The high radioactivity detected 60–90 min after injection of [^18^F]‐**14c** in the joints, the spine and the skull confirmed the extensive defluorination of [^18^F]‐**14c** (Figure 7).

**Figure 8:**
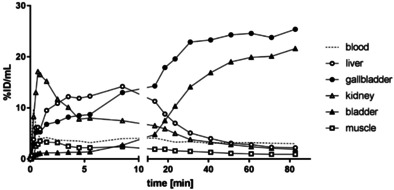
In vivo biodistribution of radioactivity in an adult C57BL/6 mouse after intravenous injection of [^18^F]‐**14c**. Time‐activity concentration curves illustrate tracer dynamics in selected regions of interest (ROI). Activity is displayed as percentage of injected dose per volume during the whole observation period of 90 min.

In Figure 8 the distribution of the radioactivity ([^18^F]‐**14c**) in different organs over the time is shown. Two minutes after injection, radioactivity was found only in liver, kidneys and gallbladder. Interestingly, the radioactivity found in liver decreased after 10 min; whereas the radioactivity for the bladder increased (Figure 8).

90 min after injection of [^18^F]‐**14c**, the animals were sacrificed, and the organs were collected and analyzed *ex vivo* at the γ‐counter to determine the residual radioactivity in the different organs. It was shown that the radioactivity was mainly present in the excretion organs and in the urine (Figure S3).

### 5.3 *In vitro* biotransformation of 14c in the presence of mouse liver microsomes

Due to its defluorination [^18^F]‐**14c** does not represent a good candidate to become a PET tracer for selective imaging KORs in the central nervous system. Therefore, [^18^F]‐**14c** was not further evaluated *in vivo*. However, in order to confirm the defluorination by *in vitro* experiments and to get an idea about possible metabolically labile positons, the non‐radioactive compound **14c** was incubated with mouse liver microsomes and the cofactor NADPH. The stability of **14c** and the formed metabolites were identified by LC‐MS/MS experiments.

**Figure 9:**
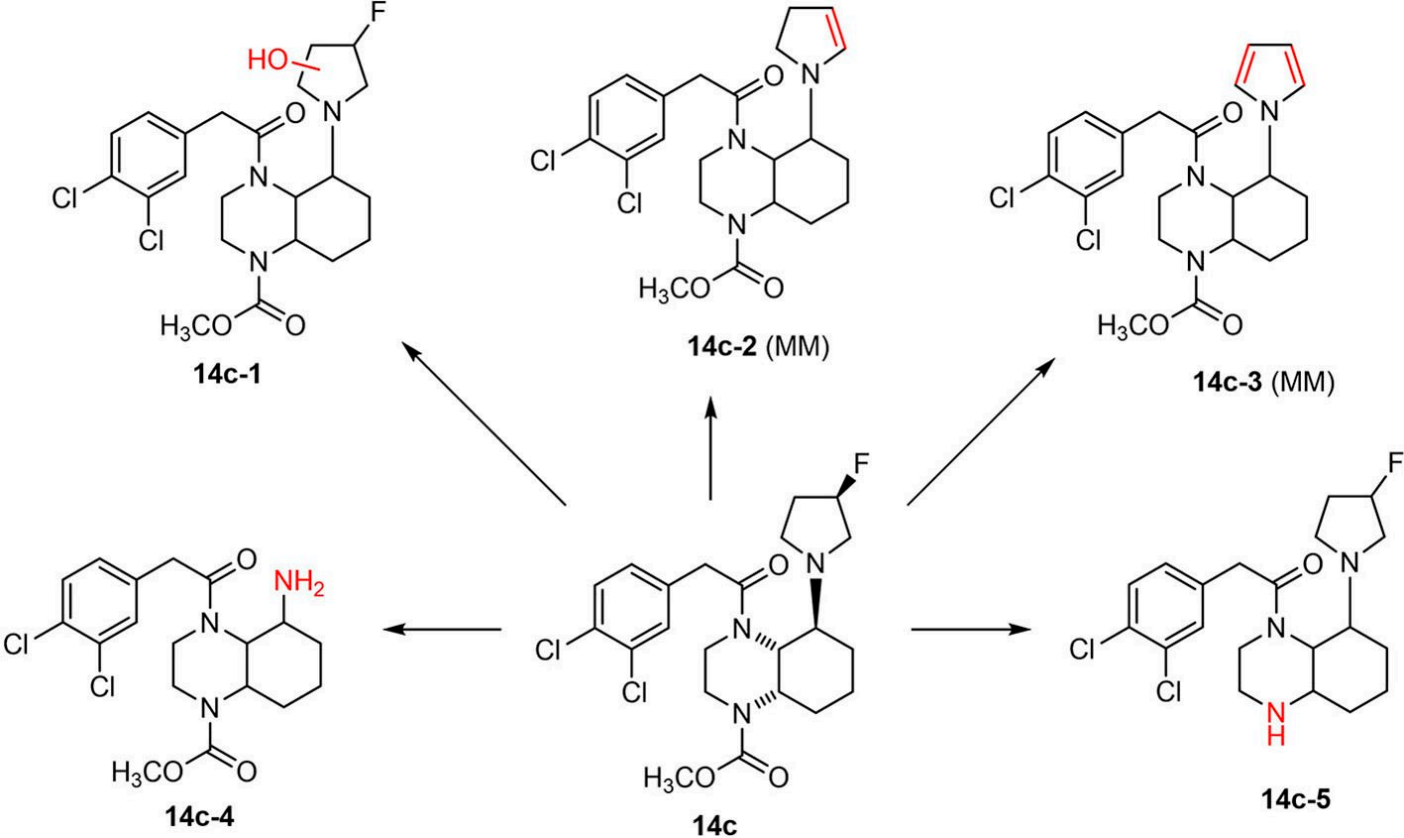
Postulated structures of phase‐I metabolites **14c‐1**–**5** of KOR agonist **14c** produced in vitro using mouse liver microsomes and NADPH. The parent compound **14c** represents a mixture two enantiomerically pure diastereomers, the stereochemistry of the metabolites **14c‐1**–**5** was not determined. MM: main metabolite.

After an incubation period of 90 min, five phase‐I metabolites were identified (Figure 9). Hydroxylation took place at the pyrrolidine ring affording metabolite **14c‐1**. Loss of HF gave dihydropyrrole **14c‐2**, which was most likely further oxidized (aromatization) to provide pyrrole metabolite **14c‐3**. Defluorination by hydroxylation and subsequent elimination of HF to form the respective ketone was already described as defluorination mechanism in literature.^54^ Therefore, it is assumed that parent compound **14c** was hydroxylated at the pyrrolidine ring (e. g., metabolite **14c‐1**) leading to a ketone after loss of HF. The ketone was then reduced to an alcohol, which induced elimination of H_2_O to form dihydropyrrole **14c‐2**. Metabolite **14c‐2** was most likely hydroxylated in α‐position of the dihydropyrrole N atom resulting in aromatization by H_2_O elimination (metabolite **14c‐3**). Whereas the metabolite **14c‐1** initiates elimination of fluoride, the metabolites **14c‐2** and **14c‐3** resulted from direct loss of HF. The high amounts of metabolites **14c‐2** and **14c‐3** (Figure S4) correlate nicely with the defluorination observed *in vivo* during the biodistribution experiments with [^18^F]‐**14c**.

In addition to the metabolites **14c‐1**‐**3**, the primary amine **14c‐4** and the secondary amine **14c‐5** were produced in minor amounts (Figure S4A and B) The primary amine **14c‐4** was formed by double oxidative N‐dealkylation and the secondary amine **14c‐5** by hydrolytic cleavage of the methyl carbamate, whereas hydrolysis of the phenylacetamide was not observed.

## 6 Conclusion

KORs play a crucial role in the pathology of various central nervous system disorders, including pain, depression, anxiety, Multiple Sclerosis, and, furthermore, itching skin diseases. Anti‐inflammatory effects of KOR agonists are mainly based on inhibition of immune cell activation, which predominantly occurs in the periphery.

In this project, we focused on two issues of KOR agonists: on the one hand side polar KOR agonists should be developed to activate KOR in the periphery with the aim to inhibit immune cell activation and reduce centrally mediated side effects. On the other side, a fluorinated PET tracer should be developed, which allows imaging of KOR non‐invasively in healthy and diseased conditions.

In order to reach these aims an S_N_2 substitution of cyclic sulfuric acid derivative **10** with hydroxypyrrolidine was performed. A series of three polar hydroxypyrrolidines **12a**–**c** bearing a Boc group, a proton or a methoxycarbonyl moiety at the second quinoxaline N atom was prepared. Conversion of the OH moiety into a F atom led to fluoropyrrolidines **14a**–**c** with the same substitution pattern at the second quinoxaline N‐atom. The KOR affinity of the hydroxypyrrolidines **12b**,**c** was in the low‐nanomolar range, and those of the fluoropyrrolidines **14b**,**c** in the low‐ to sub‐nanomolar range. Hydroxypyrrolidines **12** and fluoropyrrolidines **14** exhibit high selectivity for KOR over related MOR, DOR, σ_1_ and σ_2_ receptors. In the cAMP and β‐arrestin recruitment assay, the fluoropyrrolidine **14b** was as active as the prototypical KOR agonist U‐50,488, but the hydroxypyrrolidine derivatives **12b** and **12c** were slightly less potent.

The production of inflammation markers CD11c and INF‐γ was increased upon stimulation of mouse dendritic cells with PMA/ionomycin. Treatment of activated dendritic cells with hydroxypyrrolidine **12c** and fluoropyrrolidines **14b** and **14c** led to reduced formation of CD11c and INF‐γ indicating anti‐inflammatory activity. The most polar KOR agonist **12b** did not show this effect, which was attributed to its very high polarity. Worth mentioning that this effect was completely abrogated when stimulated DC from KOR‐deficient mice were treated with **12c**, **14b** and **14c**. This observation proves that the anti‐inflammatory effect was mediated by KOR. The same result was obtained with human DC.

Activated DC are able to activate effector T cells including Th1 and Th17 cells, which reinforce the inflammatory process. Therefore, the effect of KOR agonists **12b**, **12c**, **14b** and **14c** on mouse and human T cells activated with PMA/ionomycin was investigated. Whereas the fluoropyrrolidines **14b** and **14c** led to significantly reduced expression of activation markers CD25, CD44 and CD69 and release of IFN‐γ, the more polar hydroxypyrrolidines **12b** and **12c** were not active. It was shown that these effects were mediated by KOR, since T cells isolated from KOR‐deficient mice could not be modulated by KOR agonists **14b** and **14c**.

The anti‐inflammatory effects of fluoropyrrolidines **14b** and **14c** are mediated by KOR as DC and T cells from KOR‐deficient mice were not influenced by the KOR agonists **14b** and **14c**. The effects of **14b** and **14c** on mouse DC and T cells were also observed for human DC and T cells, indicating a translational potential of the anti‐inflammatory effects of KOR agonists to humans. The hydroxypyrrolidines were either not active (**12b**) or less active (**12c**) in these experiments. This can be attributed to the high polarity of the hydroxypyrrolidines **12b** and **12c** or to the lower KOR affinity and KOR activity compared to the fluoropyrrolidines **14b** and **14c**.

The fluorinated PET tracer [^18^F]**2** was prepared by a 1,3‐dipolar cycloaddition of propargylamine **18** with [^18^F]fluoroethyl azide (Click reaction). Application of tracer [^18^F]‐**2** to mice led to a very fast elimination via kidney and liver. After 2 min, almost the complete radioactivity was found in liver, kidneys and bladder. Although the polarity of [^18^F]‐**2** (logD_7.4_=1.37) was in a promising range, KOR rich regions were not labeled by [^18^F]‐**2**. Fast metabolism could be excluded as reason of the fast elimination.

As second option, the fluorinated PET tracer [^18^F]‐**14c** was prepared by nucleophilic substitution of the mesylate **19** with [^18^F]fluoride. The second PET tracer [^18^F]‐**14c** displayed slightly higher lipophilicity (logD_7.4_=2.54) than [^18^F]‐**2**. In biodistribution studies, [^18^F]‐**14c** exhibited slower elimination than [^18^F]‐**2**, but labeling of bones was observed, which was attributed to defluorination of the PET tracer [^18^F]‐**2**. This defluorination was analyzed *in vitro* using mouse liver microsomes and NADPH/H^+^. Altogether, five metabolites were identified and the main metabolites result from defluorination.

## Experimental Section

### Chemistry

#### General methods

Oxygen and moisture sensitive reactions were carried out under nitrogen, dried with silica gel with moisture indicator (orange gel, Merck) and in dry glassware (Schlenk flask or Schlenk tube). All solvents were of analytical grade quality. Flash chromatography (FC): Silica gel 60, 40–63 μm (Machery Nagel); parentheses include: diameter of the column (Ø), length of the stationary phase (*l*), fraction size (*v*) and eluent. NMR: NMR spectra were recorded on Agilent DD2 400 MHz and 600 MHz spectrometers; chemical shifts (*δ*) are reported in parts per million (ppm) against the reference substance tetramethylsilane and calculated using the solvent residual peak of the undeuterated solvent. HPLC method to determine the purity of compounds (Method Pu): Equipment 1: Pump: L‐7100, degasser: L‐7614, autosampler: L‐7200, UV detector: L‐7400, interface: D‐7000, data transfer: D‐line, data acquisition: HSM‐Software (all from LaChrom, Merck Hitachi); Equipment 2: Pump: LPG‐3400SD, degasser: DG‐1210, autosampler: ACC‐3000T, UV‐detector: VWD‐3400RS, interface: DIONEX UltiMate 3000, data acquisition: Chromeleon 7 (Thermo Fisher Scientific); column: LiChropher® 60 RP‐select B (5 μm), LiChroCART® 250–4 mm cartridge; flow rate: 1.0 mL/min; injection volume: 5.0 μL; detection at λ=210 nm; solvents: A: demineralized water with 0.05 % (*v/v*) trifluoroacetic acid, B: acetonitrile with 0.05 % (*v/v*) trifluoroacetic acid; gradient elution (% A): 0–4 min: 90 %; 4–29 min: gradient from 90 to 0 %; 29–31 min: 0 %; 31–31.5 min: gradient from 0 to 90 %; 31.5–40 min: 90 %. The purity of all test compounds is greater than 95 %.

#### Synthetic procedures

##### *tert*‐Butyl (4a*R*,5*S*,8a*S*)‐ and (4a*S*,5*R*,8a*R*)‐5‐[(*S*)‐3‐hydroxypyrrolidin‐1‐yl]‐3,4,4 a,5,6,7,8,8 a‐octahydroquinoxaline‐1(2*H*)‐ carboxylate (11)

Under a N_2_ atmosphere, (*S*)‐3‐hydroxypyrrolidine (0.25 mL, 3.1 mmol, 5 equiv) was added to a solution of **10** (200 mg, 0.63 mmol, 1 equiv) and K_2_CO_3_ (17 mg, 0.13 mmol, 0.2 equiv) in dry DMF (2 mL). The mixture was stirred at 80 °C for 16 h. Afterwards, HCl (1 M, 5 mL) was added, and the mixture was extracted with CH_2_Cl_2_ (2×10 mL). Then, NaOH (1 M, 5 mL) was added (pH 10) and the aqueous layer was extracted with CH_2_Cl_2_ (2×20 mL). The combined organic layers were dried (Na_2_SO_4_), filtered and concentrated *in vacuo*. The residue was purified by flash column chromatography (Ø=2 cm, *h*=15 cm, CH_2_Cl_2_/CH_3_OH/NH_3_ (25 %) solution 94 : 5 : 1, *V*=12 mL, *R*_f_=0.36 (CH_2_Cl_2_/CH_3_OH/NH_3_ (25 %) 94 : 5 : 1)). Pale yellow oil, yield 165 mg (81 %). C_17_H_31_N_3_O_3_ (325.5 g/mol). ^1^H NMR (600 MHz, CDCl_3_): *δ* (ppm)=1.35–1.50 (m, 2H, 7‐C*H*_2_, 8‐C*H*_2_), 1.43 (s, 9H, CO_2_C(C*H*_3_)_3_), 1.57–1.64 (m, 2H, 6‐C*H*_2_), 1.66–1.80 (m, 2H, 7‐C*H*_2_, NCH_2_C*H*_2_CHOH), 1.86–2.01 (m, 1H, 8‐C*H*_2_), 2.07–2.17 (m, 1H, NCH_2_C*H*_2_CHOH), 2.20–2.38 (m, 3H, 2‐C*H*_2_, 5‐C*H*, NC*H*_2_CH_2_CHOH), 2.46–2.57 (m, 1H, NC*H*_2_CHOH), 2.70–2.78 (m, 1H, 3‐C*H*_2_), 2.78–2.84 (m, 1H, NC*H*_2_CHOH), 2.86–2.94 (m, 1H, NC*H*_2_CH_2_CHOH), 2.95–3.07 (m, 3H, 2‐C*H*_2_, 3‐C*H*_2_, 4a‐C*H*), 3.71–3.81 (m, 1H, −N*H*), 4.15–4.24 (m, 1H, 8a‐C*H*), 4.24–4.32 (m, 1H, NCH_2_C*H*OH). A signal for the OH proton is not seen in the spectrum. ^13^C NMR (151 MHz, CDCl_3_): *δ* (ppm)=19.4 (1 C, C‐7), 22.8 (1 C, C‐8), 24.4 (1 C, C‐6), 28.6 (3 C, CO_2_C(*C*H_3_)_3_, 34.5 (1 C, NCH_2_ *C*H_2_CHOH), 46.5 (1 C, C‐3), 49.2 (1 C, C‐8a), 50.0 (1 C, N*C*H_2_CH_2_CHOH), 50.2 (1 C, C‐2), 56.7 (1 C, C‐4a), 60.5 (1 C, N*C*H_2_CHOH), 65.5 (1 C, C‐5), 71.1 (1 C, NCH_2_ *C*HOH), 79.7 (1 C, CO_2_ *C*(CH_3_)_3_), 155.0 (1 C, *C*O_2_C(CH_3_)_3_). IR (neat): *ν* (cm^−1^)=3437 (OH), 3298 (R_2_NH), 2943 (C−H_aliph_), 1670 (N−C=O), 1165 (N−C). HRMS (APCI): *m/z* 326.2430 (calcd. 326.2438 for C_17_H_32_N_3_O_3_ [*M*+H]^+^).

##### *tert*‐Butyl (4a*R*,5*S*,8a*S*)‐ and (4a*S*,5*R*,8a*R*)‐4‐[2‐(3,4‐dichlorophenyl)acetyl]‐5‐[(*S*)‐3‐hydroxypyrrolidin‐1‐yl]‐3,4,4 a,5,6, 7,8,8 a‐octahydroquinoxaline‐1(2*H*)‐carboxylate (12a)

Diisopropylethylamine (476 mg, 3.7 mmol, 4 equiv) was added to a solution of secondary amine **11** (300 mg, 0.9 mmol, 1 equiv) in CH_2_Cl_2_ (8 mL). At 0 °C a solution of 2‐(3,4‐dichlorophenyl)acetyl chloride (515 mg, 2.3 mmol, 2.5 equiv) in CH_2_Cl_2_ (3 mL) was added dropwise. The mixture was stirred at room temperature for 16 h. Then, NaOH (0.5 M, 10 mL) was added, and the mixture was extracted with CH_2_Cl_2_ (3×15 mL). The combined organic layers were dried (Na_2_SO_4_), filtered and concentrated *in vacuo*. The residue was purified by flash column chromatography (Ø=3 cm, *h*=10 cm, CH_2_Cl_2_/CH_3_OH/NH_3_ (25 %) solution 94 : 5 : 1, *V*=30 mL, *R*_f_=0.50 (CH_2_Cl_2_/CH_3_OH/NH_3_ (25 %) 89 : 10 : 1)). Yellow oil, yield 398 mg (85 %). C_25_H_35_Cl_2_N_3_O_4_ (512.5 g/mol). ^1^H NMR (600 MHz, CDCl_3_): *δ* (ppm)=1.20–1.32 (m, 1H, 8‐C*H*_2_), 1.35–1.56 (m, 2H, 6‐C*H*_2_, 7‐C*H*_2_), 1.45 (s, 9H, CO_2_C(C*H*_3_)_3_), 1.64–1.80 (m, 1H, 7‐C*H*_2_), 1.82–1.98 (m, 2H, 6‐C*H*_2_, NCH_2_C*H*_2_CHOH), 2.02–2.19 (m, 1H, NCH_2_C*H*_2_CHOH), 2.22–2.40 (m, 1H, 8‐C*H*_2_), 3.09–3.30 (m, 2H, 5‐C*H*, 8a‐C*H*), 3.38–3.95 (m, 6H, C(=O)C*H*_2_‐aryl, NC*H*_2_CHOH, NC*H_2_*CH_2_CHOH), 4.00–4.17 (m, 1H, 4a‐C*H*), 4.25–4.42 (m, 1H, NCH_2_C*H*OH), 4.50–4.78 (m, 1H, OH), 7.09–7.18 (m, 1H, 6‐C*H*_arom_), 7.33–7.41 (m, 2H, 2‐C*H*_arom_, 5‐C*H*_arom_). Signals for 2‐C*H*_2_ and 3‐C*H*_2_ protons are not seen in the spectrum. ^13^C NMR (151 MHz, CDCl_3_): *δ* (ppm)=19.3 (1 C, C‐7), 28.5 (1 C, C‐6), 28.5 (3 C, CO_2_C(*C*H_3_)_3_), 30.4 (1 C, C‐8), 34.4 (1 C, NCH_2_ *C*H_2_CHOH), 40.0 (1 C, N*C*H_2_CHOH), 40.8 (1 C, C(=O)*C*H_2_‐aryl), 41.1 (1 C, N*C*H_2_CH_2_CHOH), 53.6 (1 C, C‐8a), 60.9 (2 C, C‐4a, C‐8), 70.0 (1 C, NCH_2_ *C*HOH), 80.9 (1 C, CO_2_ *C*(CH_3_)_3_), 128.9 (1 C, C_arom_), 130.6 (2 C, C_arom_), 131.2 (1 C, C_arom_), 131.3 (1 C, C_arom_), 132.6 (1 C, C_arom_), 155.1 (1 C, *C*O_2_C(CH_3_)_3_), 169.2 (1 C, NC(=O)benzyl). Signals for C‐2 and C‐3 carbons atoms are not seen in the spectrum. IR (neat): *ν* (cm^−1^)=3402 (OH), 2932 (C−H_aliph_), 1686 (N−C=O), 1639 (N−C=O), 1165 (N−C), 734 and 698 (1,2‐disubst. arom.). Exact Mass (APCI): *m/z* 512.2070 (calcd. 512.2077 for C_25_H_36_^35^Cl_2_N_3_O_4_ [*M*+H]^+^). HPLC (Method Pu): purity 96 % (*t*_R_=19.20 min).

##### 2‐(3,4‐Dichlorophenyl)‐1‐{(4a*R*,8*R*,8a*R*)‐ and (4a*S*,8*S*,8a*S*)‐ 8‐[(*S*)‐3‐hydroxypyrrolidin‐1‐yl]‐3,4,4 a,5,6,7,8,8 a‐octahydroquinoxalin‐1(2*H*)‐yl}ethan‐1‐one (12b)

TFA (232 μL, 3.1 mmol, 32 equiv) was added dropwise to a solution of alcohol **12a** (50 mg, 0.1 mmol, 1 equiv) in CH_2_Cl_2_ (1 mL). The mixture was stirred at room temperature overnight. After addition of toluene, the solvents were removed *in vacuo* (azeotropic distillation). Then, NaOH (0.5 M, 10 mL) was added and the mixture was extracted with CH_2_Cl_2_ (3×15 mL). After removal of the solvent *in vacuo* the residue was purified by flash column chromatography (Ø=2 cm, *h*=10 cm, CH_2_Cl_2_/CH_3_OH/NH_3_ (25 %) 89 : 10 : 1, *V*=12 mL, *R*_f_=0.28 (CH_2_Cl_2_/CH_3_OH/NH_3_ (25 %) 94 : 5 : 1)). Pale yellow oil, yield 35 mg (87 %). C_20_H_27_Cl_2_N_3_O_2_ (412.4 g/mol). ^1^H NMR (400 MHz, CDCl_3_): *δ* (ppm)=1.22–1.32 (m, 1H, 5‐C*H*_2_), 1.52–1.80 (m, 6H, 6‐C*H*_2_, 7‐C*H*_2_, NCH_2_C*H*_2_CHOH, −N*H*), 1.85–1.96 (m, 1H, 5‐C*H*_2_), 1.98–2.09 (m, 1H, NCH_2_C*H*_2_CHOH), 2.50–2.86 (m, 4H, 3‐C*H*_2_, NC*H*_2_CHOH, NC*H*_2_CH_2_CHOH), 2.90–3.07 (m, 3H, 8‐C*H*, 3‐C*H*_2_, NC*H*_2_CHOH), 3.20, 3.24 (dt, each, *J*=12.8/3.6 Hz, 1H, 2‐C*H*_2_), 3.34–3.55 (m, 2H, 2‐C*H*_2_, 4a‐C*H*), 3.59–3.83 (m, 2H, C(=O)C*H*_2_‐aryl), 4.17–4.27 (m, 1H, NCH_2_C*H*OH), 4.51, 4.56 (dd, each, *J*=11.2/3.4 Hz, 1H, 8a‐C*H*), 7.08–7.17 (m, 1H, 6‐C*H*_arom_), 7.34–7.40 (m, 2H, 2‐C*H*_arom_, 5‐C*H*_arom_). A signal for the OH proton is not seen in the spectrum. ^13^C NMR (101 MHz, CDCl_3_): *δ* (ppm)=20.0 (1 C, C‐6), 24.2 (1 C, C‐5), 32.1 (1 C, C‐7), 34.6 (1 C, NCH_2_ *C*H_2_CHOH), 40.4 (1 C, C(=O)*C*H_2_‐aryl), 42.6 (1 C, N*C*H_2_CH_2_CHOH), 46.5 (1 C, C‐2), 48.2 (1 C, N*C*H_2_CHOH), 52.7 (1 C, C‐4a), 53.9 (1 C, C‐8a), 55.6 (1 C, C‐8), 57.4 (1 C, C‐3), 71.1 (m, 1H, NCH_2_ *C*HOH), 128.5 (1 C, C_arom_), 130.6 (2 C, C_arom_), 130.8 (1 C, C_arom_), 132.6 (1 C, C_arom_), 135.7 (1 C, C_arom_), 169.8 (1 C, C(=O)‐benzyl). IR (neat): *ν* (cm^−1^)=3399 (OH), 3310 (R_2_NH), 2932 (C−H_aliph_), 1620 C(=O), 1134 (N−C), 733 and 689 (1,2‐disubst. arom.). Exact Mass (APCI): *m/z* 412.1515 (calcd. 412.1553 for C_20_H_28_^35^Cl_2_N_3_O_2_ [*M*+H]^+^). HPLC (Method Pu): purity 96 % (*t*_R_=12.85 min).

##### *tert*‐Butyl (4a*R*,5*S*,8a*S*)‐ and (4a*S*,5*R*,8a*R*)‐4‐[2‐(3,4‐dichlorophenyl)acetyl]‐5‐{(*S*)‐3‐[(methylsulfonyl)oxy]pyrrolidin‐1‐ yl}‐3,4,4 a,5,6,7,8,8 a‐octahydroquinoxaline‐1(2*H*)‐carboxylate (13)

Triethylamine (30.6 μL, 0.2 mmol, 2.3 equiv) and 4‐(dimethylamino)pyridine (12 mg, 0.1 mmol, 1 equiv) were added to a solution of alcohol **12a** (50 mg, 0.1 mmol, 1 equiv) in CH_2_Cl_2_ (5 mL). At 0 °C methanesulfonyl chloride (15.1 μL, 0.2 mmol, 2 equiv) was added dropwise. The mixture was stirred at 0 °C for 30 min and then at room temperature for 16 h. Afterwards, H_2_O (20 mL) was added, and the mixture was extracted with CH_2_Cl_2_ (3×15 mL). The combined organic layers were dried (Na_2_SO_4_), filtered and concentrated *in vacuo*. The residue was purified by flash column chromatography (Ø=2 cm, *h*=15 cm, CH_2_Cl_2_/CH_3_OH/NH_3_ (25 %) solution 98 : 1 : 1, *V*=12 mL, *R*_f_=0.72 (CH_2_Cl_2_/CH_3_OH/NH_3_ (25 %) 94 : 5 : 1)). Yellow oil, yield 47 mg (81 %). C_26_H_37_Cl_2_N_3_O_6_S (590.6 g/mol). ^1^H NMR (600 MHz, CDCl_3_): *δ* (ppm)=1.35–1.42 (m, 2H, 6‐C*H*_2_, 8‐C*H*_2_), 1.45 (s, 9H, CO_2_C(C*H*_3_)_3_), 1.58–1.78 (m, 5H, 6‐C*H*_2_, 7‐C*H*_2_, NCH_2_C*H*_2_CHOMs), 1.91–2.27 (m, 3H, 8‐C*H*_2_, NC*H*_2_CH_2_CHOMs), 2.48–2.65 (m, 1H, 2‐C*H*_2_), 2.80–3.19 (m, 4H, 2‐C*H*_2_, 5‐C*H*, NC*H*_2_CHOMs), 2.98 (s, 3H, CH_3 mesyl_), 3.45–3.55 (m, 1H, 3‐C*H*_2_), 3.61–3.76 (m, 3H, C(=O)C*H*_2_‐aryl, 8a‐C*H*), 3.77–3.86 (m, 1H, 3‐C*H*_2_), 4.03–4.16 (m, 1H, 4a‐C*H*), 5.00–5.21 (m, 1H, NCH_2_C*H*OMs), 7.04–7.18 (m, 1H, 6‐C*H*_arom_), 7.31–7.46 (m, 2H, 2‐C*H*_arom_, 5‐C*H*_arom_). ^13^C NMR (151 MHz, CDCl_3_): *δ* (ppm)=19.5 (1 C, C‐7), 23.9 (1 C, C‐6), 28.5 (3 C, CO_2_C(*C*H_3_)_3_), 30.6 (1 C, C‐8), 32.2 (1 C, N*C*H_2_CH_2_CHOMs), 38.6 (1 C, −CH_3 mesyl_), 40.2 (1 C, NCH_2_ *C*H_2_CHOMs), 40.3 (1 C, C‐3), 41.1 (1 C, C(=O)*C*H_2_‐aryl), 42.4 (1 C, C‐8a), 47.4 (1 C, C‐2), 52.1 (1 C, C‐4a), 55.6 (1 C, N*C*H_2_CHOMs), 59.1 (1 C, C‐5), 68.3 (1 C, NCH_2_ *C*HOMs), 80.6 (1 C, CO_2_ *C*(CH_3_)_3_), 128.6 (1 C, C_arom_), 130.7 (2 C, C_arom_), 131.1 (1 C, C_arom_), 132.7 (1 C, C_arom_), 135.3 (1 C, C_arom_), 167.9 (1 C, *C*O_2_C(CH_3_)_3_), 170.0 (1 C, C(=O)‐benzyl). IR (neat): *ν* (cm^−1^)=2974 (C−H_aliph_), 1686 (N−C=O), 1639 (N−C=O), 1165 (N−C), 1254 and 1034 (O=S=O), 729 and 682 (1,2‐disubst. arom.). Exact Mass (APCI): *m/z* 590.1842 (calcd. 590.1853 for C_26_H_38_^35^Cl_2_N_3_O_6_S [*M*+H]^+^). HPLC (Method Pu): purity 90 % (*t*_R_=20.76 min).

##### *tert*‐Butyl (4a*R*,5*S*,8a*S*)‐ and (4a*S*,5*R*,8a*R*)‐4‐[2‐(3,4‐dichlorophenyl)acetyl]‐5‐[(*R*)‐3‐fluoropyrrolidin‐1‐yl]‐3,4,4 a,5,6,7,8,8 a‐ octahydroquinoxaline‐1(2*H*)‐carboxylate (14a)

TBAF ⋅ 3H_2_O (63 mg, 0.2 mmol, 2.5 equiv) was added to a suspension of mesylate **13** (47 mg, 0.1 mmol, 1 equiv) in H_2_O (5 μL). The mixture was stirred at 45 °C for 16 h. Afterwards, the mixture was diluted with EtOAc (10 mL) and the mixture was washed with H_2_O and brine (3×10 mL). The organic layer was dried (Na_2_SO_4_), filtered and concentrated *in vacuo*. The residue was purified by flash column chromatography (Ø=2 cm, *h*=15 cm, CH_2_Cl_2_/CH_3_OH/NH_3_ (25 %) 98 : 1 : 1→94 : 5 : 1, *V*=12 mL, *R*_f_=0.58 (CH_2_Cl_2_/CH_3_OH/NH_3_ (25 %) 94 : 5 : 1)). Yellowish oil, yield 20 mg (50 %). C_25_H_34_Cl_2_FN_3_O_3_ (514.5 g/mol). ^1^H NMR (400 MHz, CDCl_3_): *δ* (ppm)=1.33–1.49 (m, 5H, 6‐C*H*_2_, 7‐C*H*_2_, NCH_2_C*H*_2_CHF), 1.45 (s, 9H, CO_2_C(C*H*_3_)_3_), 1.51–1.60 (m, 1H, 8‐C*H*_2_), 1.66–1.73 (m, 1H, 6‐C*H*_2_), 1.89–2.07 (m, 2H, NC*H*_2_CH_2_CHF), 2.07–2.19 (m, 1H, 8‐C*H*_2_), 2.71–3.10 (m, 4H, 2‐C*H*_2_, 5‐C*H*, NC*H*_2_CHF), 3.30–3.44 (m, 1H, NC*H*_2_CHF), 3.44–3.56 (m, 1H, 3‐C*H*_2_), 3.59–3.77 (m, 3H, C(=O)C*H*_2_‐aryl, 8a‐C*H*), 3.79–3.92 (m, 1H, 3‐C*H*_2_), 4.05–4.17 (m, 1H, 4a‐C*H*), 4.95–5.22 (m, 1H, NCH_2_C*H*F), 7.10 (td, *J*=8.0, 2.2 Hz, 1H, 6‐C*H*_arom_), 7.28–7.44 (m, 2H, 2‐C*H*_arom_, 5‐C*H*_arom_). ^13^C NMR (101 MHz, CDCl_3_): *δ* (ppm)=19.7 (1 C, C‐7), 28.5 (3 C, CO_2_C(*C*H_3_)_3_), 29.8 (1 C, C‐6), 30.7 (1 C, C‐8), 32.7 (d, *J*=17.9 Hz 1 C, N*C*H_2_CH_2_CHF), 33.0 (d, *J*=18.7 Hz, 1 C, NCH_2_ *C*H_2_CHF), 40.9 (1 C, C‐3), 41.8 (1 C, C(=O)*C*H_2_‐aryl), 47.6 (1 C, C‐2), 52.5 (1 C, C‐4a), 56.5 (d, *J*=23.5 Hz, 1 C, N*C*H_2_CHF), 59.3 (1 C, C‐8a), 59.9 (1 C, C‐5), 80.5 (1 C, CO_2_ *C*(CH_3_)_3_), 93.4 (d, *J*=174.8 Hz, 1 C, NCH_2_ *C*HF), 128.5 (1 C, C_arom_), 130.6 (2 C, C_arom_), 131.0 (1 C, C_arom_), 132.6 (1 C, C_arom_), 135.4 (1 C, C_arom_), 155.2 (1 C, *C*O_2_C(CH_3_)_3_), 170.0 (1 C, NC(=O)‐benzyl). IR (neat): *ν* (cm^−1^)=2932 (C−H_aliph_), 1686 (N−C=O), 1639 (N−C=O), 1157 (N−C), 729 and 683 (1,2‐disubst. arom.). Exact Mass (APCI): *m/z* 514.2061 (calcd. 514.2034 for C_25_H_35_^35^Cl_2_FN_3_O_3_ [*M*+H]^+^). HPLC (Method Pu): purity 74 % (*t*_R_=20.70 min).

##### 2‐(3,4‐Dichlorophenyl)‐1‐{(4a*R*,8*R*,8a*R*)‐ and (4a*S*,8*S*,8a*S*)‐8‐ [(*R*)‐3‐fluoropyrrolidin‐1‐yl]‐3,4,4 a,5,6,7,8,8 a‐octahydroquinoxalin‐1(2*H*)‐yl}ethan‐1‐one (14b)

TFA (739 μL, 10 mmol, 32 equiv) was added dropwise to a solution of Boc‐derivative **14a** (160 mg, 0.3 mmol, 1 equiv) in CH_2_Cl_2_ (3 mL). The mixture was stirred at room temperature overnight. After addition of toluene, the solvents were removed *in vacuo* (azeotropic distillation). Then, NaOH (0.5 M, 10 mL) was added and the mixture was extracted with CH_2_Cl_2_ (3×15 mL). After removal of the solvent *in vacuo*, the residue was purified by flash column chromatography (Ø=3 cm, *h*=10 cm, CH_2_Cl_2_/CH_3_OH/NH_3_ (25 %) 94 : 5 : 1, *V*=30 mL, *R*_f_=0.46 (CH_2_Cl_2_/CH_3_OH/NH_3_ (25 %) 94 : 5 : 1)). Colorless oil, yield 96 mg (77 %). C_20_H_26_Cl_2_FN_3_O (414.3 g/mol). ^1^H NMR (400 MHz, CDCl_3_): *δ* (ppm)=1.22–1.37 (m, 1H, 5‐C*H*_2_), 1.43–1.69 (m, 6H, 6‐C*H*_2_, 7‐C*H*_2_, NCH_2_C*H*_2_CHF), 1.81–2.09 (m, 3H, NC*H*_2_CH_2_CHF, 5‐C*H*_2_), 2.64–2.86 (m, 2H, NC*H*_2_CHF), 2.94–3.07 (m, 3H, 3‐C*H*_2_, 8‐C*H*), 3.10–3.22 (m, 1H, C(=O)C*H*_2_‐aryl), 3.30–3.53 (m, 2H, 4a‐C*H*, C(=O)C*H*_2_‐aryl), 3.60–3.84 (m, 2H, 2‐C*H*_2_), 4.36–4.64 (m, 1H, 8a‐C*H*), 4.93–5.21 (m, 1H, NCH_2_C*H*F), 7.01–7.19 (m, 1H, 6‐C*H*_arom_), 7.28–7.44 (m, 2H, 2‐C*H*_arom_, 5‐C*H*_arom_). A signal for the NH proton is not seen in the spectrum. ^13^C NMR (101 MHz, CDCl_3_): *δ* (ppm)=20.2 (1 C, C‐6), 23.4 (1 C, C‐5), 32.0 (1 C, C‐7), 32.8 (d, *J*=22.5 Hz,1 C, NCH_2_ *C*H_2_CHF), 33.2 (d, *J*=21.6 Hz, 1 C, N*C*H_2_CH_2_CHF), 40.6 (1 C, C‐2), 42.4 (1 C, C(=O)*C*H_2_‐aryl), 47.1 (1 C, C‐3), 52.3 (1 C, C‐4a), 54.0 (1 C, C‐8a), 54.8 (d, *J*=22.7 Hz, 1 C, N*C*H_2_CHF), 55.6 (1 C, C‐8), 93.7 (d, *J*=176.1 Hz, 1 C, NCH_2_ *C*HF), 128.3 (1 C, C_arom_), 130.5 (2 C, C_arom_), 131.4 (1 C, C_arom_), 132.5 (1 C, C_arom_), 135.9 (1 C, C_arom_), 170.1 (1 C, C(=O)benzyl). IR (neat): *ν* (cm^−1^)=3313 (R_2_NH), 2932 (C−H_aliph_), 1628 (N−C=O), 1134 (N−C), 733 and 679 (1,2‐disubst. arom.). Exact Mass (APCI): *m/z* 414.1536 (calcd. 414.1510 for C_20_H_27_^35^Cl_2_FN_3_O [*M*+H]^+^). HPLC (Method Pu): purity 95 % (*t*_R_=13.89 min).

##### Methyl (4a*R*,5*S*,8a*S*)‐ and (4a*S*,5*R*,8a*R*)‐4‐[2‐(3,4‐dichlorophenyl)acetyl]‐5‐[(*R*)‐3‐fluoropyrrolidin‐1‐yl]‐3,4,4 a,5,6,7,8,8 a‐ octahydroquinoxaline‐1(2*H*)‐carboxylate (14c)

Under a N_2_ atmosphere, pyridine (49 μL, 0.60 mmol, 5 equiv) was added to a solution of secondary amine **14b** (50 mg, 0.12 mmol, 1 equiv) in dry CH_2_Cl_2_ (0.5 mL). At 0 °C, a solution of methyl chloroformate (56 μL, 0.72 mmol, 6 equiv) in CH_2_Cl_2_ (0.5 mL) was added dropwise. The ice bath was removed and the mixture was stirred at room temperature for 1 h. H_2_O (10 mL) was added and the mixture was extracted with CH_2_Cl_2_ (3×10 mL). The combined organic layers were dried (Na_2_SO_4_), filtered and concentrated *in vacuo*. The residue was purified by flash column chromatography (Ø=2 cm, *h*=14 cm, CH_2_Cl_2_/CH_3_OH/NH_3_ (25 %) 97 : 2 : 1, *V*=12 mL, *R*_f_=0.8 (CH_2_Cl_2_/CH_3_OH/NH_3_ (25 %) 94 : 5 : 1). Pale yellow oil, yield 10 mg (18 %). C_22_H_28_Cl_2_FN_3_O_3_ (472.4 g/mol). ^1^H NMR (400 MHz, CDCl_3_): *δ* (ppm)=1.32–1.50 (m, 2H, 7‐C*H*_2_), 1.52–1.63 (m, 1H, 8‐C*H*_2_), 1.65–1.81 (m, 2H, 6‐C*H*_2_), 1.84–2.00 (m, 2H, NCH_2_C*H*_2_CHF), 2.06–2.18 (m, 1H, 8‐C*H*_2_), 2.27–2.58 (m, 1H, NC*H*_2_CH_2_CHF), 2.69–3.09 (m, 4H, 8‐C*H*, NC*H*_2_CH_2_CHF, NC*H*_2_CHF), 3.32–3.49 (m, 1H, 3‐C*H*_2_), 3.49–3.63 (m, 2H, 2‐C*H*_2_, 8a‐C*H*), 3.64–3.84 (m, 4H, C(=O)C*H*_2_‐aryl, 2‐C*H*_2_, 3‐C*H*_2_), 3.69 (s, 3H, CO_2_C*H*_3_), 4.13–4.23 (m, 1H, 4a‐C*H*), 4.97–5.22 (m, 1H, NCH_2_C*H*F), 7.09 (td, *J*=8.0, 2.1 Hz, 1H, 6‐C*H*_arom_), 7.28–7.43 (m, 2H, 2‐C*H*_arom_, 5‐C*H*_arom_). ^13^C NMR (101 MHz, CDCl_3_): *δ* (ppm)=19.4 (1 C, C‐7), 19.5 (1 C, C‐6), 30.5 (1 C, C‐8), 32.6 (d, *J*=16.5 Hz, 1 C, NCH_2_ *C*H_2_CHF), 32.8 (d, *J*=17.0 Hz, 1 C, N*C*H_2_CH_2_CHF), 40.2 (1 C, C‐3), 41.6 (1 C, C(=O)*C*H_2_‐aryl), 41.9 (1 C, C‐2), 47.7 (1 C, N*C*H_2_CH_2_CHF), 52.5 (2 C, C‐4a, C‐8a), 52.9 (1 C, CO_2_ *C*H_3_), 56.7 (d, *J*=21.9 Hz, 1 C, N*C*H_2_CHOF), 59.5 (1 C, C‐5), 93.4 (d, *J*=175.1 Hz, 1 C, NCH_2_ *C*HF), 128.6 (1 C, C_arom_), 130.6 (2 C, C_arom_), 131.0 (1 C, C_arom_), 132.6 (1 C, C_arom_), 135.2 (1 C, C_arom_), 156.4 (1 C, *C*O_2_CH_3_)., 171.2 (1 C, C(=O)‐benzyl). IR (neat): *ν* (cm^−1^)=2947 (C−H_aliph_), 1694 (N−C=O), 1639 (N−C=O), 1126 (N−C), 733 and 683 (1,2‐disubst. arom.). Exact Mass (APCI): *m/z* 472.1548 (calcd. 472.1565 for C_22_H_29_^35^Cl_2_FN_3_O_3_ [*M*+H]^+^). HPLC (Method Pu): purity 96 % (*t*_R_=17.87 min).

##### *tert*‐Butyl (4a*R*,5*S*,8a*S*)‐ and (4a*S*,5*R*,8a*R*)‐5‐{(*S*)‐3‐[(*tert*‐butyldiphenylsilyl)oxy]pyrrolidin‐1‐yl}‐4‐[2‐(3,4‐dichlorophenyl)‐ acetyl]‐3,4,4 a,5,6,7,8,8 a‐octahydroquinoxaline‐1(2*H*)‐carboxylate (15)

Imidazole (167 mg, 2.45 mmol, 2.5 equiv), DMAP (6 mg, 0.05 mmol, 0.05 equiv) and TBDPSCl (280 μL, 1.08 mmol, 1.1 equiv) were added successively to a solution of **12a** (500 mg, 0.98 mmol, 1 equiv) in CH_2_Cl_2_ (8 mL). The mixture was stirred at room temperature overnight. After addition of CH_2_Cl_2_ (10 mL), the mixture was washed with brine (3×10 mL). The organic layer was dried (Na_2_SO_4_), filtered and concentrated *in vacuo*. The residue was purified by flash column chromatography (Ø=3 cm, *h*=14 cm, cHex/EtOAc 7 : 3, *V*=30 mL, *R*_f_=0.48 (EtOAc/cHex 5 : 5)). Pale yellow oil, yield 534 mg (74 %). C_41_H_53_Cl_2_N_3_O_4_Si (750.9 g/mol). ^1^H NMR (600 MHz, CDCl_3_): *δ* (ppm)=0.99–1.10 (s, 9H, SiC(C*H*_3_)_3_), 1.30–1.39 (m, 2H, 6‐C*H*_2_, 7‐C*H*_2_), 1.41–1.56 (m, 2H, 7‐C*H*_2_, 8‐C*H*_2_), 1.44 (s, 9H, CO_2_C(C*H*_3_)_3_), 1.68–1.87 (m, 3H, 6‐C*H_2_*, NCH_2_C*H*_2_CHOSi), 2.06–2.15 (m, 1H, 8‐C*H*_2_), 2.52–2.62 (m, 1H, NC*H_2_*CH_2_CHOSi), 2.66–2.72 (m, 1H, NC*H*_2_CHOSi), 2.73–2.83 (m, 1H, NC*H*_2_CHOSi), 2.84–2.93 (m, 2H, 5‐C*H*, NC*H_2_*CH_2_CHOSi), 3.23–3.39 (m, 1H, 3‐C*H*_2_), 3.41–3.52 (m, 1H, 2‐C*H*_2_), 3.54–3.76 (m, 3H, C(=O)C*H*_2_‐aryl, 2‐C*H*_2_), 3.80–3.90 (m, 1H, 3‐C*H*_2_), 3.98–4.08 (m, 1H, 8a‐C*H*), 4.24–4.37 (m, 1H, NCH_2_C*H*OSi), 6.98–7.07 (m, 1H, 6‐C*H*_arom_), 7.22–7.30 (m, 2H, 2‐C*H*_arom_, 5‐C*H*_arom_), 7.32–7.46 (m, 6H, C*H*_arom_), 7.60–7.67 (m, 4H, C*H*_arom_). A signal for 4a‐C*H* proton is not seen in the spectrum. ^13^C NMR (151 MHz, CDCl_3_): *δ* (ppm)=19.2 (1 C, C‐7), 19.7 (1 C, C‐6), 21.2 (1 C, Si*C*(CH_3_)_3_), 27.0 (3 C, SiC(*C*H_3_)_3_), 28.5 (3 C, CO_2_C(*C*H_3_)_3_), 30.7 (1 C, C‐8), 35.2 (1 C, NCH_2_ *C*H_2_CHOSi), 38.9 (1 C, C(=O)*C*H_2_‐aryl), 40.7 (1 C, C‐3), 42.1 (1 C, C‐2), 52.6 (1 C, C‐8a), 56.9 (1 C, N*C*H_2_CH_2_CHOSi), 59.8 (1 C, C‐5), 60.5 (1 C, N*C*H_2_CHOSi), 72.5 (1 C, NCH_2_ *C*HOSi), 80.4 (1 C, CO_2_ *C*(CH_3_)_3_), 127.7 (4 C, C_arom_), 128.5 (2 C, C_arom_), 128.7 (1 C, C_arom_), 129.7 (2 C, C_arom_), 130.5 (2 C, C_arom_), 130.7 (1 C, C_arom_), 131.0 (1 C, C_arom_), 132.6 (1 C, C_arom_), 135.8 (4 C, C_arom_), 155.3 (1 C, *C*O_2_C(CH_3_)_3_), 169.8 (1 C, NC(=O)‐benzyl). A signal for C‐4a carbon atom is not seen in the spectrum. IR (neat): *ν* (cm^−1^)=2932 (C−H_aliph_), 1690 (N−C=O), 1643 (N−C=O), 1107 (N−C), 729 (1,2‐disubst. arom.), 702 (SiC_6_H_5_). Exact Mass (APCI): *m/z* 394.1437 (calcd. 394.1447 for C_20_H_26_^35^Cl_2_N_3_O [*M*−C_21_H_28_O_3_Si+H]^+^). HPLC (Method Pu): purity 92 % (*t*_R_=28.82 min).

##### 1‐((4a*R*,8*R*,8a*R*)‐ and (4a*S*,8*S*,8a*S*)‐8‐{(*S*)‐3‐[(*tert*‐Butyldiphenylsilyl)oxy]pyrrolidin‐1‐yl}‐3,4,4 a,5,6,7,8,8 a‐octahydroquinoxalin‐1(2*H*)‐yl)‐2‐(3,4‐dichlorophenyl)ethan‐1‐one (16)

TFA (1.68 mL, 22.6 mmol, 32 equiv) was added dropwise to a solution of **15** (520 mg, 0.7 mmol, 1 equiv) in CH_2_Cl_2_ (10 mL). The mixture was stirred at room temperature overnight. After addition of toluene, the solvents were removed *in vacuo* (azeotropic distillation). Then, NaOH (0.5 M, 20 mL) was added and the mixture was extracted with CH_2_Cl_2_ (3×20 mL). After removal of the solvent *in vacuo*, the residue was purified by flash column chromatography (Ø=3 cm, *h*=10 cm, CH_2_Cl_2_/CH_3_OH/NH_3_ (25 %) 94 : 5 : 1, *V*=30 mL, *R*_f_=0.60 (CH_2_Cl_2_/CH_3_OH/NH_3_ (25 %) 94 : 5 : 1)). Yellow oil, yield 410 mg (89 %). C_36_H_45_Cl_2_N_3_O_2_Si (650.8 g/mol). ^1^H NMR (600 MHz, CDCl_3_): *δ* (ppm)=0.98–1.11 (m, 9H, SiC(C*H*_3_)_3_), 1.21–1.40 (m, 2H, 5‐C*H*_2_, 7‐C*H*_2_), 1.54–1.76 (m, 5H, 6‐C*H*_2_, 7‐C*H*_2_, NCH_2_C*H*_2_CHOSi, N*H*), 1.84–2.09 (m, 2H, 5‐C*H*_2_, NCH_2_C*H*_2_CHOSi), 2.42–2.56 (m, 1H, NC*H_2_*CH_2_CHOSi), 2.59–2.88 (m, 4H, 3‐C*H*_2_, NC*H*_2_CHOSi), 2.90–3.08 (m, 2H, NC*H_2_*CH_2_CHOSi, 8‐C*H*), 3.20–3.38 (m, 2H, 2‐C*H*_2_, 4a‐C*H*), 3.41–3.49 (m, 1H, 2‐C*H*_2_), 3.57–3.79 (m, 2H, C(=O)C*H*_2_‐aryl), 4.20–4.41 (m, 1H, NCH_2_C*H*OSi), 4.46–4.58 (m, 1H, 8a‐C*H*), 7.01–7.25 (m, 2H, 2‐C*H*_arom_, 6‐C*H*_arom_), 7.28–7.32 (m, 1H, 5‐C*H*_arom_), 7.33–7.45 (m, 6H, C*H*_arom_), 7.59–7.69 (m, 4H, C*H*_arom_). ^13^C NMR (151 MHz, CDCl_3_): *δ* (ppm)=19.2 (1 C, C‐6), 20.4 (1 C, Si*C*(CH_3_)_3_), 23.2 (1 C, C‐5), 26.7 (1 C, C‐7), 27.0 (3 C, SiC(*C*H_3_)_3_), 32.1 (1 C, NCH_2_ *C*H_2_CHOSi), 40.5 (1 C, C(=O)*C*H_2_‐aryl), 42.4 (1 C, C‐2), 44.3 (1 C, N*C*H_2_CHOSi), 47.2 (1 C, N*C*H_2_CH_2_CHOSi), 52.5 (1 C, C‐4a), 53.6 (1 C, C‐8a), 56.0 (1 C, C‐8), 59.4 (1 C, C‐3), 72.6 (1 C, NCH_2_ *C*HOSi), 127.7 (4 C, C_arom_), 128.1 (2 C, C_arom_), 128.7 (1 C, C_arom_), 129.6 (2 C, C_arom_), 130.4 (2 C, C_arom_), 130.6 (1 C, C_arom_), 131.2 (1 C, C_arom_), 132.4 (1 C, C_arom_), 135.8 (4 C, C_arom_), 169.7 (1 C, NC(=O)‐benzyl). IR (neat): *ν* (cm^−1^)=2932 (C−H_aliph_), 1632 C(=O), 1103 (N−C), 737 (1,2‐disubst. arom.), 702 (SiC_6_H_5_). Exact Mass (APCI): *m/z* 650.2755 (calcd. 650.2731 for C_36_H_46_^35^Cl_2_N_3_O_2_Si [*M*+H]^+^). HPLC (Method Pu): purity 86 % (*t*_R_=22.55 min).

##### Methyl (4a*R*,5*S*,8a*S*)‐ and (4a*S*,5*R*,8a*R*)‐5‐{(*S*)‐3‐[(*tert*‐butyldiphenylsilyl)oxy] pyrrolidin‐1‐yl}‐4‐[2‐(3,4‐dichlorophenyl)acetyl]‐3,4,4 a,5,6,7,8,8 a‐octahydroquinoxaline‐1(2*H*)‐carboxylate (17)

Under a N_2_ atmosphere, pyridine (32 μL, 0.40 mmol, 5 equiv) was added to a solution of **16** (50 mg, 0.08 mmol, 1 equiv) in dry CH_2_Cl_2_ (0.2 mL). At 0 °C, a solution of methyl chloroformate (37 μL, 0.48 mmol, 6 equiv) in CH_2_Cl_2_ (0.2 mL) was added dropwise. The ice bath was removed and the mixture was stirred at room temperature for 1 h. H_2_O (10 mL) was added and the mixture was extracted with CH_2_Cl_2_ (3×10 mL). The combined organic layers were dried (Na_2_SO_4_), filtered and concentrated *in vacuo*. The residue was purified by flash column chromatography (Ø=2 cm, *h*=10 cm, CH_2_Cl_2_/CH_3_OH/NH_3_ (25 %) 97 : 2 : 1, *V*=12 mL, *R*_f_=0.84 (CH_2_Cl_2_/CH_3_OH/NH_3_ (25 %) 94 : 5 : 1)). Pale yellow oil, yield 44 mg (78 %). C_38_H_47_Cl_2_N_3_O_4_Si (708.8 g/mol). ^1^H NMR (400 MHz, CDCl_3_): *δ* (ppm)=1.01–1.10 (m, 9H, SiC(C*H*_3_)_3_), 1.18–1.47 (m, 3H, 6‐C*H*_2_, 7‐C*H*_2_, 8‐C*H*_2_), 1.65–1.88 (m, 4H, NCH_2_C*H*_2_CHOSi, 6‐C*H*_2_, 7‐C*H*_2_), 2.05–2.17 (m, 1H, 8‐C*H*_2_), 2.52–2.63 (m, 1H, NC*H_2_*CH_2_CHOSi), 2.66–2.73 (m, 1H, NC*H*_2_CHOSi), 2.75–2.82 (m, 1H, NC*H*_2_CHOSi), 2.84–2.99 (m, 2H, 5‐C*H*, NC*H_2_*CH_2_CHOSi), 3.25–3.43 (m, 1H, 3‐C*H*_2_), 3.45–3.89 (m, 4H, 3‐C*H*_2_, 8a‐C*H*, C(=O)C*H*_2_‐aryl), 3.69 (s, 3H, CO_2_C*H*_3_), 4.03–4.15 (m, 1H, 4a‐C*H*), 4.25–4.39 (m, 1H, NCH_2_C*H*OSi), 7.02 (d, *J*=8.0 Hz, 1H, 6‐C*H*_arom_), 7.27–7.31 (m, 2H, 2‐C*H*_arom_, 5‐C*H*_arom_), 7.32–7.47 (m, 6H, C*H*_arom_), 7.58–7.73 (m, 4H, C*H*_arom_). Signals for 2‐C*H*_2_ protons are not seen in the spectrum. ^13^C NMR (101 MHz, CDCl_3_): *δ* (ppm)=19.2 (1 C, C‐7), 19.6 (1 C, Si*C*(CH_3_)_3_), 26.7 (1 C, C‐6), 27.1 (3 C, SiC(*C*H_3_)_3_), 30.5 (1 C, C‐8), 35.2 (1 C, NCH_2_ *C*H_2_CHOSi), 40.0 (1 C, C(=O)*C*H_2_‐aryl), 42.2 (1 C, 3‐C*H*_2_), 49.0 (1 C, N*C*H_2_CH_2_CHOSi), 52.6 (1 C, C‐4a), 52.9 (1 C, CO_2_ *C*H_3_), 59.4 (1 C, C‐5), 59.8 (1 C, N*C*H_2_CHOSi), 72.5 (1 C, NCH_2_ *C*HOSi), 127.7 (4 C, C_arom_), 127.9 (2 C, C_arom_), 128.4 (1 C, C_arom_), 129.7 (2 C, C_arom_), 130.6 (2 C, C_arom_), 131.0 (1 C, C_arom_), 132.3 (1 C, C_arom_), 134.9 (1 C, C_arom_), 135.8 (4 C, C_arom_), 156.4 (1 C, *C*O_2_CH_3_), 169.8 (1 C, C(=O)‐benzyl). Signals for C‐2 and C‐8a carbon atoms are not seen in the spectrum. IR (neat): *ν* (cm^−1^)=2932 (C−H_aliph_), 1697 (N−C=O), 1643 (N−C=O), 1107 (N−C), 737 (1,2‐disubst. arom.), 702 (SiC_6_H_5_). Exact Mass (APCI): *m/z* 650.2751 (calcd. 650.2731 for C_36_H_46_^35^Cl_2_N_3_O_2_Si [*M*−C_2_H_2_O_2_+H]^+^). HPLC (Method Pu): purity 85 % (*t*_R_=26.57 min).

##### Methyl (4a*R*,5*S*,8a*S*)‐ and (4a*S*,5*R*,8a*R*)‐4‐[2‐(3,4‐dichlorophenyl)acetyl]‐5‐[(*S*)‐3‐hydroxypyrrolidin‐1‐yl]‐3,4,4 a,5,6,7, 8,8 a‐octahydroquinoxaline‐1(2*H*)‐carboxylate (12c)

TBAF ⋅ 3H_2_O (178 mg, 0.56 mmol, 1.1 equiv) was added to a solution of **17** (364 mg, 0.51 mmol, 1 equiv) in THF (5 mL). The mixture was stirred at room temperature overnight. H_2_O (10 mL) was added and the mixture was extracted with CH_2_Cl_2_ (3×10 mL). The combined organic layers were dried (Na_2_SO_4_), filtered and the solvent was removed *in vacuo*. The residue was purified by flash column chromatography (Ø=2 cm, *h*=13 cm, CH_2_Cl_2_/CH_3_OH 9 : 1, *V*=12 cm, *R*_f_=0.42 (CH_2_Cl_2_/CH_3_OH/NH_3_ (25 %) 94 : 5 : 1) Colorless oil, yield 154 mg (64 %). C_22_H_29_Cl_2_N_3_O_4_ (470.4 g/mol). ^1^H NMR (600 MHz, CDCl_3_): *δ* (ppm)=1.31–1.44 (m, 2H, 6‐C*H*_2_, 7‐C*H*_2_), 1.47–1.59 (m, 1H, 8‐C*H*_2_), 1.65–1.75 (m, 2H, 7‐C*H*_2_, NCH_2_C*H*_2_CHOH), 1.76–1.86 (m, 1H, 6‐C*H*_2_), 1.98–2.08 (m, 1H, NCH_2_C*H*_2_CHOH), 2.11–2.24 (m, 1H, 8‐C*H*_2_), 2.39–2.60 (m, 1H, NC*H*_2_CHOH), 2.61–2.75 (m, 1H, NC*H_2_*CH_2_CHOH), 2.76–2.98 (m, 2H, NC*H_2_*CH_2_CHOH, NC*H*_2_CHOH), 3.01–3.16 (m, 1H, 5‐C*H*), 3.51–3.61 (m, 1H, 3‐C*H*_2_), 3.63–3.80 (m, 3H, 8a‐C*H*, C(=O)C*H*_2_‐aryl), 3.69 (s, 3H, CO_2_C*H*_3_), 3.88–4.01 (m, 1H, 3‐C*H*_2_), 4.12–4.34 (m, 2H, NCH_2_C*H*OH, 4a‐C*H*), 4.56 (brs, 1H, OH), 7.07–7.17 (m, 1H, 6‐C*H*_arom_), 7.32–7.41 (m, 2H, 2‐C*H*_arom_, 5‐C*H*_arom_). Signals for 2‐C*H*_2_ protons are not seen in the spectrum. ^13^C NMR (151 MHz, CDCl_3_): *δ* (ppm)=19.4 (1 C, C‐7), 24.2 (1 C, C‐6), 30.5 (1 C, C‐8), 34.7 (1 C, NCH_2_ *C*H_2_CHOH), 40.4 (1 C, C(=O)*C*H_2_‐aryl), 41.1 (1 C, C‐3), 42.8 (1 C, C‐8a), 49.0 (1 C, N*C*H_2_CH_2_CHOH), 52.6 (1 C, C‐4a), 53.0 (1 C, CO_2_ *C*H_3_), 56.3 (1 C, N*C*H_2_CHOH), 60.0 (1 C, C‐5), 71.0 (1 C, NCH_2_ *C*HOH), 128.7 (1 C, C_arom_), 130.7 (2 C, C_arom_), 131.0 (1 C, C_arom_), 132.7 (1 C, C_arom_), 135.0 (1 C, C_arom_), 156.4 (1 C, *C*O_2_CH_3_), 170.9 (1 C, C(=O)‐benzyl). A signal for C‐2 carbon atom is not seen in the spectrum. IR (neat): *ν* (cm^−1^)=3402 (OH), 2940 (C−H_aliph_), 1690 (N−C=O), 1632 (N−C=O), 1107 (N−C), 772 and 683 (1,2‐disubst. arom.). Exact Mass (APCI): *m/z* 470.1565 (calcd. 470.1608 for C_22_H_30_^35^Cl_2_N_3_O_4_ [*M*+H]^+^). HPLC (Method Pu): purity 99 % (*t*_R_=16.55 min).

##### Methyl (4a*R*,5*S*,8a*S*)‐ and (4a*S*,5*R*,8a*R*)‐4‐[2‐(3,4‐dichlorophenyl)acetyl]‐5‐[(*S*)‐3‐(methylsulfonyloxy)pyrrolidin‐1‐yl]‐ 3,4,4 a,5,6,7,8,8 a‐octahydroquinoxaline‐1(2*H*)‐carboxylate (19)

Triethylamine (66 μL, 0.5 mmol, 2.3 equiv) and 4‐(dimethylamino)pyridine (26 mg, 0.2 mmol, 1 equiv) were added to a solution of **12c** (100 mg, 0.2 mmol, 1 equiv) in CH_2_Cl_2_ (10 mL). At 0 °C, methanesulfonyl chloride (33 μL, 0.4 mmol, 2 equiv) was added dropwise. The mixture was stirred at 0 °C for 30 min and then at room temperature for 16 h. After addition of H_2_O (20 mL), the mixture was extracted with CH_2_Cl_2_ (3×20 mL). The combined organic layers were dried (Na_2_SO_4_), filtered and concentrated *in vacuo*. The residue was purified by flash column chromatography (Ø=2 cm, *h*=15 cm, CH_2_Cl_2_/CH_3_OH 98 : 2, *V*=12 mL, *R*_f_=0.64 (CH_2_Cl_2_/CH_3_OH/NH_3_ (25 %) 89 : 10 : 1)). Yellow oil, yield 63 mg (55 %). C_23_H_31_Cl_2_N_3_O_4_S (548.5 g/mol). ^1^H NMR (600 MHz, CDCl_3_): *δ* (ppm)=1.35–1.50 (m, 2H, 6‐C*H*_2_, 7‐C*H*_2_), 1.65–1.78 (m, 2H, 6‐C*H*_2_, 7‐C*H*_2_), 1.94–2.01 (m, 1H, NCH_2_C*H*_2_CHOMs), 2.02–2.24 (m, 3H, 8‐C*H*_2_, NCH_2_C*H*_2_CHOMs), 2.49–2.60 (m, 1H, NC*H*_2_CH_2_CHOMs), 2.88–2.92 (m, 1H, NC*H*_2_CHOMs), 2.94–3.03 (m, 5H, −C*H*_3 mesyl_, 4a‐C*H*, NC*H*_2_CH_2_CHOMs), 3.07–3.16 (m, 1H, NC*H*_2_CHOMs), 3.38–3.50 (m, 1H, 2‐C*H*_2_), 3.52–3.60 (m, 1H, 3‐C*H*_2_), 3.61–3.82 (m, 5H, C(=O)C*H*_2_‐aryl, 2‐C*H*_2_, 3‐C*H*_2_, 8a‐C*H*), 3.69 (s, 3H, CO_2_C*H*_3_), 4.09–4.22 (m, 1H, 4a‐C*H*), 4.98–5.18 (m, 1H, NCH_2_C*H*OMs), 7.03–7.15 (m, 1H, 6‐C*H*_arom_), 7.31–7.36 (m, 1H, 2‐C*H*_arom_), 7.39 (m, 1H, 5‐C*H*_arom_). ^13^C NMR (151 MHz, CDCl_3_): *δ* (ppm)=19.4 (1 C, C‐7), 24.1 (1 C, C‐6), 29.8 (1 C, C‐8), 32.2 (1 C, NCH_2_ *C*H_2_CHOMs), 38.6 (1 C, *C*H_3 mesyl_), 40.2 (1 C, C(=O)*C*H_2_‐aryl), 40.4 (1 C, C‐8a), 41.7 (1 C, C‐3), 42.1 (1 C, C‐2), 47.6 (1 C, N*C*H_2_CH_2_CHOMs), 52.1 (1 C, C‐4a), 52.9 (1 C, CO_2_ *C*H_3_), 55.7 (1 C, N*C*H_2_CHOMs), 59.2 (1 C, C‐5), 80.7 (1 C, NCH_2_ *C*HOMs), 128.5 (1 C, C_arom_), 130.7 (2 C, C_arom_), 131.0 (1 C, C_arom_), 132.7 (1 C, C_arom_), 135.2 (1 C, C_arom_), 156.4 (1 C, *C*O_2_CH_3_), 170.0 (1 C, C(=O)‐benzyl). IR (neat): *ν* (cm^−1^)=2936 (C−H_aliph_), 1694 (N−C=O), 1639 (N−C=O), 1130 (N−C), 1261 and 1030 (O=S=O), 772 and 683 (1,2‐disubst. arom.). Exact Mass (APCI): *m/z* 548.1421 (calcd. 548.1383 for C_23_H_32_^35^Cl_2_N_3_O_4_S [*M*+H]^+^). HPLC (Method Pu): purity 99 % (*t*_R_=18.02 min).

### Receptor binding studies

#### Preparation of membrane homogenates from guinea pig brain^41^, ^42^, ^43^

Five guinea pig brains were homogenized with the potter (500‐800 rpm, 10 up‐and‐down strokes) in 6 volumes of cold 0.32 M sucrose. The suspension was centrifuged at 1200×*g* for 10 min at 4 °C. The supernatant was separated and centrifuged at 23 500×*g* for 20 min at 4 °C. The pellet was resuspended in 5–6 volumes of buffer (50 mM Tris, pH 7.4) and centrifuged again at 23500×*g* (20 min, 4 °C). This procedure was repeated twice. The final pellet was resuspended in 5–6 volumes of buffer and frozen (−80 °C) in 1.5 mL portions containing about 1.5 mg protein/mL.

#### Determination of KOR affinity (guinea pig brain)^35^, ^36^, ^37^, ^38^

The assay was performed with the radioligand [^3^H]U‐69,593 (55 Ci/mmol, Amersham, Little Chalfont, UK). The thawed guinea pig brain membrane preparation (about 100 μg of the protein) was incubated with various concentrations of test compounds, 1 nM [^3^H]U‐69,593, and TRIS‐MgCl_2_‐Puffer (50 mM, 8 mM MgCl_2_, pH 7.4) at 37 °C. The non‐specific binding was determined with 10 μM unlabeled U‐69,593. The K_d_‐value of U‐69,593 is 0.69 nM.

#### Affinity towards MOR, DOR,^35^‐^38^ σ_1_ and σ_2_ recetors^41^, ^42^, ^43^

Materials, preparation of membrane homogenates from various tissues, protein determination, and the general procedure for binding assays are detailed in the Supporting Information.

### *In vitro* functional assays

#### cAMP inhibition assay

To measure KOR G_αi_‐mediated cAMP inhibition, HEK 293T (ATCC CRL‐11268) cells were co‐transfected with human KOR along with a luciferase‐based cAMP biosensor (GloSensor; Promega) and assays were performed similar to previously described.^55^ After 16 h, transfected cells were plated into Poly‐lysine coated 384‐well white clear bottom cell culture plates with DMEM+1 % dialyzed FBS at a density of 15 000–20 000 cells per 40 μL per well and incubated at 37 °C with 5 % CO_2_ overnight. The next day, drug solutions were prepared in fresh drug buffer (20 mM HEPES, 1x HBSS, 0.3 % bovine serum albumine (BSA), pH 7.4) at 3× drug concentration. Plates were decanted and received 20 μL per well of drug buffer (20 mM HEPES, 1x HBSS) followed by addition of 10 μL of drug solution (3 wells per condition) for 15 min in the dark at room temperature. To stimulate endogenous cAMP via β adrenergic‐Gs activation, 10 μL luciferin (4 mM final concentration) supplemented with isoproterenol (400 nM final concentration) were added per well. Cells were again incubated in the dark at room temperature for 15 min, and luminescence intensity was quantified using a Wallac TriLux microbeta (Perkin Elmer) luminescence counter. Results (relative luminescence units) were plotted as a function of drug concentration, normalized to % SalA stimulation, and analyzed using “log(agonist) vs. response” in GraphPad Prism 5.0.

#### Tango β‐arrestin‐2 recruitment assay

The KOR Tango constructs were designed and assays were performed as previously described.^45^, ^56^ HTLA cells expressing TEV fused‐β‐arrestin2 (kindly provided by Dr. Richard Axel, Columbia University) were transfected with the KOR Tango construct. The next day, cells were plated in DMEM supplemented with 1 % dialyzed FBS in poly‐L‐lysine coated 384‐well white clear bottom cell culture plates at a density of 10 000–15 000 cells/well in a total of 40 μL. The cells were incubated for at least 6 h before receiving drug stimulation. Drug solutions were prepared in drug buffer (20 mM HEPES, 1x HBSS, 0.3 % BSA, pH 7.4) at 3× and added to cells (20 μL per well) for overnight incubation. Drug solutions used for the Tango assay were exactly the same as used for the cAMP assay. The next day, medium and drug solutions were removed and 20 μL per well of BrightGlo reagent (purchased from Promega, after 1 : 20 dilution) was added. The plate was incubated for 20 min at room temperature in the dark before being counted using a luminescence counter. Results (relative luminescence units) were plotted as a function of drug concentration, normalized to % U‐50488 stimulation, and analyzed using “log(agonist) vs. response” in GraphPad Prism 5.0.

### *In vitro* studies to assess the anti‐inflammatory activity in immune cells

#### Immune cell isolation and stimulation

C57BL/6 mice (wild‐type; purchased from Janvier Labs, Le Genest‐Saint‐Isle, France) and kappa‐opioid receptor deficient mouse mutants (KOR^−/−^, purchased from The Jackson Laboratory, Bar Harbor, ME) were used at the age of 8 to 12 weeks and housed under specific pathogen‐free conditions in microisolator cages. Mice were given chow and water *ad libitum* and animal experiments were performed with the approval of the State Review Board of North Rhine‐Westphalia according to the German law for animal welfare (reference numbers 81‐02.05.50.17.015). After sacrifice of mice peripheral lymph nodes, femurs and tibias were removed to isolate T cells or bone marrow cells (see below). Single cell suspensions of mouse lymph nodes were prepared according to standard methods. Subsequently, total T cells were isolated from cell suspensions using the Pan T Cell Isolation Kit II (Miltenyi Biotech, Bergisch Gladbach, Germany), activated for 12 h with **phorbol** 12‐myristate 13‐acetate (PMA, 5 ng/mL) and ionomycin (500 ng/mL) and cultured for 48 h in the presence of compounds **12 b**, **12 c**, **14 b** and **14 c** (1 μg/mL or 5 μg/mL each) or PBS as a control. Finally, cells were subjected to flow cytometry or cytokine quantification assays (see below).

For the generation of DC, bone marrow (bm) was collected from tibias and femurs of wild‐type and KOR^−/−^ mice, single cell suspensions were prepared and cultured in the presence of 150 U/mL GM‐CSF and 75 U/mL IL‐4 (Biotechne, Minneapolis, MN) for 9 days. From day 7 to day 9 of culture, cells were stimulated with PMA (5 ng/mL) and ionomycin (500 ng/mL) in the presence or absence of compounds **12b**, **12c**, **14b** or **14c** (1 μg/mL or 5 g/mL each). On day 9, bmDC were harvested and used for flow cytometry analyses (see below).

Human DC and T cells were isolated from peripheral blood mononuclear cells (PBMC). Therefore, PBMC were purified from fully anonymized leukapheresis reduction chambers, obtained from the blood bank with the informed consent of healthy donors by Ficoll gradient centrifugation according to standard methods (Ficoll reagent was purchased from Merck, Darmstadt, Germany). Total DC or T cells were negatively enriched using the Pan‐DC Enrichment Kit or the Pan T Cell Isolation Kit (Miltenyi Biotech). All experiments were carried out according to the declaration of Helsinki and were approved by the ethical committee of the University of Münster Medical School (2008‐180‐f‐S). After isolation, human DC and T cells were activated for 12 h with PMA (5 ng/mL) and ionomycin (500 ng/mL) and cultured for additional 48 h in the presence of compounds **12b**, **12c**, **14b** and **14c** (1 or 5 g/mL each) or PBS (as a control) and subjected to flow cytometry analyses or cytokine quantification (see below).

#### Multicolor flow cytometry

The expression of cell surface and intracellular markers was analyzed by multicolor flow cytometry on a Gallios^TM^ flow cytometer (Beckman Coulter, Krefeld, Germany) using the Kaluza software. For flow cytometry, mouse cells were stained in PBS using antibodies against CD3ϵ (clone 145‐2C11), CD25 (clone PC61), MHC‐II (major histocompatibility complex II; clone M5/114), CD69 (clone H1.2F3), F4/80 (clone BM8), and CD19 (clone 6D5), CD44 (clone IM7) and CD11c (clone N418); all purchased from Biolegend. Intracellular staining of IFN‐γ (clone XMG1.2) was performed after cell permeabilization using the Fix/Perm Buffer Set (Biolegend) according to the manufacturer's instructions.

Human cells were stained in PBS using antibodies against CD3 (clone OKT3), CD25 (clone BC96), HLA‐DR (clone L243), CD69 (clone FN50) and CD11c (clone 3.9); all purchased from Biolegend. Intracellular staining of IFN‐γ (clone 4S.B3) was performed after cell permeabilization using the Fix/Perm Buffer Set (Biolegend) according to the manufacturer's instructions. Isotype‐matched controls were included in each staining and apoptotic cells were identified using an annexin V apoptosis assay kit (Abcam, Cambridge, UK).

#### Cytokine quantification

Cytokine concentrations in cell culture supernatants of T cells or DC treated with **12b**, **12c**, **14b** or **14c** were quantified using LEGENDplex™ kits (Biolegend, San Diego, CA) according to the manufacturer's instructions. All samples were analyzed on a Gallios™ flow cytometer using the LEGENDplex™ Data Analysis Software (Biolegend) and cytokine concentrations were determined according to standard curves.

#### Statistics

All values are expressed as means ±SD. Statistically significant differences were assessed by one‐way analysis of variance (ANOVA) test, comparing more than two groups. The alpha‐level was set at a value of <0.05 in most and at a value of <0.01 in exceptional cases (e. g., Figure 3B) SigmaPlot 14 or GraphPad Prism 8 was used to analyze, plot, and illustrate data.

### Radiosynthesis

#### General

The first step of the radiosynthesis was carried out on a modified PET tracer radiosynthesiser (TRACERLab Fx_FDG_, GE Healthcare). The recorded data were processed by the TRACERLab Fx software (GE Healthcare). Separation and purification of the radiosynthesized compound [^18^F]‐**2** were performed on the following semipreparative radio‐HPLC system A1: K‐500 and K‐501 pump, K‐2000 UV detector (Herbert Knauer GmbH), NaI(TI) Scintibloc 51 SP51 γ‐detector (Crismatec) and an ACE 5 AQ column (250 mm×10 mm). Method A1 started with a linear gradient from 0 to 100 % CH_3_CN in water (0.1 % TFA) over 45 min, holding for 5 min and followed by a linear gradient from 100 to 0 % CH_3_CN in water (0.1 % TFA) over 5 min, with λ=220 nm and a flow rate of 5.5 mL ⋅ min^−1^. Separation and purification of the radiosynthesized compound [^18^F]‐**14c** were performed on the following semipreparative radio‐HPLC system A2: two Variopumps (Scintomics), Smartline UV detector 2500 (Herbert Knauer GmbH), a GabiStar γ‐detector (Raytest Isotopenmessgeräte GmbH) and an ACE 5 AQ column (250 mm×10 mm). Method A2 started with a linear gradient from 20 % to 35 % CH_3_CN in water (0.1 % TFA) over 45 min and then from 35 % to 100 % in 5 min, followed by a linear gradient from 100 to 20 % CH_3_CN in water (0.1 % TFA) over 5 min, with *λ*=220 nm and a flow rate of 5.5 mL ⋅ min^−1^. Radiochemical purities and specific activities were determined using the analytical radio‐HPLC system B: Two Smartline 1000 pumps and a Smartline UV detector 2500 (Herbert Knauer GmbH), a GabiStar γ‐detector (Raytest Isotopenmessgeräte GmbH) and a Nucleosil 100‐5C‐18 column (250 mm×4 mm). Method B started with a linear gradient from 10 to 100 % CH_3_CN in water (0.1 % TFA) over 15 min, holding for 3 min followed by a linear gradient from 100 % to 10 % CH_3_CN in water (0.1 % TFA) over 2 min, with λ=210 nm and a flow rate of 1.0 mL ⋅ min^−1^. The recorded data of both HPLC‐systems were processed by the GINA Star software (Raytest Isotopenmessgeräte GmbH). No‐carrier‐added aqueous [^18^F]fluoride was produced on a RDS 111e cyclotron (CTI‐Siemens) by irradiation of a 2.8 mL water target using 10 MeV proton beams on 97.0 % enriched [^18^O]H_2_O by the ^18^O(p,n)^18^F nuclear reaction. After unloading the target, it was rinsed with ultrapure water (2.0 mL). This rinsed water containing [^18^F]fluoride ions was used for the radiosyntheses.

#### Radiosynthesis of ^18^F‐labeled tracers [^18^F]‐2 and [^18^F]‐14c

##### 2‐(3,4‐Dichlorophenyl)‐1‐((4a*R*,8*R*,8a*S*)‐ and ((4a*S*,8*S*,8a*R*)‐ 4‐{[1‐(2‐[^18^F]fluoroethyl)‐1*H*‐1,2,3‐triazol‐4‐yl]methyl}‐8‐ (pyrrolidin‐1‐yl)‐3,4,4 a,5,6,7,8,8 a‐octahydroquinoxalin‐ 1(*2H*)‐yl)ethan‐1‐one ([^18^F]‐2)

In a computer controlled TRACERLab Fx_FDG_ Synthesizer the batch of aqueous [^18^F]fluoride ions (2280–4771 MBq) from the cyclotron target was passed through an anion exchange resin (pre‐conditioned Sep‐Pak® Light QMA cartridge with CO_3_^2−^ as counter ion). [^18^F]Fluoride ions were eluted from the resin to the reactor with a mixture of 1 M K_2_CO_3_ (40 μL), water for injection (200 μL), and DNA‐grade CH_3_CN (800 μL) containing Kryptofix®2.2.2 (K222) (20 mg, 53 μmol). Subsequently, the aqueous K(K222)[^18^F]F solution was carefully evaporated to dryness *in vacuo*.

The precursor 2‐azidoethyl 4‐methylbenzenesulfonate (20 mg, 83 μmol) in DNA‐grade CH_3_CN (500 μL) was added and the mixture was heated at 110 °C for 3 min. The radiolabeled synthon 1‐azido‐2‐[^18^F]fluoroethane was distilled from the reactor into a 5 mL flask that contained 0.4 M CuSO_4_ ⋅ 5H_2_O aqueous solution (120 μL), sodium ascorbate (16 mg, 81 μmol) in water for injection (100 μL) and alkyne **18** (5.0 mg, 11.5 μmol) in DMF (400 μL) and the mixture was cooled to −10 °C. After 30 min stirring at 40 °C, the mixture was passed through a Waters Sep‐Pak Light cartridge filled with quartz wool. The cartridge was rinsed with DMF (200 μL) and the resulting clear solution was purified by gradient‐radio‐HPLC system A1, method A1. The fraction containing compound [^18^F]‐**2** (*t*_R_([^18^F]‐**2**)=25.5 min) was evaporated to dryness *in vacuo* and formulated in water for injection/EtOH (9 : 1 *v/v*; 1 mL). [^18^F]‐**2** was obtained in an overall rcy of 49.9±8.6 % (d.c., based on cyclotron‐derived [^18^F]fluoride ions, *n*=5) in 136±12 min from the end of radionuclide production. [^18^F]‐**2** was isolated in rcp of 99.3±0.4 % with *A*_m_ in the range of 4.6–14.6 GBq/μmol at the end of the synthesis. Rcp and *A*_m_ of [^18^F]‐**2** (*t*_R_([^18^F]‐**2**)=8.98–9.15 min) were determined by analytical radio‐HPLC B, method B. Radiochemical identity of [^18^F]‐**2** was determined by co‐elution of this compound with the non‐radioactive counterpart **2**, that was added to a preparation of [^18^F]‐**2**, at the analytical radio‐HPLC B (method B) detected by the γ detector ([^18^F]‐**2**) and the UV detector (**2**), respectively.

##### Methyl (4a*R*,5*S*,8a*S*)‐ and (4a*S*,5*R*,8a*R*)‐4‐[2‐(3,4‐dichlorophenyl)acetyl]‐5‐[(*R*)‐3‐[^18^F]fluoropyrrolidin‐1‐yl]‐3,4,4 a,5,6,7, 8,8 a‐octahydroquinoxaline‐1(2*H*)‐carboxylate ([^18^F]‐14c)

Separation of [^18^F]fluoride, formation and evaporation of K(K222)[^18^F]F in the TRACERLab Fx_FDG_ Synthesiser were performed as described in the procedure for the preparation of [^18^F]‐**2** (see above). A solution of the precursor mesylate **19** (5.0 mg, 9.1 μmol) in DNA‐grade CH_3_CN (1.0 mL) was added and the mixture was heated at 110 °C for 20 min. Then, the solution was cooled to 55 °C, water for injection (10 mL) was added and the mixture was passed through a Waters Sep‐Pak C_18_ Light cartridge (preconditioned with 10 mL ethanol and 10 mL water). The cartridge was washed with water for injection (10 mL) and eluted with a DMF (0.5 mL) that was preheated to 100 °C. The eluate was diluted with water for injection (0.5 mL) and this product solution was purified by gradient‐radio‐HPLC system A2 (method A2). The fraction containing compound [^18^F]‐**14c** (*t*_R_([^18^F]‐**14c**)=35.0 min) was evaporated to dryness *in vacuo* and formulated in water for injection/EtOH (9 : 1, *v/v*; 1 mL). [^18^F]‐**14c** was obtained in rcy of 20.7±3.4 % (*n*=5; d.c. based on cyclotron‐derived [^18^F]fluoride ions) in 128±14 min from the end of radionuclide production. The target compound was isolated rcp of 98.9±0.7 % with *A*_m_ of 4.0–111.3 GBq/μmol at the end of the synthesis. Rcp and *A*_m_ of [^18^F]‐**14c** (*t*_R_([^18^F]‐**14c**)=11.33–11.58 min) were determined by analytical radio‐HPLC B, method B. Radiochemical identity of [^18^F]‐**14c** was determined analogously to [^18^F]‐**2**.

### Determination of the distribution coefficient logD_7.4_

The lipophilicity of radioligands [^18^F]‐**2** and [^18^F]‐**14c** was assessed by determination of the water *n*‐octan‐1‐ol distribution coefficient (logD) following a published procedure.^57^ In brief, approximately 3.2 MBq of radioligand [^18^F]‐**2** and approximately 0.3 MBq of radiotracer [^18^F]‐**14c**, respectively, were mixed with equal amounts (0.6 mL) of phosphate buffered saline (PBS, pH 7.4) and *n*‐octan‐1‐ol. The resulting biphasic system was mixed vigorously for 3 min at room temperature and subsequently, the tubes were centrifuged (≥5 min). Then 400 μL of the *n*‐octan‐1‐ol layer was separated and 400 μL of PBS were added. The mixture was mixed vigorously for 10 min at room temperature followed by centrifugation of the tubes for≥5 min. Two samples of 100 μL of each layer were counted in a γ‐counter (2480 Automatic Gamma Counter Wizard^2^, Perkin‐Elmer). The distribution coefficient was determined by calculating the ratio cpm(*n*‐octan‐1‐ol)/cpm(PBS) and expressed as log*D*_*7.4*_ (log(cpm_*n*‐octanol_/cpm_PBS_)). Three independent experiments were performed in duplicate and data were provided as mean values ±standard deviation.

### Animal experiments

All animal experiments were performed in accordance with the legal requirements of the European Community (Directive 2010/63/EU) and the corresponding German Animal Welfare Law (TierSchG, TierSchVersV) and were approved by the local authorizing agency (State Office for Nature, Environment and Consumer Protection North Rhine‐Westphalia; in vivo biodistribution studies: permit no. 84‐02. 04. 2013.A046 and 84‐02.05.30.12.A084.

### Stability of [^18^F]‐2 and [^18^F]‐14c in mouse and human serum *(in vitro)*

The serum stability of radioligands [^18^F]‐**2** and [^18^F]‐**14c** was evaluated by incubation in mouse or human serum at 37 °C for up to 90 min. An aliquot of formulated [^18^F]‐**2** or [^18^F]‐**14c** (20 μL, ca. 6.6 MBq and ca.10 MBq, respectively) was added to a sample of mouse serum (200 μL) or human serum (200 μL), and the mixtures were incubated at 37 °C. Samples of 20 μL each were taken after periods of 10, 30, 60 and 90 min and ice‐cold DNA‐grade CH_3_CN (100 μL) were added. After centrifugation for ≥5 min, the supernatant was analyzed by analytical radio‐HPLC B, method B.

### *In vivo* Biodistribution studies of [^18^F]‐2and [^18^F]‐14c

Adult C57BL/6 mice (*n*=2 male, 15 weeks, 28 g for [^18^F]‐**2**; *n*=2, male, 10 weeks, 25 g for [^18^F]‐**14c**) were anesthetized by isoflurane/O_2_ and one lateral tail vein was cannulated BY using a 27 G needle connected to 15 cm polyethylene catheter tubing. Radiotracers (∼500 kBq/g bodyweight, 15 MBq) were injected as a bolus (50 μL compound flushed with 100 μL saline) *via* the tail vein and subsequent PET scanning was performed.

Small Animal PET Scanning. PET experiments were carried out using a submillimeter high resolution (0.7 mm full width at half‐maximum) small animal scanner (32 module quadHIDAC, Oxford Positron Systems Ltd., Oxford, UK) with uniform spatial resolution (<1 mm) over a large cylindrical field (165 mm diameter, 280 mm axial length). List‐mode data were acquired for 90 min and reconstructed into dynamic time frames using an iterative reconstruction algorithm. Subsequently, the scanning bed was transferred to the computed tomography (CT) scanner (Inveon, Siemens Medical Solutions, U.S.), and a CT acquisition with a spatial resolution of 80 μm was performed for each mouse. Reconstructed image data sets were coregistered based on extrinsic markers attached to the multimodal scanning bed and the in‐house developed image analysis software MEDgical. Three‐dimensional volumes of interest (VOIs) were defined over the respective organs in CT data sets, transferred to the coregistered PET data, and analyzed quantitatively. Regional uptake was calculated as percentage of injected dose by dividing counts per milliliter in the VOI by total counts in the mouse multiplied by 100 (%ID/mL).

### Biostability and biotransformation of [^18^F]‐2 in mice (*in vivo*)

Approximately 72 MBq of [^18^F]‐**2** (in a volume of 100 μL) were injected into the tail vein of each of three 11‐week‐old C57/BL6 mice. The animals were sacrificed 90 min after injection. Whole blood of the three mice was pooled (∼2 mL) and centrifuged (3 min) to isolate plasma. Plasma (∼0.7 mL) was separated and mixed with ice‐cold CH_3_CN (1.4 mL). After further centrifugation for 5 min, the supernatant was separated and evaporated to dryness *in vacuo* at moderate temperatures (≤60 °C). The residue was dissolved in water for injection (20 μL) and the solution was analyzed by analytical radio‐HPLC B, method B.

### Biotransformation of 14c using mouse liver microsomes (*in vitro*)

#### LC‐MS setup (Method LC‐MS)

For the determination of exact masses and for conducting MS/MS experiments, an LC system was coupled with a quadrupole time‐of‐flight (qToF) mass spectrometer.

**HPLC‐DAD** (Thermo Fisher Scientific): Solvent rack (SRD 3600); pump (DGP‐3600RS); autosampler (WPS‐3000RS); column oven (TCC‐3000RS); precolumn: SecurityGuardTM Cartridge AQ C18 (4.0×2.0 mm, 4.0 μm particle size); column: SynergiTM Hydro‐RP (50×2.1 mm, 2.5 μm particle size, Phenomenex®, Aschaffenburg, Germany); temperature: 30 °C and DAD‐detector (DAD‐3000RS). The LC system was coupled with a micrOTOF‐Q II (Bruker Daltonics, Bremen, Germany). The ESI‐qToF was operated in positive ion polarity in the full scan mode (*m/z* 70–700, 200–1000 or 500–1600) with the following settings: capillary voltage 4500 V; end plate offset −500 V; collision cell RF 300.0 Vpp; nebulizer 2.0 bar; dry heater 200 °C; dry gas 9.0 L/min. To protect the MS from salts or other components of the matrices, a six‐port valve was used to elute the first 2.0 min of each run into the waste (cut‐off). In case of MS/MS experiments the isolation window of the first quadrupole was set to 10 *m/z* units (for *m/z*<600) or 20 *m/z* units (for m/z>600). The collision energy of the second quadrupole was set in the range of 10–35 eV and is given for each experiment. For data handling and control of the system the software Data Analysis and Hystar from Bruker Daltonics (Bremen, Germany) was used. The calibration of the ToF spectra was achieved by injection of LiHCO_2_ (m/z<700, *i*‐propanol/H_2_O 1 : 1, 10 mM) via a 20 μL sample loop within each LC run at 2.0–2.2 min.

**LC parameters**: mobile phase A: H_2_O/CH_3_CN 90 : 10+0.1 % FA; mobile phase B: CH_3_CN/H_2_O 90 : 10+0.1 % FA; mobile phase C: H2O+0.1 % FA; pump 1: flow rate: 0–3 min: 0.1 mL/min, 3–3.1 min: from 0.1 mL/min to 0.4 mL/min, 3.1–17.9 min: 0.4 mL/min, 17.9–18 min: from 0.4 mL/min to 0.1 mL/min; gradient elution: (A%): 0–3.1 min: 100 %, 3.1–12 min: gradient from 100 % to 0 %, 12–14.5 min: 0 %, 14.5–15 min: gradient from 0 % to 100 %, 15–18 min: 100 %; pump 2: flow rate: 0–3 min: 0.3 mL/min, 3–3.1 min: from 0.3 mL/min to 0.0 mL/min, 3.1–17.9 min: 0.0 mL/min, 17.9–18 min: from 0.0 mL/min to 0.3 mL/min; isocratic: (C %): 0–18 min: 100 %.

#### Experimental procedure

Mouse liver microsomes (7.8 mg protein/mL) were added to an Eppendorf cap filled with sodium phosphate buffer pH 7.4 (PBS, 0.1 M), MgCl_2_ solution (0.05 M), NADPH (2 mg/mL in PBS) and DMSO stock solution, giving a total volume of 200 μL. Final concentrations for the incubations were 75 mM PBS, 0.6 mM NADPH, 1 mg/mL microsomal protein, 50 μM of the respective compound, 12.5 mM MgCl_2_ and 0.5 % DMSO. For murine serum stability, the respective compound (1 μL, 10 mM DMSO stock solution) was incubated in murine serum (black six mice, 199 μL). The suspension was mixed vigorously and incubated (37 °C, 90 min, 900 rpm). Subsequently, the incubation was stopped by addition of CH_3_CN/CH_3_OH (1 : 1, 400 μL), the caps were cooled down (0 °C, 10 min) and the precipitated proteins were separated by centrifugation (4 °C, 15 min, 16000 rpm). Afterward, the supernatant was analyzed by the LC‐MS method described above. With the same procedure, the empty value (without stock solution), blank value (without NADPH), buffer sample (parent incubated in PBS solution) and negative control (solvent (599 μL) and DMSO stock solution (1 μL)) were prepared.

## Supporting Information

Supporting Information contains details on the receptor binding studies, quality control of [^18^F]‐**2** and its stability in blood serum, investigation of *in vivo* radiometabolites of [^18^F]‐**2**, distribution of the PET tracer [^18^F]‐**14c** in various organs, *in vitro* biotransformation of **14c**, identification of metabolites of **14c** formed in vitro and purity data of all test compounds. Moreover, the ^1^H and ^13^C NMR spectra of all synthesized compounds are included. Molecular formula strings are added.

## Abbreviations

*A*_m_: molar activity
APCI: atmospheric pressure chemical ionization
BBB: blood brain barrier
bm: bone marrow
bmDC: bone marrow‐derived dendritic cells
cpm: counts per minute
CSF: cerebrospinal fluid
CT: computer tomography
d.c.: decay corrected
DC: dendritic cells
DOR: *δ*‐opioid receptor
EAE: experimental autoimmune encephalomyelitis
H&E: hematoxylin and eosin
INF‐β: interferone β
INF‐γ: interferone γ
IL‐10: interleukin‐10
IL‐17: interleukin‐17
Iono: ionomycin
KOR: κ‐opioid receptor
*l*: length of the stationary phase
MOG: myelin oligodendrocyte glycoprotein
MOR: μ‐opioid receptor
MS: multiple sclerosis
OPC: oligodendrocyte progenitor cells
PBMC: peripheral blood mononuclear cells
PBS: phosphate buffered saline
PET: positron emission tomography
PMA: phorbol 12‐myristate 13‐acetate
PML: progressive multifocal leukoencephalopathy
rcp: radiochemical purity
rcy: radiochemical yield
TBAF: tetrabutylammonium fluoride
TBDPS: *tert*‐butyldiphenylsilyl
T_H_1: T helper cells 1
T_H_17: T helper cells 17
Treg: regulatory T cells
*V*: fraction size
VOI: volume of interest
WT: wild‐type

## Conflict of interest

The authors declare no conflict of interests.

## Supporting information

As a service to our authors and readers, this journal provides supporting information supplied by the authors. Such materials are peer reviewed and may be re‐organized for online delivery, but are not copy‐edited or typeset. Technical support issues arising from supporting information (other than missing files) should be addressed to the authors.

Supplementary

## References

[1] G. W. Pasternak , Y.-X. Pan , Pharmacol. Rev. 2013, 65, 1257–1317. 24076545 10.1124/pr.112.007138 PMC3799236

[2] G. Pasternak , Y. X. Pan , Acta Anaesthesiol. Taiwanica 2011, 49, 21–25. 10.1016/j.aat.2010.12.008 PMC4014005 21453899

[3] B. N. Dhawan , F. Cesselin , R. Raghubir , T. Reisine , P. B. Bradley , P. S. Portoghese , M. Hamon , Pharmacol. Rev. 1996, 48, 567–592. 8981566

[4] T. W. Vanderah , Clin. J. Pain 2010, 26 (suppl.10), 10–15. 10.1097/AJP.0b013e3181c49e3a 20026960

[5] C. Chavkin , Neuropsychopharmacology 2011, 36, 369–370. 21116263 10.1038/npp.2010.137 PMC3055513

[6] M. Rios , Neuropsychopharmacology 2011, 36, 368–369. 21116262 10.1038/npp.2010.139 PMC3055507

[7] S. D. Mague , A. M. Pliakas , M. S. Todtenkopf , H. C. Tomasiewicz , Y. Zhang , W. C. Jr Stevens , R. M. Jones , P. S. Portoghese , W. A. Jr Carlezon , Pharmacol. Ther. 2003, 305, 323–330. 10.1124/jpet.102.046433 12649385

[8] M. D. Metcalf , A. Coop , AAPS J. 2005, 7, Article 71, E704–E271. 10.1208/aapsj070371 PMC2751273 16353947

[9] A. S. Agostimho , M. Mietzsch , L. Zangrandi , I. Kmec , A. Mutti , L. Kraus , P. Fidzinski , U. C. Schneider , M. Holtkamp , R. Heilbronn , C. Schwarzer , EMBO Mol. Med. 2019, 11, e9963. 31486590 10.15252/emmm.201809963 PMC6783645

[10] L. Lalanne , G. Ayranci , B. L. Kieffer , P. E. Lutz , Front. Psychiatry 2014, 5, 1–17. 25538632 10.3389/fpsyt.2014.00170 PMC4258993

[11] J. L. Lynch , J. F. Alley , L. Wellman , A. J. Beitz , Brain Res. 2008, 1191, 180–191. 18096140 10.1016/j.brainres.2007.11.034 PMC2258219

[12] C. Du , Y. Duan , W. Wei , Y. Cai , H. Chai , J. Lv , X. Du , J. Zhu , X. Xie , Nat. Commun. 2016, 7, 11120. 27040771 10.1038/ncomms11120 PMC4822006

[13] G. Tangherlini , D. V. Kalinin , D. Schepmann , T. Che , N. Mykicki , S. Ständer , K. Loser , B. Wünsch , J. Med. Chem. 2019, 62, 893–907. 30543421 10.1021/acs.jmedchem.8b01609

[14] P. S. Talbot , R. Narendran , E. R. Butelman , Y. Huang , K. Ngo , M. Slifstein , D. Martinez , M. Laruelle , D.-R. Hwang , J. Nucl. Med. 2005, 46, 484–494. 15750163

[15] A. G. Hayes , P. J. Birch , N. J. Hayward , M. J. Sheehan , H. Rogers , M. B. Tyers , D. B. Judd , D. I. Scopes , A. Naylor , Br. J. Pharmacol. 1990, 101, 944–948. 1964823 10.1111/j.1476-5381.1990.tb14185.x PMC1917842

[16] N. B. Nabulsi , M.-Q. Zheng , J. Ropchan , D. Labaree , Y.-S. Ding , L. Blumberg , Y. Huang , Nucl. Med. Biol. 2011, 38, 215–221. 21315277 10.1016/j.nucmedbio.2010.08.014

[17] B. W. Schoultz , T. Hjornevik , F. Willoch , J. Marton , A. Noda , Y. Murakami , S. Miyoshi , S. Nishimura , E. Årstad , A. Drzezga , I. Matsunari , G. Henriksen , Eur. J. Nucl. Med. Mol. Imaging 2010, 37, 1174–1180. 20157708 10.1007/s00259-010-1384-6

[18] M. Naganawa , L. K. Jacobsen , M.-Q. Zheng , S.-F. Lin , A. Banerjee , W. Byon , D. Weinzimmer , G. Tomasi , N. Nabulsi , S. Grimwood , L. L. Badura , R. E. Carson , T. J. McCarthy , Y. Huang , NeuroImage 2014, 99, 69–79. 24844744 10.1016/j.neuroimage.2014.05.033 PMC4140089

[19] M. S. Placzek , G. C. Van de Bittner , H.-Y. Wey , S. E. Lukas , J. M. Hooker , Neuropsychopharmacology, 2015, 40, 1–8. 26058662 10.1038/npp.2015.159 PMC4864638

[20] J. M. Miller , F. Zanderigo , P. D. Purushothaman , C. DeLorenzo , H. Rubin-Falcone , R. T. Ogden , J. Keilp , M. A. Oquendo , N. Nabulsi , Y. H. Huang , R. V. Parsey , R. E. Carson , J. J. Mann , Synapse, 2018, 72, e22042. 29935119 10.1002/syn.22042 PMC7599086

[21] S. Li , M.-Q. Zheng , M. Naganawa , S. Kim , H. Gao , M. Kapinos , D. Labaree , Y. Huang , J. Nucl. Med. 2019, 60, 1023–1030. 30630942 10.2967/jnumed.118.220517 PMC6604690

[22] M.-Q. Zheng , N. Nabulsi , S. J. Kim , G. Tomasi , S.-F. Lin , C. Mitch , S. Quimby , V. Barth , K. Rash , J. Masters , A. Navarro , E. Seest , E. D. Morris , R. E. Carson , Y. Huang , J. Nucl. Med. 2013, 54, 455–463. 23353688 10.2967/jnumed.112.109512 PMC3775344

[23] C. H. Mitch , S. J. Quimby , N. Diaz , C. Pedregal , M. G. De La Torre , A. Jimenez , Q. Shi , E. J. Canada , S. D. Kahl , M. A. Statnick , D. L. McKinzie , D. R. Benesh , K. S. Rash , C. N. Barth , J. Med. Chem. 2011, 54, 8000–8012. 21958337 10.1021/jm200789r

[24] M. Naganawa , M.-Q. Zheng , S. Henry , N. Nabulsi , S.-F. Lin , J. Ropchan , D. Labaree , S. Najafzadeh , M. Kapinos , J. Tauscher , A. Neumeister , R. E. Carson , Y. Huang , J. Nucl. Med. 2015, 56, 243–248. 25593119 10.2967/jnumed.114.147975 PMC4322754

[25] M. Naganawa , G. L. Dickinson , M. Q. Zheng , S. Henry , F. Vandenhende , J. Witcher , R. Bell , N. Nabulsi , S. F. Lin , J. Ropchan , A. Neumeister , M. Ranganathan , J. Tauscher , Y. Huang , R. E. Carson , J. Pharmacol. Exp. Ther. 2016, 356, 260–266. 26628406 10.1124/jpet.115.229278 PMC4727157

[26] S. J. Kim , M.-Q. Zheng , N. Nabulsi , D. Labaree , J. Ropchan , S. Najafzadeh , R. E. Carson , Y. Huang , E. D. Morris , J. Nucl. Med. 2013, 54, 1668–1674. 23918735 10.2967/jnumed.112.118877 PMC5824998

[27] Z. Cai , S. Li , R. Pracitto , A. Navarro , A. Shirali , J. Ropchan , Y. Huang , ACS Chem. Neurosci. 2017, 8, 12–16. 27741398 10.1021/acschemneuro.6b00268

[28] S. Li , Z. Cai , M.-Q. Zheng , D. Holden , M. Naganawa , S.-F. Lin , J. Ropchan , D. Labaree , M. Kapinos , T. Lara-Jaime , A. Navarro , Y. Huang , J. Nucl. Med. 2018, 59, 140–16. 28747521 10.2967/jnumed.117.195586 PMC5750518

[29] M. Naganawa , S. Li , N. Nabulsi , S. Najafzadeh , H. Gao , Z. Cai , M. Kapinos , J. Ropchan , R. Carson , Y. Huang , J. Nucl. Med. 2019, 60, supplement 1, 577.30602593

[30] G. Poinsel , F. Oueslati , M. Dhilly , J. Delamare , C. Perrio , D. Debruyne , L. Barré , Nucl. Med. Biol. 2008, 35, 561–569. 18589300 10.1016/j.nucmedbio.2008.02.010

[31] S. Schmitt , N. Colloc'h , C. Perrio , Eur. J. Med. Chem. 2015, 90, 742–750. 25513968 10.1016/j.ejmech.2014.12.016

[32] S. Schmitt , J. Delamare , O. Tirel , F. Fillesoye , M. Dhilly , C. Perrio , Nucl. Med. Biol. 2016, 44, 50–61. 27821345 10.1016/j.nucmedbio.2016.09.005

[33] P. Molenveld , R. Bouzanne Des Mazery , G. J. Sterk , R. P. M. Storcken , R. Autar , B. Van Oss , R. N. S. Van Der Haas , R. Fröhlich , D. Schepmann , B. Wünsch , M. Soeberdt , Bioorg. Med. Chem. Lett. 2015, 25, 5326–5330. 26411794 10.1016/j.bmcl.2015.09.040

[34] M. Soeberdt , P. Molenveld , R. P. M. Storcken , R. Bouzanne Des Mazery , G. J. Sterk , R. Autar , M. G. Bolster , C. Wagner , S. N. H. Aerts , F. R. Van Holst , A. Wegert , G. Tangherlini , B. Frehland , D. Schepmann , D. Metze , T. Lotts , U. Knie , K. Y. Lin , T. Y. Huang , C. C. Lai , S. Ständer , B. Wünsch , C. Abels , J. Med. Chem. 2017, 60, 2526–2551. 28218838 10.1021/acs.jmedchem.6b01868

[35] C. Bourgeois , E. Werfel , F. Galla , K. Lehmkuhl , H. Torres-Gómez , D. Schepmann , B. Kögel , T. Christoph , W. Straßburger , W. Englberger , M. Soeberdt , S. Hüwel , H. J. Galla , B. Wünsch , J. Med. Chem. 2014, 57, 6845–6860. 25062506 10.1021/jm500940q

[36] C. Wittig , D. Schepmann , M. Soeberdt , C. G. Daniliuc , B. Wünsch , Org. Biomol. Chem. 2017, 15, 6520–6540. 28745376 10.1039/c7ob01530e

[37] C. Geiger , C. Zelenka , K. Lehmkuhl , D. Schepmann , W. Englberger , B. Wünsch , J. Med. Chem. 2010, 53, 4212–4222. 20441176 10.1021/jm100182p

[38] D. Kracht , E. Rack , D. Schepmann , R. Fröhlich , B. Wünsch , Org. Biomol. Chem. 2010, 8, 212–225. 20024152 10.1039/b915180j

[39] B. R. Costa , W. D. De Bowen , S. B. Hellewell , C. George , R. B. Rothman , A. A. Reid , J. M. Walker , A. E. Jacobson , K. C. Rice , J. Med. Chem. 1989, 32, 1996–2002. 2547074 10.1021/jm00128a050

[40] L. Radesca , W. D. Bowen , L. Di Paolo , B. R. de Costa , J. Med. Chem. 1991, 34, 3058–3065. 1656044 10.1021/jm00114a015

[41] P. Hasebein , B. Frehland , K. Lehmkuhl , R. Fröhlich , D. Schepmann , B. Wünsch , Org. Biomol. Chem. 2014, 12, 5407–5426. 24934693 10.1039/c4ob00510d

[42] C. Meyer , B. Neue , D. Schepmann , S. Yanagisawa , J. Yamaguchi , E.-U. Würthwein , K. Itami , B. Wünsch , Bioorg. Med. Chem. 2013, 21, 1844–1856. 23462714 10.1016/j.bmc.2013.01.038

[43] K. Miyata , D. Schepmann , B. Wünsch , Eur. J. Med. Chem. 2014, 83, 709–716. 25016157 10.1016/j.ejmech.2014.06.073

[44] Z. Zheng , X.-P. Huang , T. J. Mangano , R. Zou , X. Chen , S. A. Zaidi , B. L. Roth , R. C. Stevens , V. Katritch , J. Med. Chem. 2017, 60, 3070–3081. 28339199 10.1021/acs.jmedchem.7b00109 PMC5493393

[45] W. K. Kroeze , M. F. Sassano , X.-P. Huang , K. Lansu , J. D. McCorvy , P. M. Giguère , N. Sciaky , B. L. Roth , Nat. Struct. Mol. Biol. 2015, 22, 362–369. 25895059 10.1038/nsmb.3014 PMC4424118

[46] X. Liang , R. Liu , C. Chen , F. Ji , T. Li , Transl. Perioper. pain Med. 2016, 1, 5–13. PMC4790459 26985446

[47] Y. Tai , Q. Wang , H. Korner , L. Zhang , W. Wei , Front. Pharmacol. 2018, 9, 642. 29997500 10.3389/fphar.2018.00642 PMC6028573

[48] S. Sozzani , A. Del Prete , D. Bosisio , J. Autoimmune Dis. 2017, 85, 126–140. 10.1016/j.jaut.2017.07.012 28774715

[49] C. Reis e Sousa , Curr. Opin. Immunol. 2004, 16, 21–25. 14734106 10.1016/j.coi.2003.11.007

[50] O. Joffre , M. A. Nolte , R. Spörri , C. Reis e Sousa , Immunol. Rev. 2009, 227, 234–247. 19120488 10.1111/j.1600-065X.2008.00718.x

[51] M. Kunkl , S. Frascolla , C. Amormino , E. Volpe , L. Tuosto , Cells 2020, 9, E482. 32093011 10.3390/cells9020482 PMC7072830

[52] A. Rendon , K. Schäkel , Int. J. Mol. Sci. 2019, 20, E1475. 30909615 10.3390/ijms20061475 PMC6471628

[53] P. Yang , F. Y. Qian , M. F. Zhang , A. L. Xu , X. Wang , B. P. Jiang , L. L. Zhou , J. Leukocyte Biol. 2019, 106, 1233–1240. 31497905 10.1002/JLB.4RU0619-197R

[54] K. B. Park , N. R. Kitteringham , P. M. O'Neill , Annu. Rev. Pharmacol. Toxicol. 2001, 41, 443–4470. 11264465 10.1146/annurev.pharmtox.41.1.443

[55] T. Che , S. Majumdar , S. A. Zaidi , P. Ondachi , J. D. McCorvy , S. Wang , P. D. Mosier , R. Uprety , E. Vardy , B. E. Krumm , G. W. Han , M. Y. Lee , E. Pardon , J. Steyaert , X. P. Huang , R. T. Strachan , A. R. Tribo , G. W. Pasternak , F. I. Carroll , R. C. Stevens , V. Cherezov , V. Katritch , D. Wacker , B. L. Roth , Cell 2018, 172, 1–13. 10.1016/j.cell.2017.12.011 PMC5802374 29307491

[56] W. Liu , D. Wacker , C. Gati , G. W. Han , D. James , D. Wang , G. Nelson , U. Weierstall , V. Katritch , A. Barty , N. A. Zatsepin , D. Li , M. Messerschmidt , S. Boutet , G. J. Williams , J. E. Koglin , M. M. Seibert , C. Wang , S. T. A. Shah , S. Basu , R. Fromme , C. Kupitz , K. N. Rendek , I. Grotjohann , P. Fromme , R. A. Kirian , K. R. Beyerlein , T. A. White , H. N. Chapmann , M. Caffrey , J. C. H. Spence , R. C. Stevens , V. Cherezov , Science 2013, 342, 1521–1524. 24357322 10.1126/science.1244142 PMC3902108

[57] O. Prante , C. Hocke , S. Löber , H. Hübner , P. Gmeiner , T. Kuwert , Nuklearmedizin 2006, 45, 41–48. 16493513 10.1267/nukl06010041

