# Supplementary material for: Synthesis and Pharmacological Evaluation of Fluorinated Quinoxaline‐Based κ‐Opioid Receptor (KOR) Agonists Designed for PET Studies
Source: ChemMedChem. 2020 Sep 1;15(19):1834–53. doi: 10.1002/cmdc.202000502 (PMC7589326; doi:10.1002/cmdc.202000502)

ChemMedChem

Supporting Information

Synthesis and Pharmacological Evaluation of Fluorinated Quinoxaline-Based κ-Opioid Receptor (KOR) Agonists Designed for PET Studies

Giovanni Tangherlini, Frederik Börgel, Dirk Schepmann, Samuel Slocum, Tao Che, Stefan Wagner, Katrin Schwegmann, Sven Hermann, Nadine Mykicki, Karin Loser, and Bernhard Wünsch*

Table of Contents

Content	page
1. Receptor binding studies	S2
2. Quality control of [^{18}F]**2** and its stability in blood serum	S6
3. Investigation of *in vivo* radiometabolites of [^{18}F]**2**	S7
4. Distribution of the PET tracer [^{18}F]**14c** in various organs	S8
5. *in vitro* biotransformation of **14c** in the presence of mouse liver microsomes	S9
6. Identification of **14c** and its metabolites by LC-MS	S10
7. References	S13
8. NMR spectra	S14
9. Purity data of test compounds	S27
10. HPLC chromatograms of the test compounds	S28

1. Receptor binding studies

Materials

The guinea pig brains for KOR, MOR, and σ_1 receptor binding assays, rat brains for DOR binding assay and rat liver for σ_2 binding assay were commercially available (Harlan-Winkelmann, Borchen, Germany). Homogenizer: Elvehjem Potter (B. Braun Biotech International, Melsungen, Germany) and Soniprep 150, MSE, London, UK). Centrifuges: Cooling centrifuge model Rotina 35R (Hettich, Tuttlingen, Germany) and High-speed cooling centrifuge model Sorvall RC-5C plus (Thermo Fisher Scientific, Langenselbold, Germany). Multiplates: standard 96-well multiplates (Diagonal, Muenster, Germany). Shaker: self-made device with adjustable temperature and tumbling speed (scientific workshop of the institute). Vortexer: Vortex Genie 2 (Thermo Fisher Scientific, Langenselbold, Germany). Harvester: MicroBeta FilterMate-96 Harvester. Filter: Printed Filtermat Typ A and B. Scintillator: Meltilex (Typ A or B) solid state scintillator. Scintillation analyzer: MicroBeta Trilux (all Perkin Elmer LAS, Rodgau-Jügesheim, Germany). Chemicals and reagents were purchased from different commercial sources and of analytical grade.

Preparation of membrane homogenates from guinea pig brain[1-3]

Five guinea pig brains were homogenized with the potter (500-800 rpm, 10 up-and-down strokes) in 6 volumes of cold 0.32 M sucrose. The suspension was centrifuged at 1200 x *g* for 10 min at 4 °C. The supernatant was separated and centrifuged at 23500 x *g* for 20 min at 4 °C. The pellet was resuspended in 5-6 volumes of buffer (50 mM TRIS, pH 7.4) and centrifuged again at 23500 x g (20 min, 4 °C). This procedure was repeated twice. The final pellet was resuspended in 5-6 volumes of buffer and frozen (−80 °C) in 1.5 mL portions containing about 1.5 mg protein/mL.

Preparation of membrane homogenates from rat brain[4-6]

5 rat brains (Sprague Dawley rats) were homogenized with the potter (500-800 rpm, 10 up-and-down strokes) in 6 volumes of cold 0.32 M sucrose. The suspension was centrifuged at 1,200 x *g* for 10 min at 4 °C. The supernatant was separated and centrifuged at 23,500 x *g* for 20 min at 4 °C. The pellet was resuspended in 5-6 volumes

of buffer (50 mM TRIS, pH 7.4) and centrifuged again at 23,500 x *g* (20 min, 4 °C). This procedure was repeated twice. The final pellet was resuspended in 5-6 volumes of buffer and stored at -80,°C in 1.5 mL portions containing about 1.5 mg protein/mL.

Preparation of membrane homogenates from rat liver[1-3]

Two rat livers were cut into small pieces and homogenized with the potter (500-800 rpm, 10 up-and-down strokes) in 6 volumes of cold 0.32 M sucrose. The suspension was centrifuged at 1,200 x *g* for 10 min at 4 °C. The supernatant was separated and centrifuged at 31,000 x *g* for 20 min at 4 °C. The pellet was resuspended in 5-6 volumes of buffer (50 mM TRIS, pH 8.0) and incubated at room temperature for 30 min. After the incubation, the suspension was centrifuged again at 31,000 x *g* for 20 min at 4 °C. The final pellet was resuspended in 5-6 volumes of buffer and stored at -80,°C in 1.5 mL portions containing about 2 mg protein/mL.

Protein determination

The protein concentration was determined by the method of Bradford,[7] modified by Stoscheck.[8] The Bradford solution was prepared by dissolving 5 mg of Coomassie Brilliant Blue G 250 in 2.5 mL of EtOH (95%, v/v). 10 mL deionized H_2O and 5 mL phosphoric acid (85%, m/v) were added to this solution, the mixture was stirred and filled to a total volume of 50.0 mL with deionized H_2O. The calibration was carried out using bovine serum albumin as a standard in 9 concentrations (0.1, 0.2, 0.4, 0.6, 0.8, 1.0, 1.5, 2.0 and 4.0 mg/mL). In a 96-well standard multiplate, 10 µL of the calibration solution or 10 µL of the membrane receptor preparation were mixed with 190 µL of the Bradford solution, respectively. After 5 min, the UV absorption of the protein-dye complex at λ = 595 nm was measured with a platereader (Tecan Genios, Tecan, Crailsheim, Germany).

General procedures for the binding assays

The test compound solutions were prepared by dissolving approximately 10 µmol (usually 2-4 mg) of test compound in DMSO so that a 10 mM stock solution was obtained. To obtain the required test solutions for the assay, the DMSO stock solution was diluted with the respective assay buffer. The filtermats were presoaked in 0.5 % aqueous

polyethylenimine solution for 2 h at room temperature before use. All binding experiments were carried out in duplicates in the 96-well multiplates. The concentrations given are the final concentration in the assay. Generally, the assays were performed by addition of 50 µL of the respective assay buffer, 50 µL of test compound solution in various concentrations (10^{-5}, 10^{-6}, 10^{-7}, 10^{-8}, 10^{-9} and 10^{-10} mol/L), 50 µL of corresponding radioligand solution and 50 µL of the respective receptor preparation into each well of the multiplate (total volume 200 µL). The receptor preparation was always added last. During the incubation, the multiplates were shaken at a speed of 500-600 rpm at the specified temperature. Unless otherwise noted, the assays were terminated after 120 min by rapid filtration using the harvester. During the filtration each well was washed five times with 300 µL of water. Subsequently, the filtermats were dried at 95 °C. The solid scintillator was melted on the dried filtermats at a temperature of 95 °C for 5 minutes. After solidifying of the scintillator at room temperature, the trapped radioactivity in the filtermats was measured with the scintillation analyzer. Each position on the filtermat corresponding to one well of the multiplate was measured for 5 min with the [^{3}H]tritium counting protocol. The overall counting efficiency was 20 %. The IC_{50}-values were calculated with the program GraphPad Prism® 3.0 (GraphPad Software, San Diego, CA, USA) by non-linear regression analysis. Subsequently, the IC_{50} values were transformed into K_i-values using the equation of Cheng and Prusoff.[9] The K_i-values are given as mean value ± SEM from three independent experiments.

Determination of MOR affinity (guinea pig brain)[4-6]

The assay was performed with the radioligand [^{3}H]DAMGO (51 Ci/mmol, Perkin Elmer LAS). The thawed guinea pig brain membrane preparation (about 100 µg of the protein) was incubated with various concentrations of test compounds, 3 nM [^{3}H]DAMGO, and TRIS-$MgCl_2$-Puffer (50 mM, 8 mM $MgCl_2$, pH 7.4) at 37 °C. The non-specific binding was determined with 10 µM unlabeled Naloxon. The K_d-value of DAMGO is 0.57 nM.

Determination of DOR affinity (rat brain)[4-6]

The assay was performed with the radioligand [^{3}H]DPDPE (69 Ci/mmol, Amersham). The thawed rat brain membrane preparation (about 75 µg of the protein) was incubated with

various concentrations of test compounds, 3 nM [^{3}H]DPDPE, and TRIS-$MgCl_2$-PMSF-buffer (50 mM, 8 mM $MgCl_2$, 400 µM PMSF, pH 7.4) at 37 °C. The non-specific binding was determined with 10 µM unlabeled Morphine. The K_d-value of DPDPE is 0.65 nM.

Determination of the σ_1 receptor affinity (guinea pig brain)[1-3]

The assay was performed with the radioligand (#)-[^{3}H]pentazocine (22.0 Ci/mmol; Perkin Elmer). The thawed membrane preparation of guinea pig brains (about 100 µg of the protein) was incubated with various concentrations of test compounds, 2 nM (+)-[^{3}H]Pentazocine, and TRIS buffer (50 mM, pH 7.4) at 37 °C. The non-specific binding was determined with 10 µM unlabeled (+)-pentazocine. The K_d-value of (+)-pentazocine is 2.9 nM.[10]

Determination of the σ_2 receptor affinity (rat liver)[1-3]

The assays were performed with the radioligand [^{3}H]di-*o*-tolylguanidine ([^{3}H]DTG, specific activity 50 Ci/mmol; ARC, St. Louis, MO, USA). The thawed membrane preparation of rat liver containing 100 µg protein was incubated with various concentrations of the test compound, 3 nM [^{3}H]DTG and buffer containing (+)-pentazocine (500 nM (+)-pentazocine in 50 mM TRIS, pH 8.0) at room temperature. The non-specific binding was determined with 10 µM non-labeled DTG. The K_d values is 17.9 nM.[11]

2. Quality control of [^{18}F]2 and its stability in blood serum

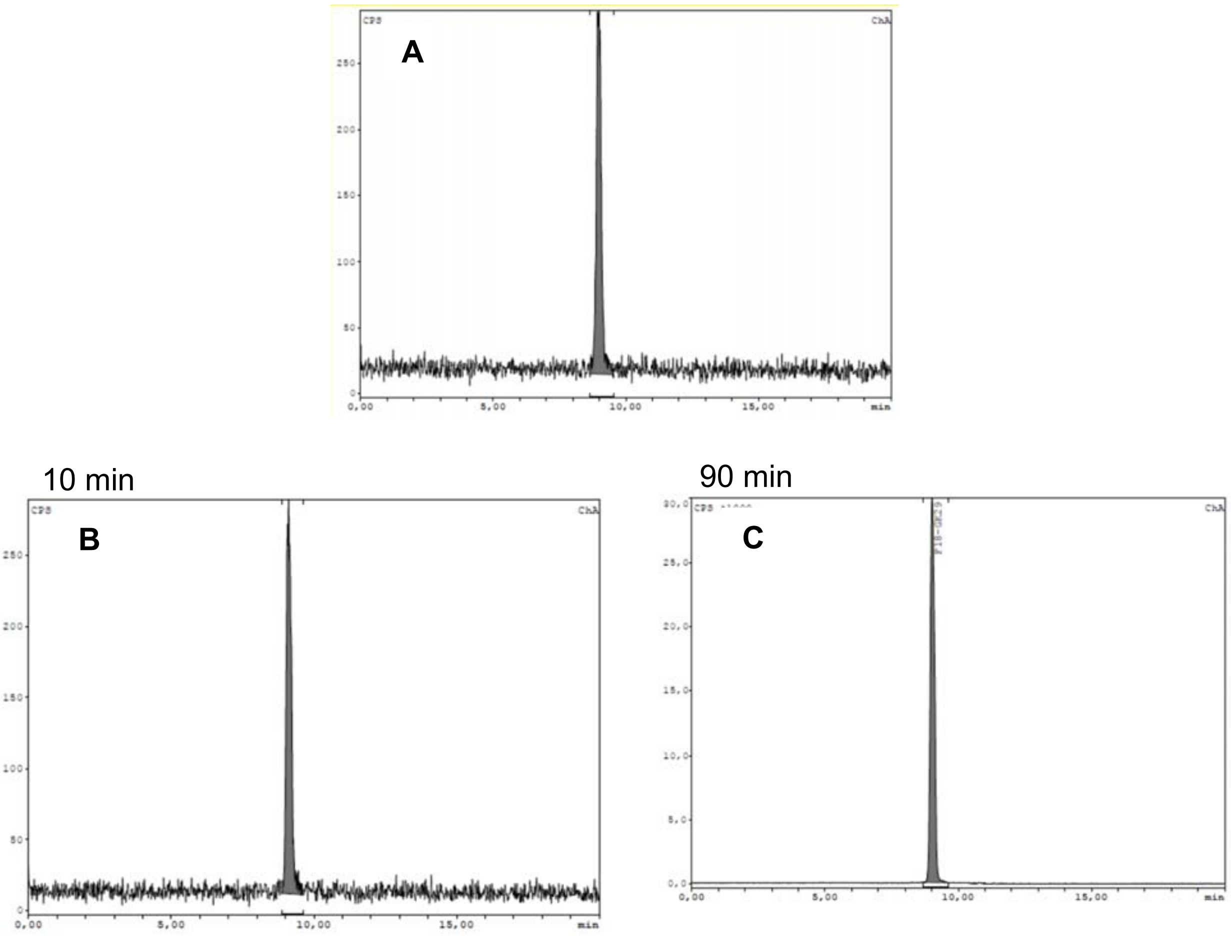

Figure S1: Radio-HPLC chromatograms of a typical quality control (QC) of produced [^{18}F]**2** (**A**) and of *in vitro* stability of [^{18}F]**2** after incubation with mouse blood serum at 37 °C for 10 min (**B**) and 90 min (**C**).

3. Investigation of *in vivo* radiometabolites of [^{19}F]2

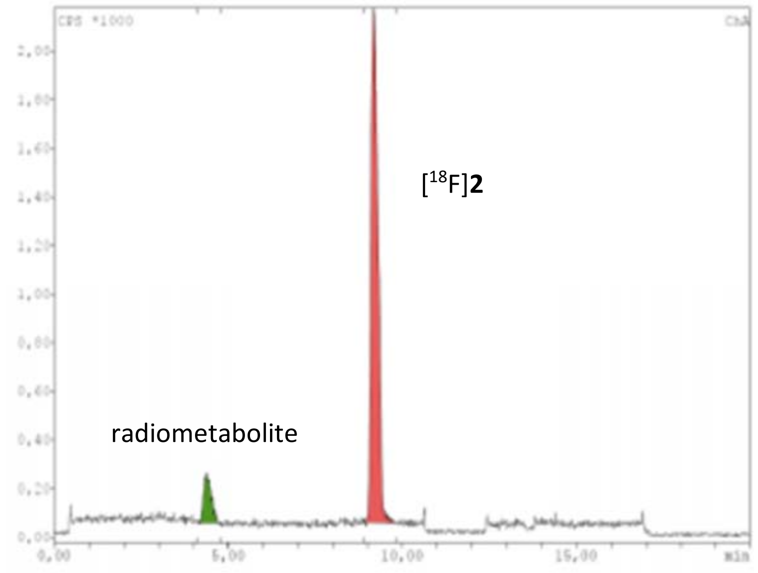

Figure S2: Radio-HPLC chromatogram of mice plasma 90 min after injection of [^{18}F]**2**.

4. Distribution of the PET tracer [^{18}F]14c in various organs

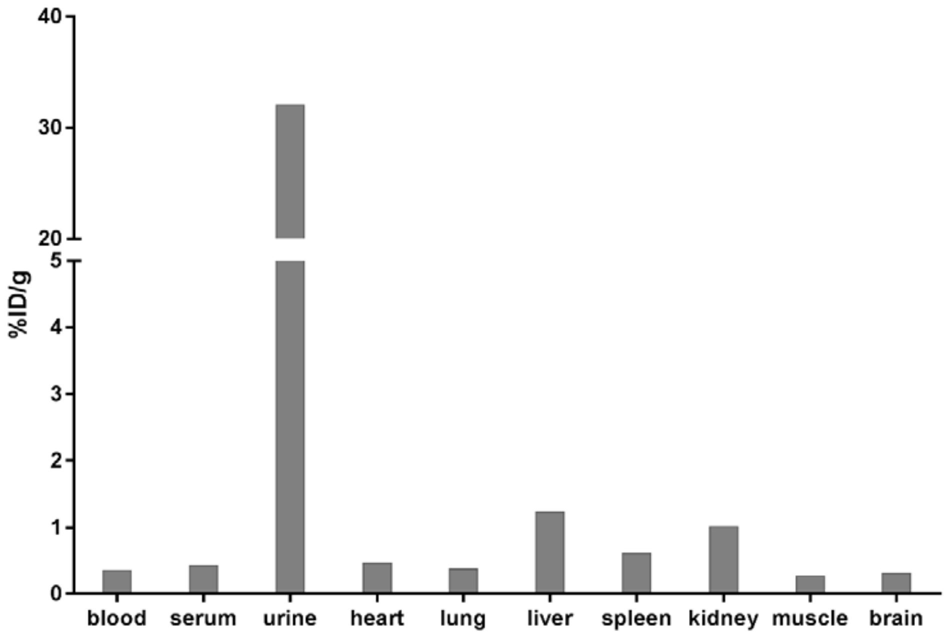

Figure S3: *Ex vivo* biodistribution of radiotracer [^{18}F]**14c** determined by scintillation counting 3 - 4 h p. i..

5. *in vitro* biotransformation of 14c in the presence of mouse liver microsomes

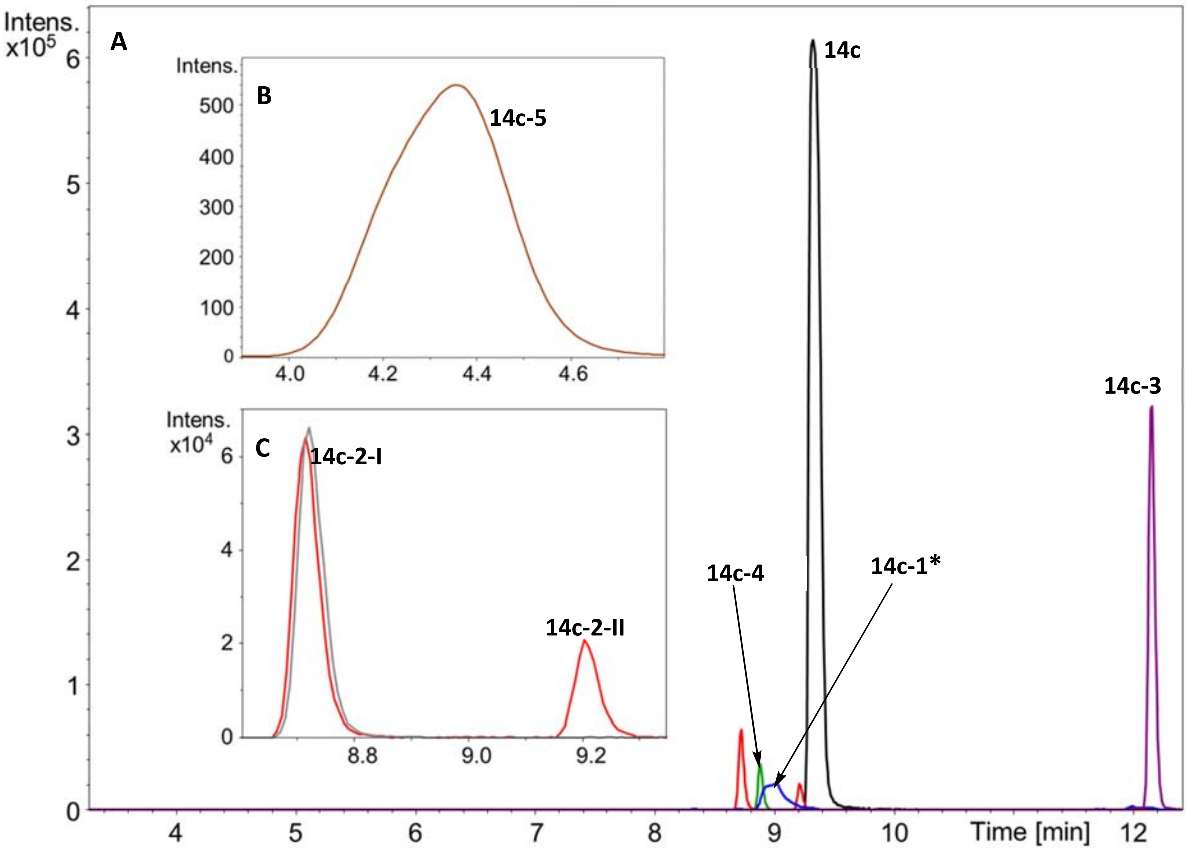

Figure S4: EICs (m/z ± 0.01, ESI positive mode, $[M+H]^+$) for **A**: parent compound **14c** (m/z 472.1565, black) and its metabolites **14c-1** (m/z 470.1408, blue), **14c-4** (m/z 400.1189, green) and **14c-3** (m/z 450.1346, purple); **B**: metabolite **14c-5** (m/z 414.1510, brown); **C**: both isomers of metabolite **14c-2** (m/z 452.1502) after incubation with mouse liver microsome preparations and NADPH (red) and negative control (grey); LC-qToF setup, method LC-MS.

*For depiction of the EIC of metabolite **14c-1** the m/z of the in-source fragment $[M-H_2O+H]^+$ is used, as it revealed higher intensity.

6. Identification of 14c and its metabolites by LC-MS

Table S1: Identification of parent compound **14c**, LC-MS method E, t_R = 9.3 min, width: m/z ± 5, CE: 35 eV. *Mixture of two diastereomers.

obs. m/z	calc. m/z	sum formula
472.1557	472.1565	$C_{22}H_{29}Cl_2FN_3O_3^+$
383.0919	383.0924	$C_{18}H_{21}Cl_2N_2O_3^+$
351.0649	351.0662	$C_{17}H_{17}Cl_2N_2O_2^+$
315.0282	315.0298	$C_{13}H_{13}Cl_2N_2O_3^+$
287.0339	287.0349	$C_{12}H_{13}Cl_2N_2O_2^+$
273.0185	273.0192	$C_{11}H_{11}Cl_2N_2O_2^+$
230.0124	230.0134	$C_{10}H_{10}Cl_2NO^+$
197.1281	197.1285	$C_{10}H_{17}N_2O_2^+$
165.1016	165.1022	$C_9H_{13}N_2O^+$
158.9749	158.9763	$C_7H_5Cl_2^+$

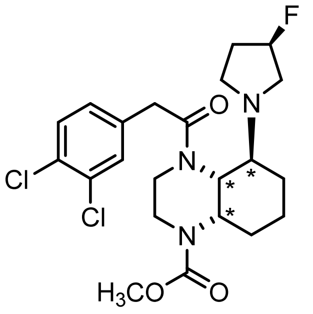

14c*

Table S2: Identification of metabolite **14c-1**, LC-MS method E, t_R = 9.0 min, width: m/z ± 5, CE: 35 eV.

obs. m/z	calc. m/z	sum formula
488.1512	488.1514	$C_{22}H_{29}Cl_2FN_3O_4^+$
383.0927	383.0924	$C_{18}H_{21}Cl_2N_2O_3^+$
197.1295	197.1285	$C_{10}H_{17}N_2O_2^+$

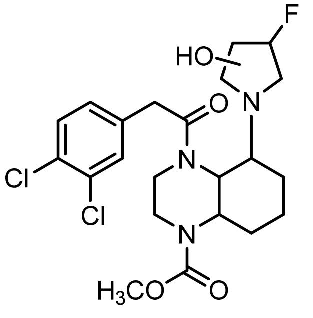

14c-1

Table S3: Identification of impurity **14c-2-I**, LC-MS method E, t_R = 8.7 min, width: m/z ± 5, CE: 35 eV.

obs. m/z	calc. m/z	sum formula
452.1512	452.1502	$C_{22}H_{28}Cl_2N_3O_3^+$
383.0900	383.0924	$C_{18}H_{21}Cl_2N_2O_3^+$
266.1846	266.1863	$C_{14}H_{24}N_3O_2^+$
230.0126	230.0134	$C_{10}H_{10}Cl_2NO^+$
197.1266	197.1285	$C_{10}H_{17}N_2O_2^+$
165.1022	165.1022	$C_9H_{13}N_2O^+$

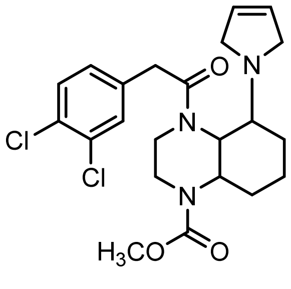

14c-2-I

Table S4: Identification of metabolite **14c-2-II**, LC-MS method E, t_R = 9.2 min, width: m/z ± 5, CE: 35 eV.

obs. m/z	calc. m/z	sum formula
452.1510	452.1502	$C_{22}H_{28}Cl_2N_3O_3^+$
383.0902	383.0924	$C_{18}H_{21}Cl_2N_2O_3^+$
266.1879	266.1863	$C_{14}H_{24}N_3O_2^+$
230.0134	230.0134	$C_{10}H_{10}Cl_2NO^+$
197.1268	197.1285	$C_{10}H_{17}N_2O_2^+$
165.1026	165.1022	$C_9H_{13}N_2O^+$

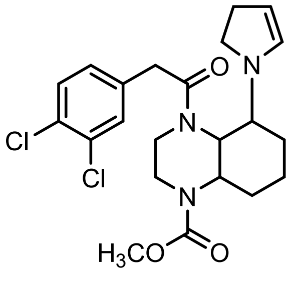

14c-2-II

Table S5: Identification of metabolite **14c-3**, LC-MS method E, t_R = 12.2 min, width: m/z ± 5, CE: 35 eV.

obs. m/z	calc. m/z	sum formula
450.1334	450.1346	$C_{22}H_{26}Cl_2N_3O_3^+$
383.0920	383.0924	$C_{18}H_{21}Cl_2N_2O_3^+$
264.1697	264.1707	$C_{14}H_{22}N_3O_2^+$
230.0131	230.0134	$C_{10}H_{10}Cl_2NO^+$
197.1282	197.1285	$C_{10}H_{17}N_2O_2^+$
165.1018	165.1022	$C_9H_{13}N_2O^+$

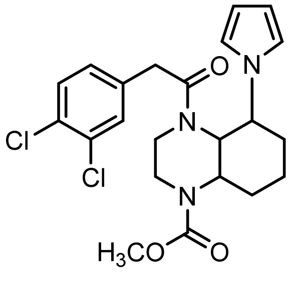

14c-3

Table S6: Identification of metabolite **14c-4**, LC-MS method E, t_R = 8.9 min.

obs. m/z	calc. m/z	sum formula
400.1166	400.1189	$C_{18}H_{24}Cl_2N_3O_3^+$

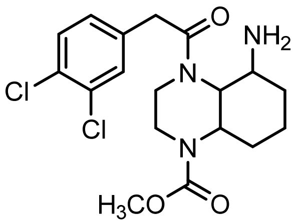

14c-4

Table S7: Identification of metabolite **b14c-5**, LC-MS method E, t_R = 4.3 min.

obs. m/z	calc. m/z	sum formula
414.1517	414.1510	$C_{20}H_{27}Cl_2FN_3O^+$

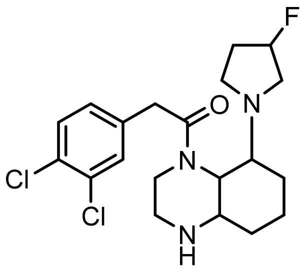

14c-5

7. References

1. Hasebein, P.; Frehland, B.; Lehmkuhl, K.; Fröhlich, R.; Schepmann, D.; Wünsch, B. Synthesis and pharmacological evaluation of like- and unlike-configured tetrahydro-2-benzazepines with the α-substituted benzyl moiety in the 5-position. *Org. Biomol. Chem.* **2014**, *12* (29), 5407–5426.
2. Meyer, C.; Neue, B.; Schepmann, D.; Yanagisawa, S.; Yamaguchi, J.; Würthwein, E.-U.; Itami, K.; Wünsch, B. Improvement of σ1 receptor affinity by late-stage C-H-bond arylation of spirocyclic lactones. *Bioorg. Med. Chem.* **2013**, *21* (7), 1844–1856.
3. Miyata, K.; Schepmann, D.; Wünsch, B. Synthesis and σ receptor affinity of regioisomeric spirocyclic furopyridines. *Eur. J. Med. Chem.* **2014***, 83*, 709–716.
4. Wittig, C.; Schepmann, D.; Soeberdt, M.; Daniliuc, C. G.; Wünsch, B. Stereoselective Synthesis of Conformationally Restricted KOR Agonists Based on the 2,5-diazabicyclo[2.2.2]octane Scaffold. *Org. Biomol. Chem.* **2017**, *15* (31), 6520–6540.

5, Geiger, C.; Zelenka, C.; Lehmkuhl, K.; Schepmann, D.; Englberger, W.; Wünsch, B. Conformationally Constrained Κ Receptor Agonists: Stereoselective Synthesis and Pharmacological Evaluation of 6,8-Diazabicyclo[3.2.2]nonane Derivatives. *J. Med. Chem* **2010**, *53*, 4212–4222.

6. Kracht, D.; Rack, E.; Schepmann, D.; Fröhlich, R.; Wünsch, B. Stereoselective Synthesis and Structure–affinity Relationships of Bicyclic κ Receptor Agonists. *Org. Biomol. Chem.* **2010**, *8(1),* 212–225.
7. Bradford, M. M. A Rapid and Sensitive Method for the Quantitation of Microgram Quantities of Protein Utilizing the Principle of Protein-Dye Binding. *Anal. Biochem.* **1976**, *72*, 248–254.
8. Stoscheck, C. M. [6] Quantitation of Protein. *Methods Enzymol.* **1990**, *182*, 50–68.
9. Yung-Chi, C.; Prusoff, W. H. Relationship between the Inhibition Constant (KI) and the Concentration of Inhibitor Which Causes 50 per Cent Inhibition (I50) of an Enzymatic Reaction. *Biochem. Pharmacol.* **1973**, *22* (23), 3099–3108.
10. DeHaven-Hudkins, D. L.; Fleissner, L. C.; Ford-Rice, F. Y. Characterization of the Binding of ([^{3}H]+)-Pentazocine to Sigma Recognition Sites in Guinea Pig Brain. *Eur. J. Pharmacol.* **1992**, *227* (4), 371–378.
11. Mach, R. H.; Smith, C. R.; Childers, S. R. Ibogaine Possesses a Selective Affinity for Sigma 2 Receptors. *Life Sci.* **1995**, *57* (4), PL57-62.

8. NMR spectra

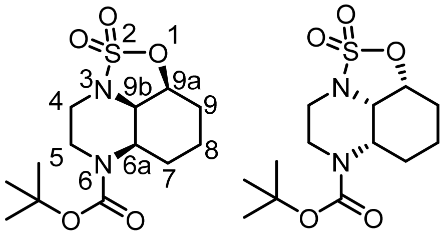

10

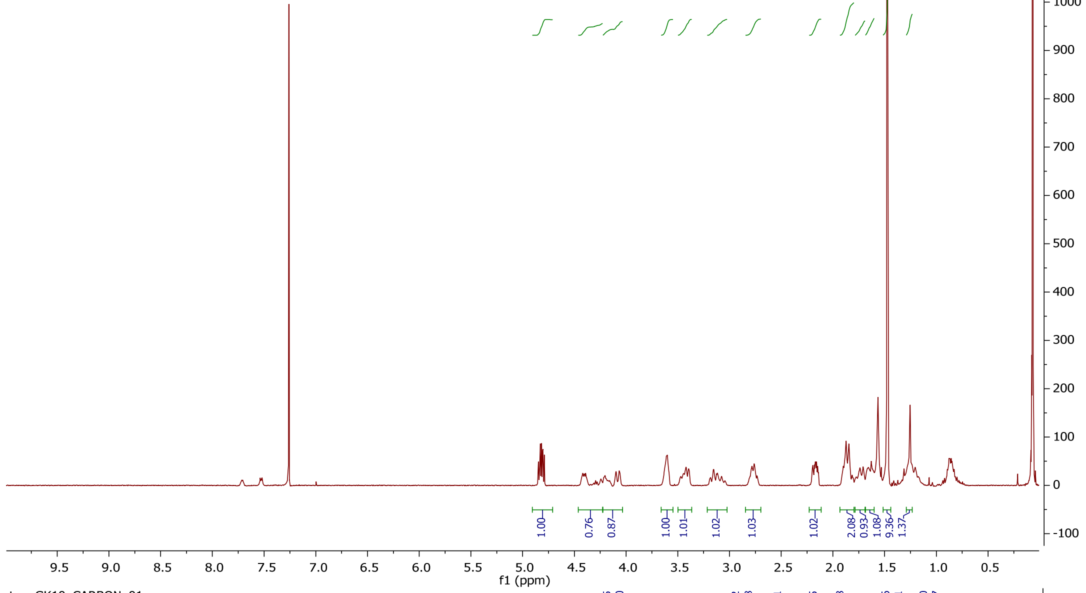

tan_GK10_CARBON_01

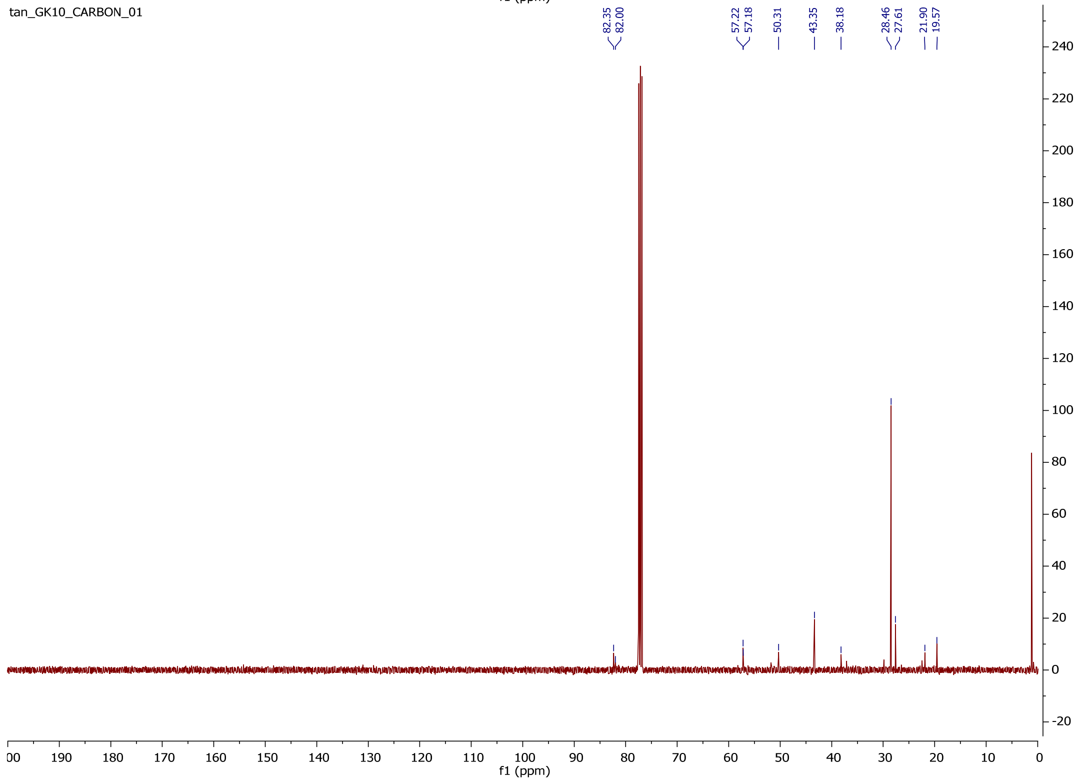

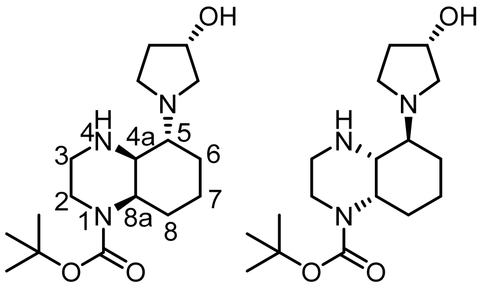
OH
N
H
4
N
4a
5
6
3
2
7
1
N
8a
8
O
O
OH
N
H
N
N
O
O

11

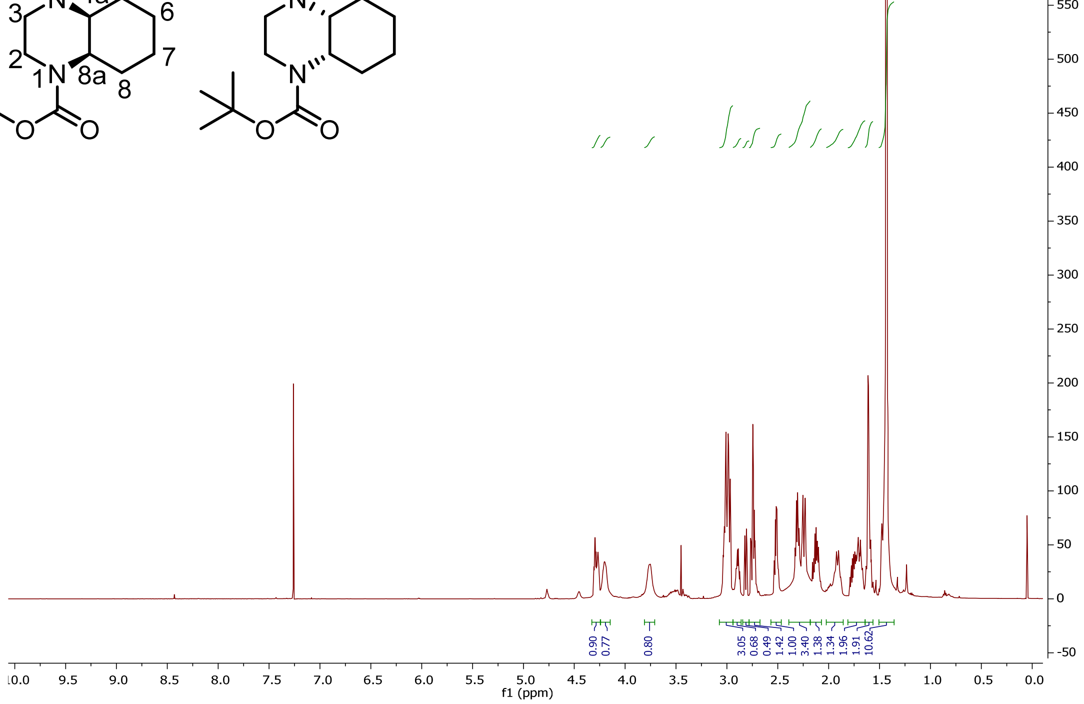
0.90
0.77
0.80
3.05
0.68
0.49
1.42
1.00
3.40
1.38
1.34
1.96
1.91
10.62
f1 (ppm)

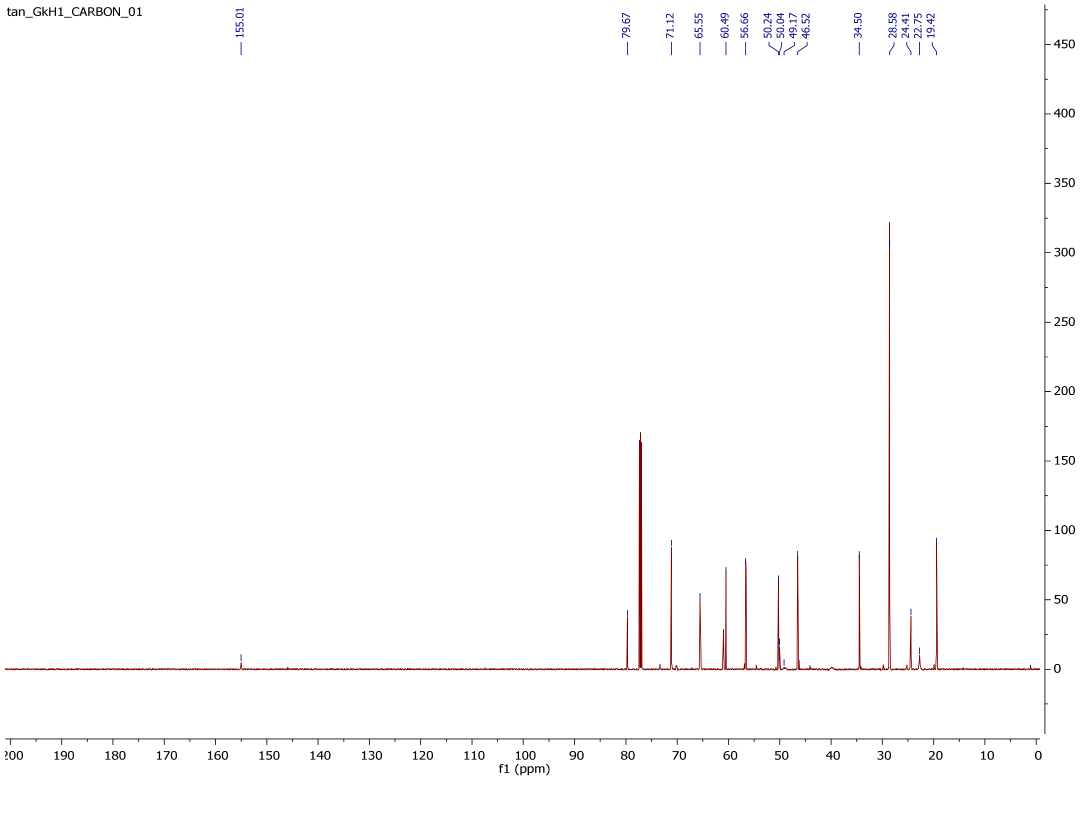
tan_GkH1_CARBON_01
155.01
79.67
71.12
65.55
60.49
56.66
50.24
50.04
49.17
46.52
34.50
28.58
24.41
22.75
19.42
f1 (ppm)

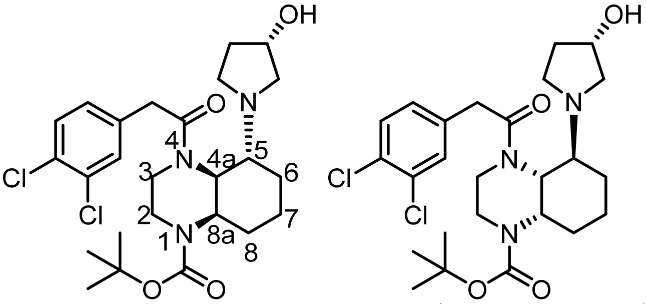

12a

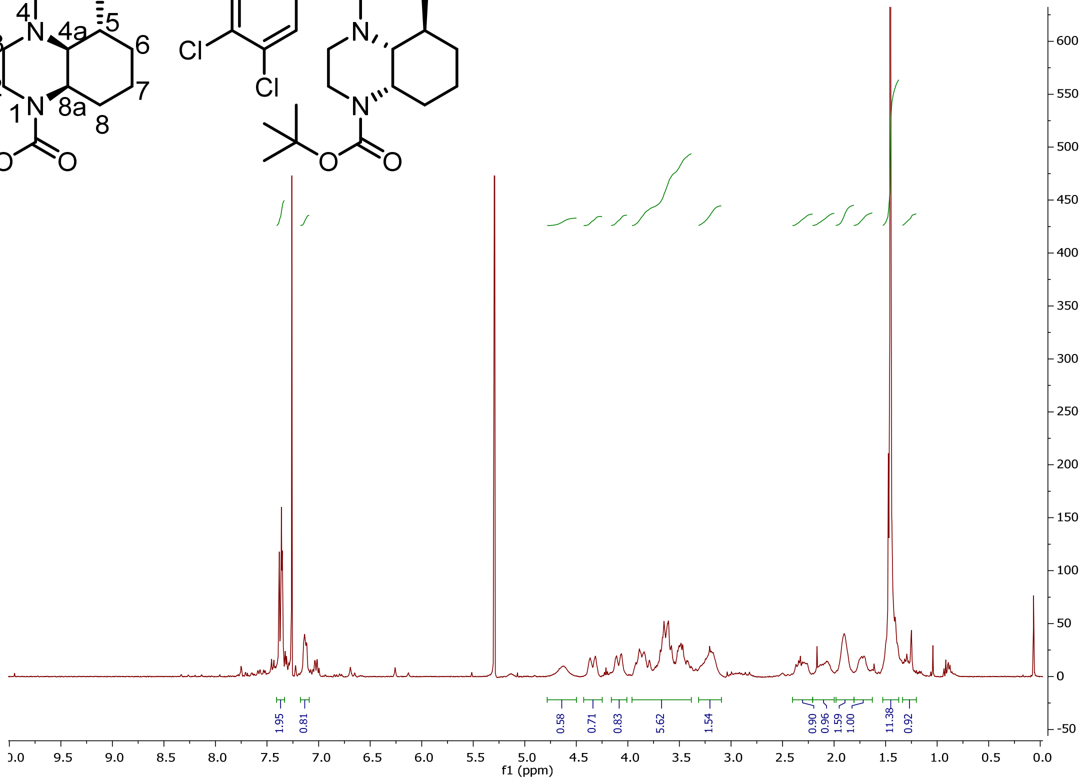
1.95
0.81
0.58
0.71
0.83
5.62
1.54
0.90
0.96
1.59
1.00
11.38
0.92
f1 (ppm)

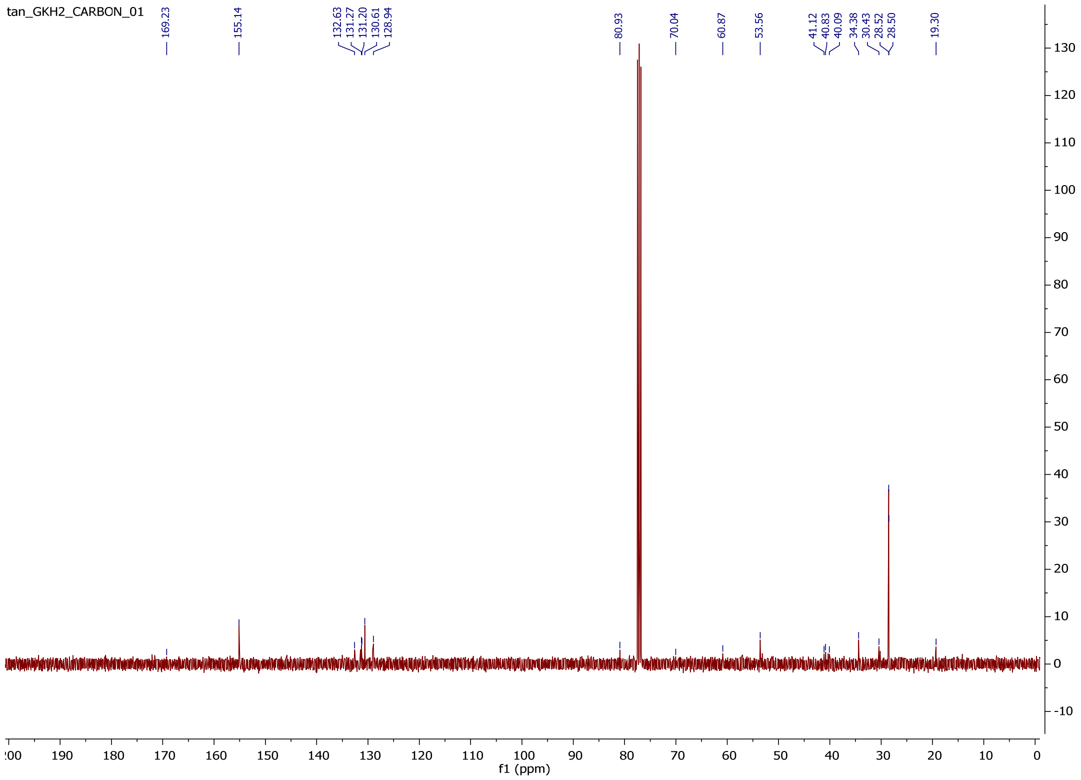
tan_GKH2_CARBON_01
169.23
155.14
132.63
131.27
131.20
130.61
128.94
80.93
70.04
60.87
53.56
41.12
40.83
40.09
34.38
30.43
28.52
28.50
19.30
f1 (ppm)

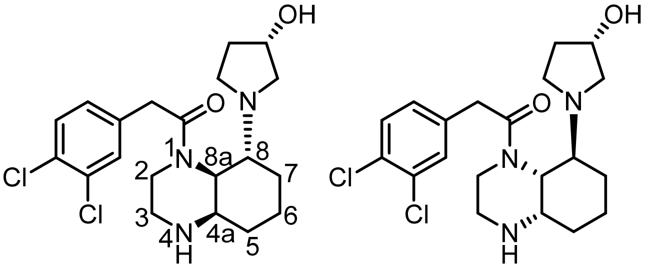
OH
O
N
Cl
Cl
1
2
3
4
N
H
8a
8
7
6
5
4a

12b

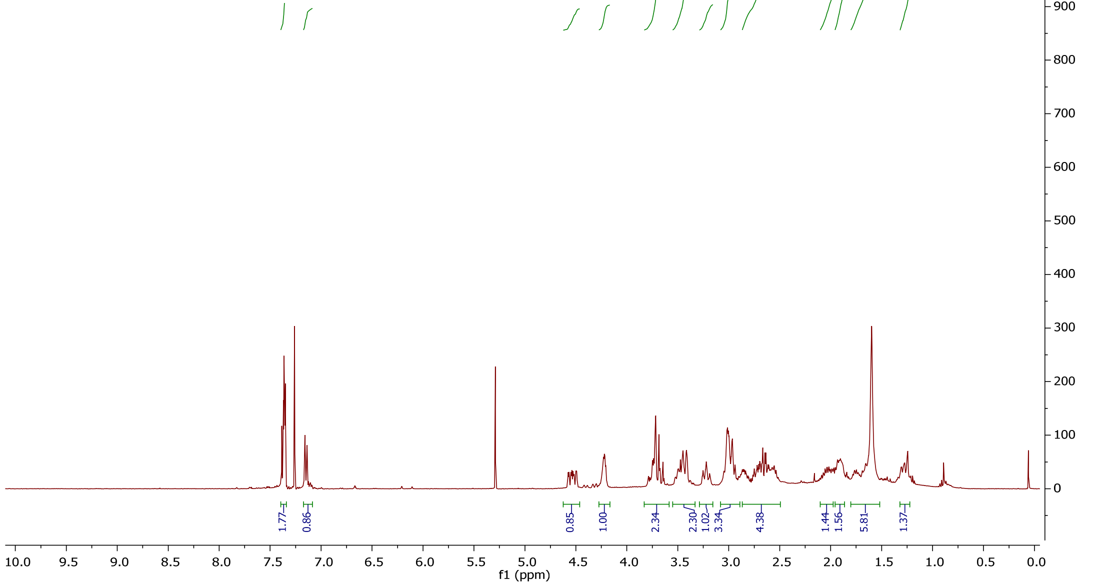
1.77
0.86
0.85
1.00
2.34
2.30
1.02
3.34
4.38
1.44
1.56
5.81
1.37
f1 (ppm)

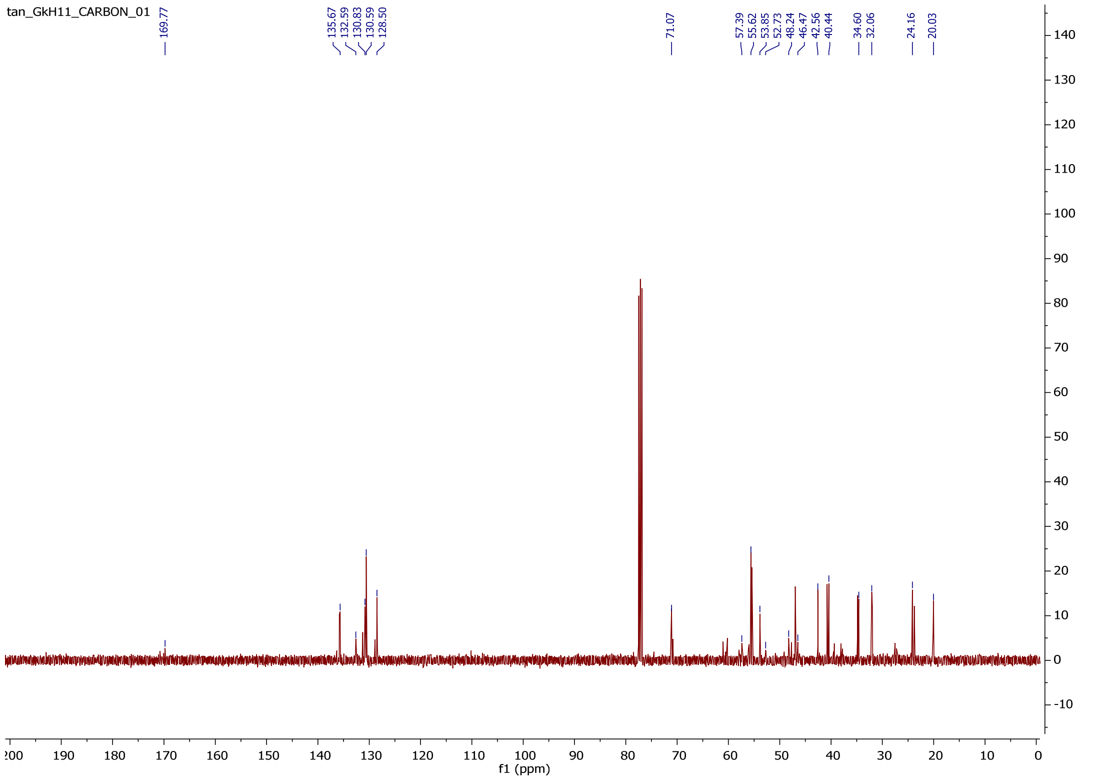
tan_GkH11_CARBON_01
169.77
135.67
132.59
130.83
130.59
128.50
71.07
57.39
55.62
53.85
52.73
48.24
46.47
42.56
40.44
34.60
32.06
24.16
20.03
f1 (ppm)

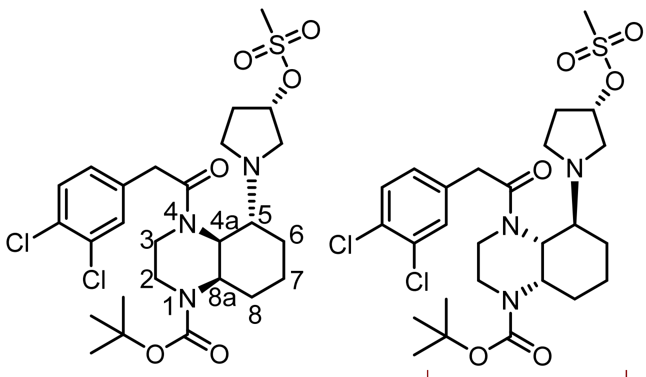
Cl
Cl
4
3
2
1
4a
5
6
7
8
8a
N
O
S
Cl
Cl

13

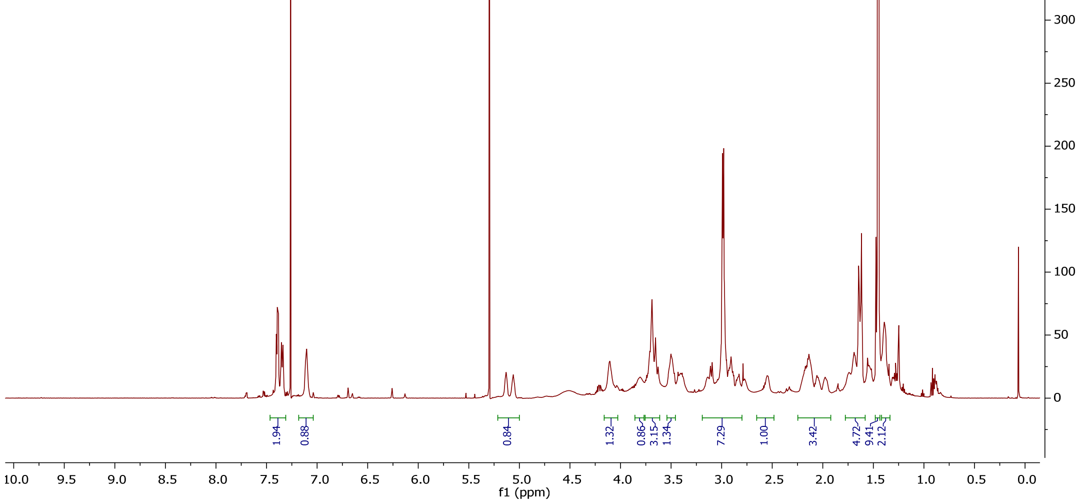
1.94
0.88
0.84
1.32
0.86
3.15
1.34
7.29
1.00
3.42
4.72
9.41
2.12
f1 (ppm)

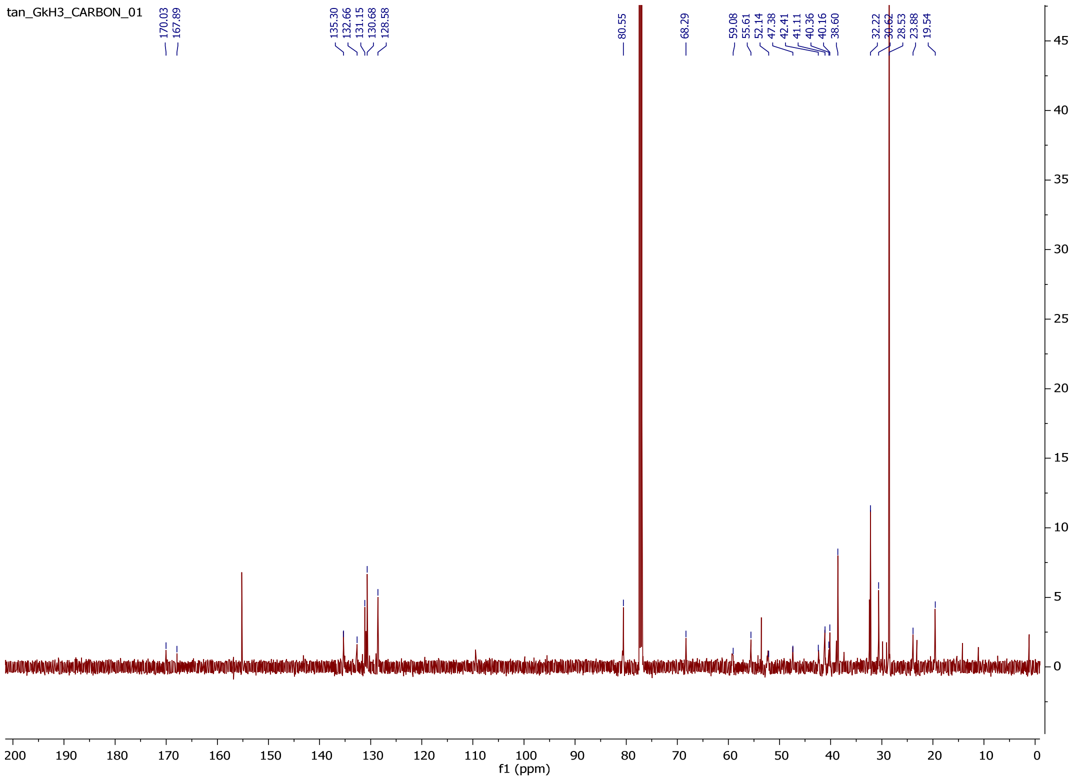
tan_GkH3_CARBON_01
170.03
167.89
135.30
132.66
131.15
130.68
128.58
80.55
68.29
59.08
55.61
52.14
47.38
42.41
41.11
40.36
40.16
38.60
32.22
28.53
23.88
19.54
f1 (ppm)

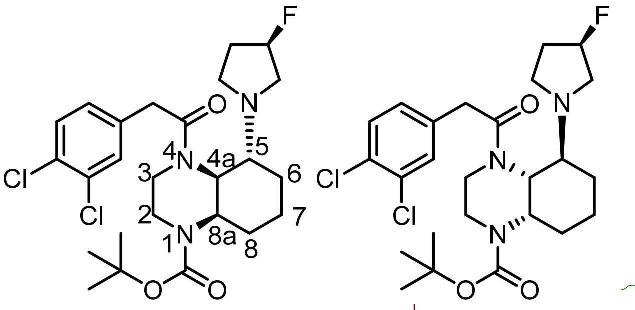

14a

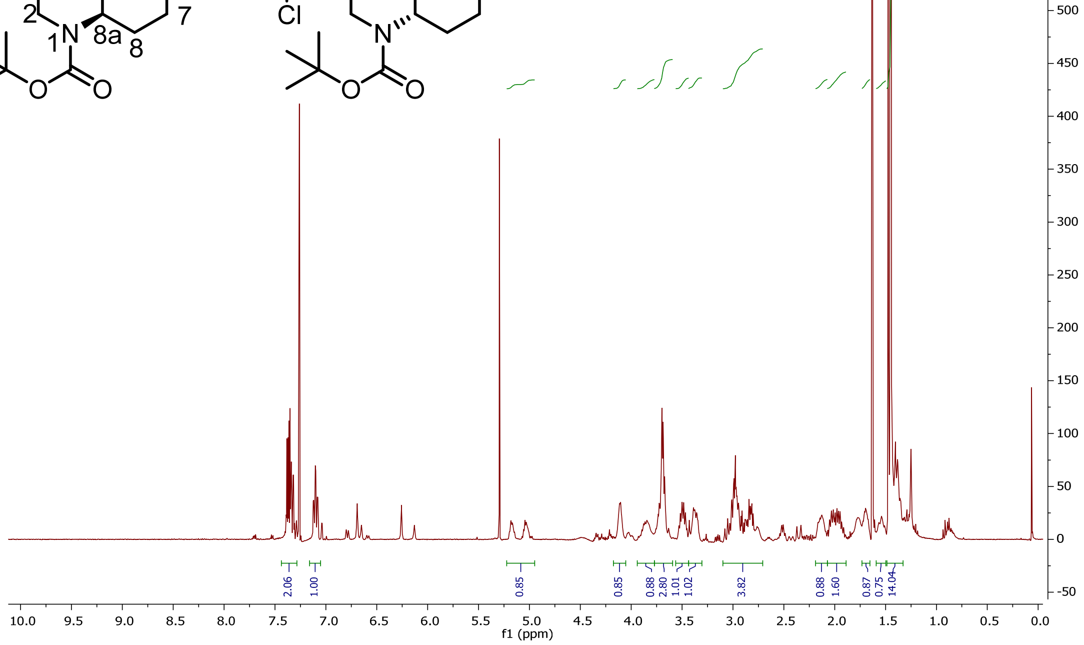
2.06
1.00
0.85
0.85
0.88
2.80
1.01
1.02
3.82
0.88
1.60
0.87
0.75
14.04
f1 (ppm)

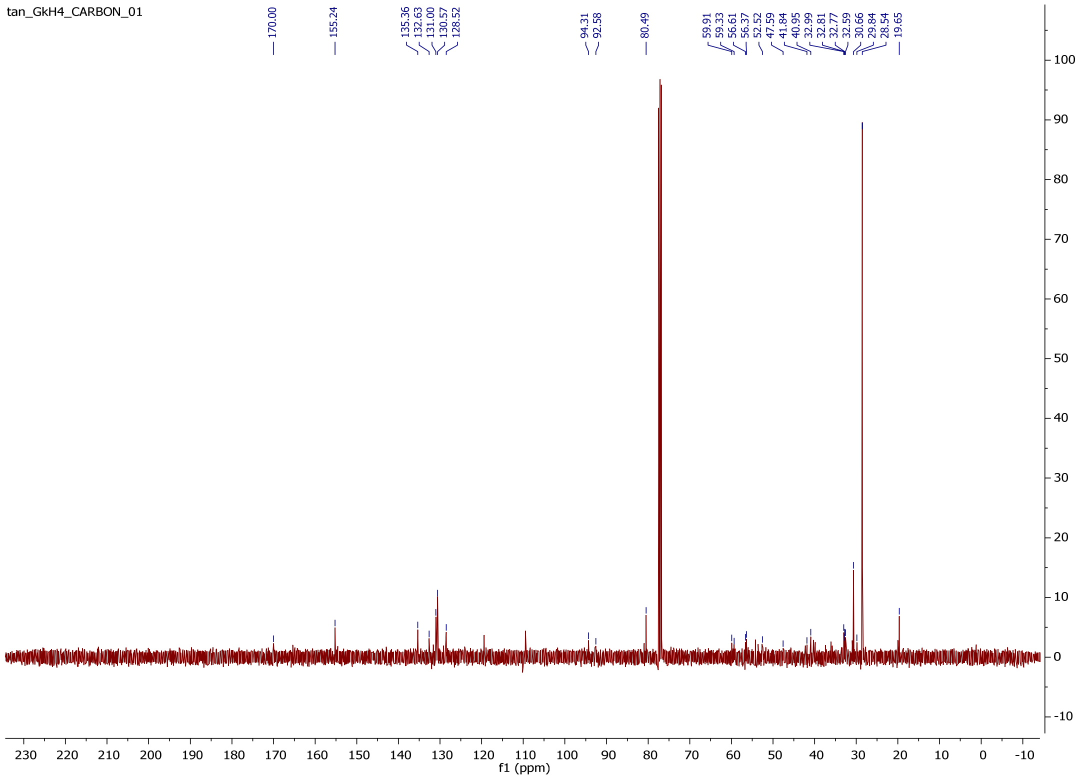
tan_GkH4_CARBON_01
170.00
155.24
135.36
132.63
131.00
130.57
128.52
94.31
92.58
80.49
59.91
59.33
56.61
56.37
52.52
47.59
41.84
40.95
32.99
32.81
32.77
32.59
30.66
29.84
28.54
19.65
f1 (ppm)

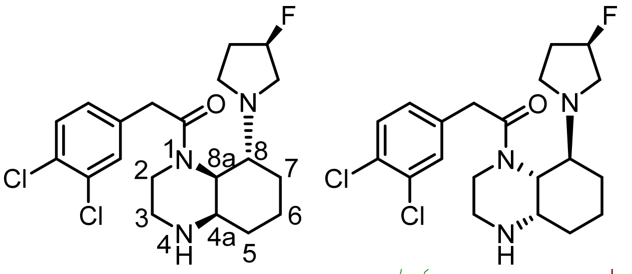

14b

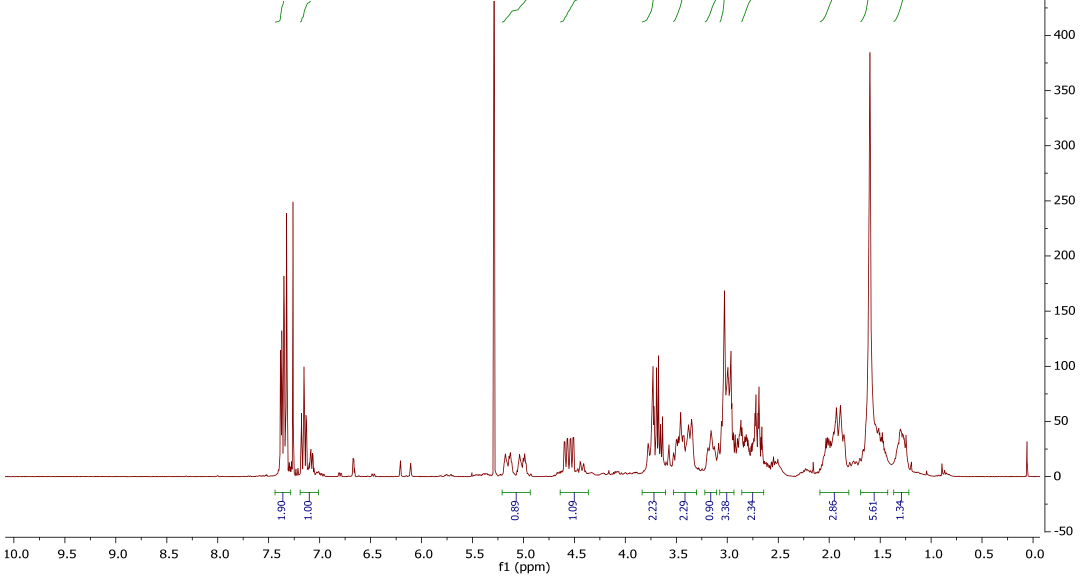

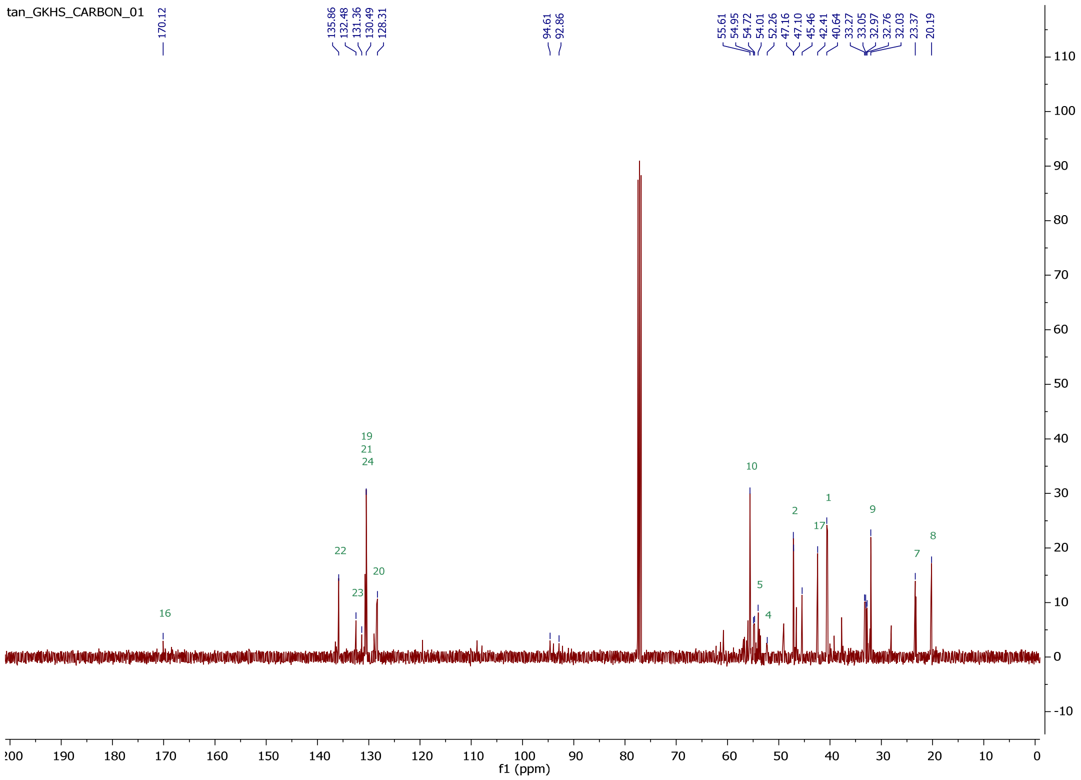

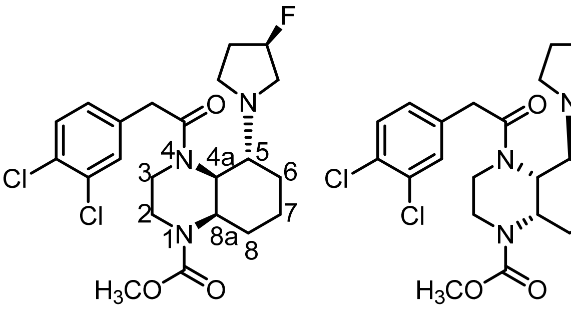
F
O
N
4
4a
5
6
3
Cl
N
7
Cl
2
1
N
8a
8
H3CO
O
F
N
O
N
Cl
Cl
N
H3CO
O

14c

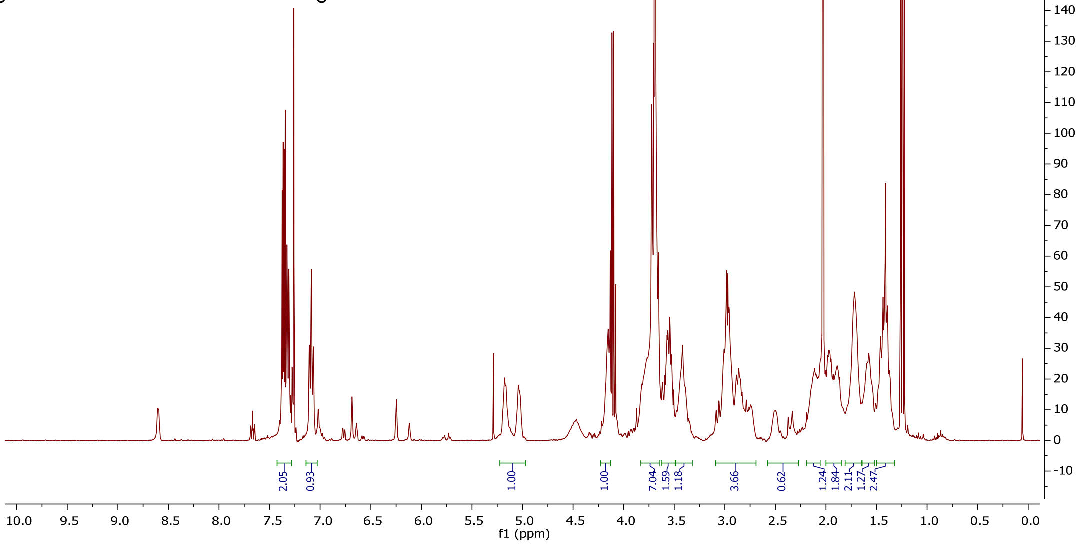
2.05
0.93
1.00
1.00
7.04
1.59
1.18
3.66
0.62
1.24
1.84
2.11
1.27
2.47
f1 (ppm)

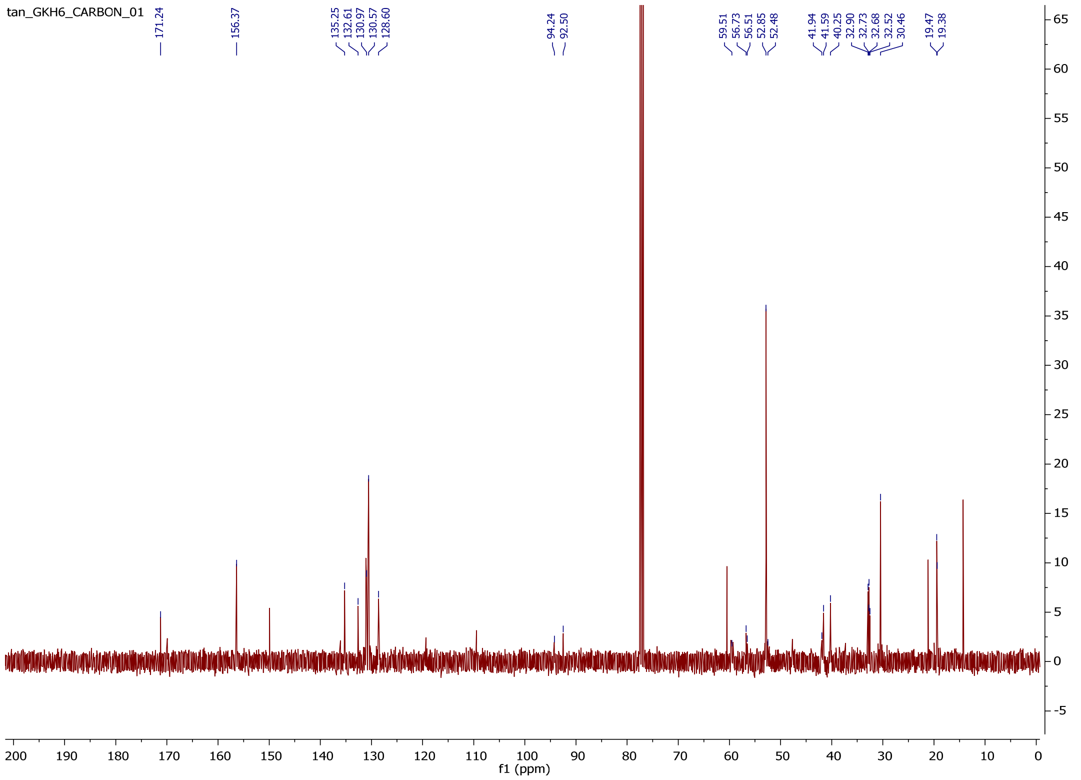
tan_GKH6_CARBON_01
171.24
156.37
135.25
132.61
130.97
130.57
128.60
94.24
92.50
59.51
56.73
56.51
52.85
52.48
41.94
41.59
40.25
32.90
32.73
32.68
32.52
30.46
19.47
19.38
f1 (ppm)

S22

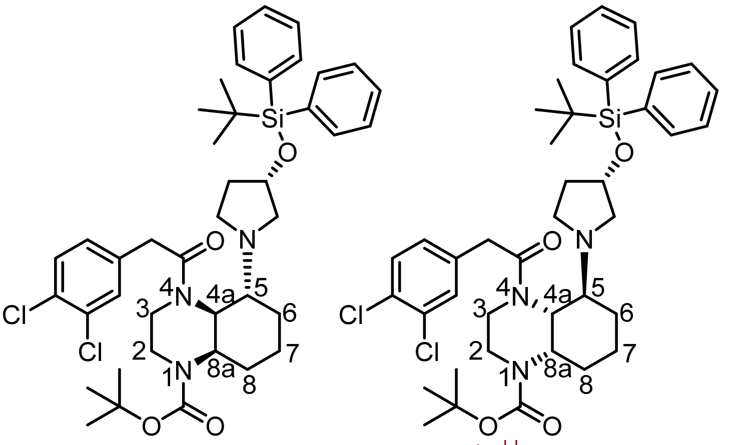

15

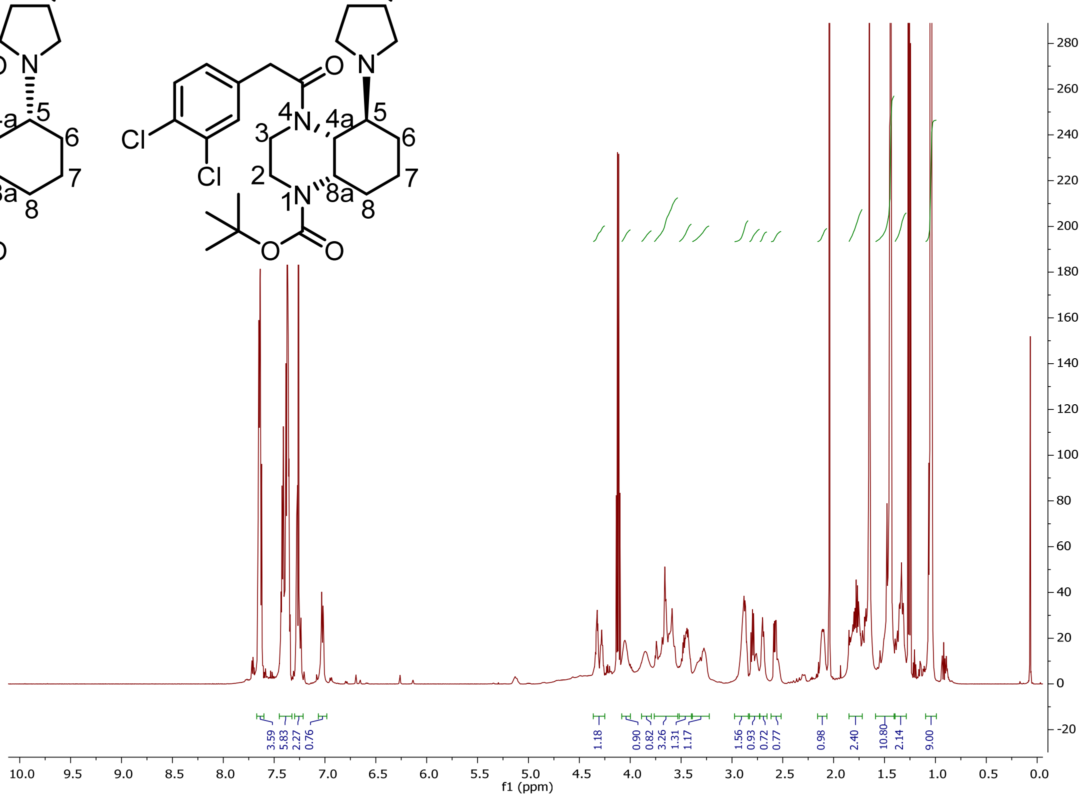
3.59
5.83
2.27
0.76
1.18
0.90
0.82
3.26
1.31
1.17
1.56
0.93
0.72
0.77
0.98
2.40
10.80
2.14
9.00
f1 (ppm)

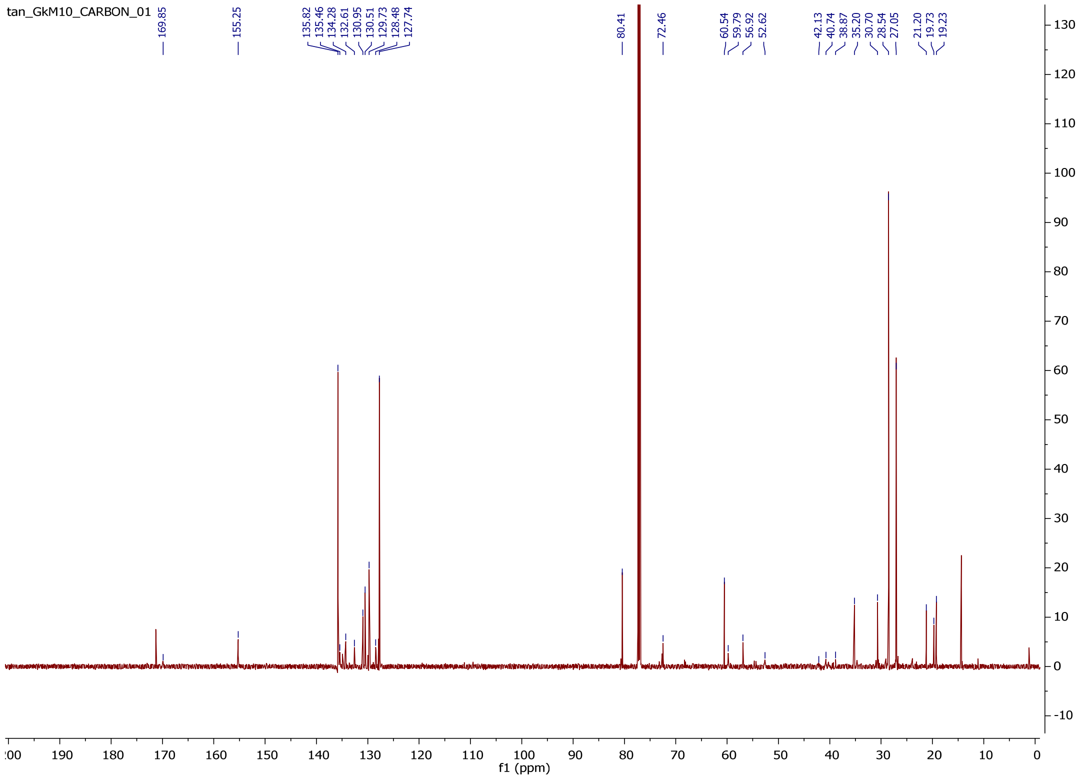
tan_GkM10_CARBON_01
169.85
155.25
135.82
135.46
134.28
132.61
130.95
130.51
129.73
128.48
127.74
80.41
72.46
60.54
59.79
56.92
52.62
42.13
40.74
38.87
35.20
30.70
28.54
27.05
21.20
19.73
19.23
f1 (ppm)

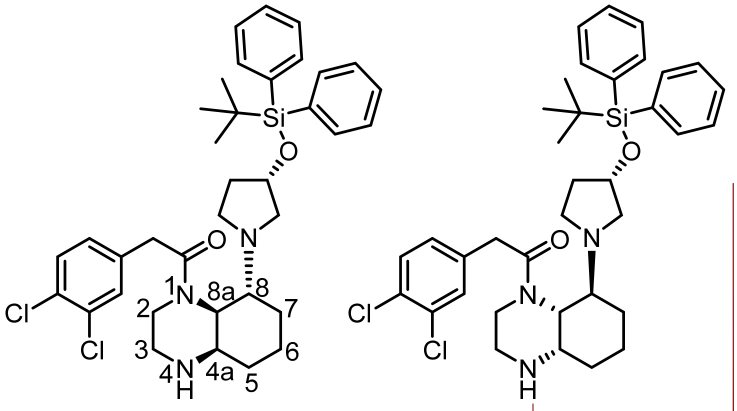
Si
O
N
O
N
Cl
Cl
1
2
3
4
4a
5
6
7
8
8a
H

16

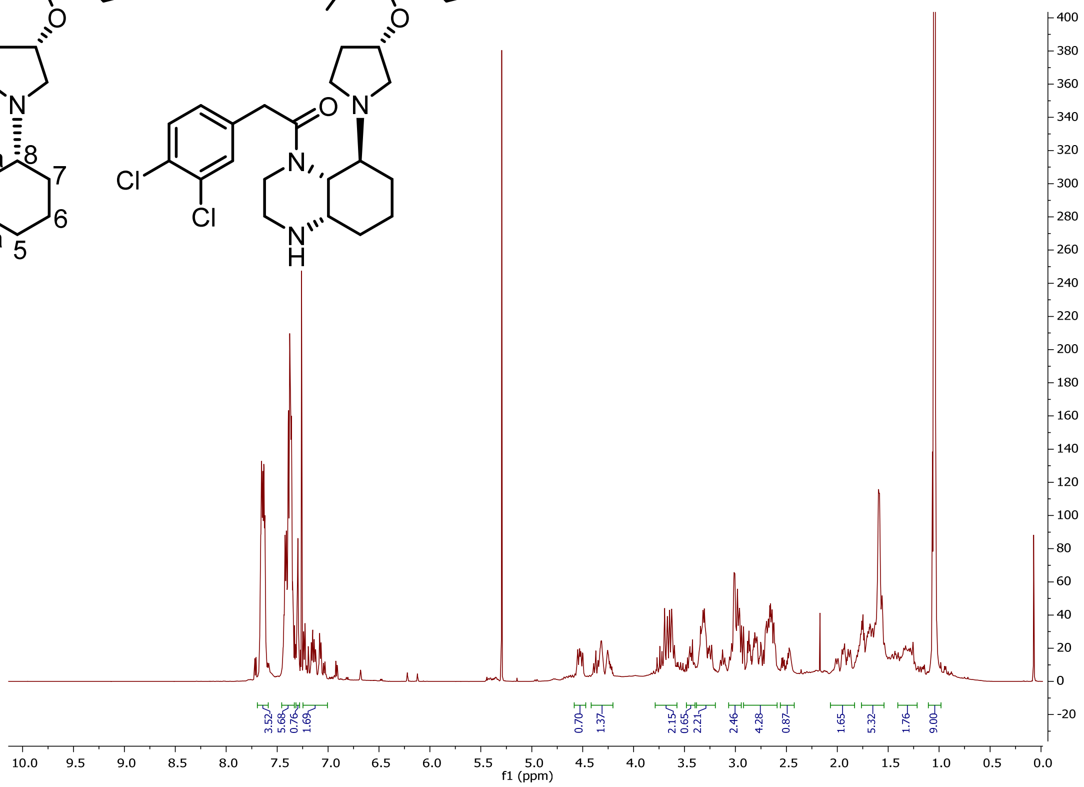
3.52
5.68
0.76
1.69
0.70
1.37
2.15
0.65
2.21
2.46
4.28
0.87
1.65
5.32
1.76
9.00
f1 (ppm)

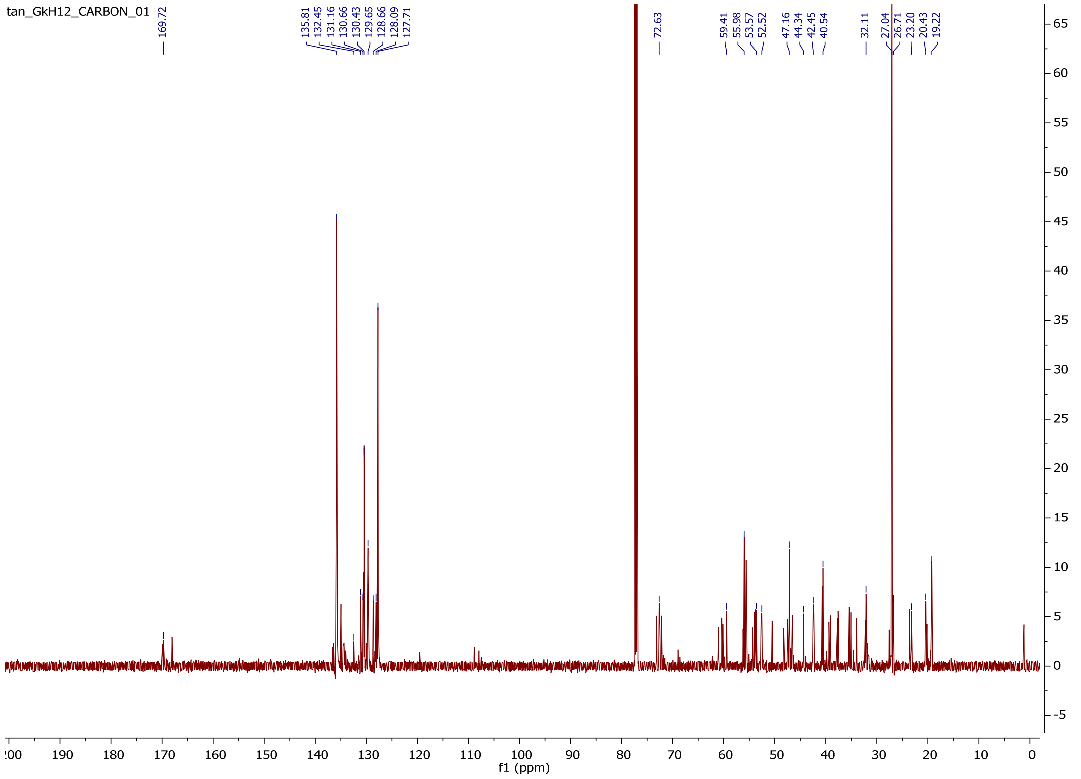
tan_GkH12_CARBON_01
169.72
135.81
132.45
131.16
130.66
130.43
129.65
128.66
128.09
127.71
72.63
59.41
55.98
53.57
52.52
47.16
44.34
42.45
40.54
32.11
27.04
26.71
23.20
20.43
19.22
f1 (ppm)

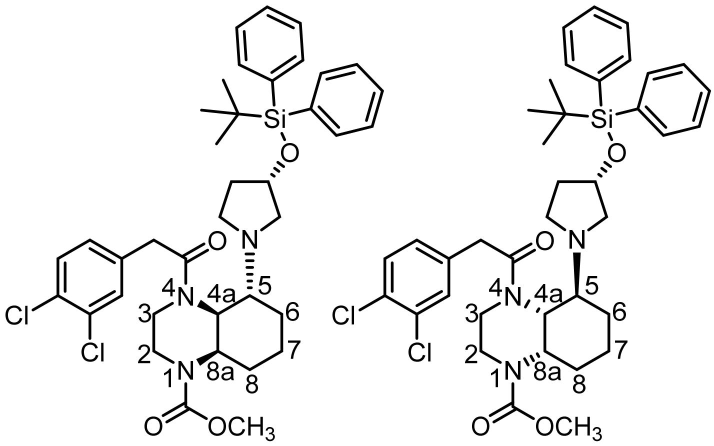

17

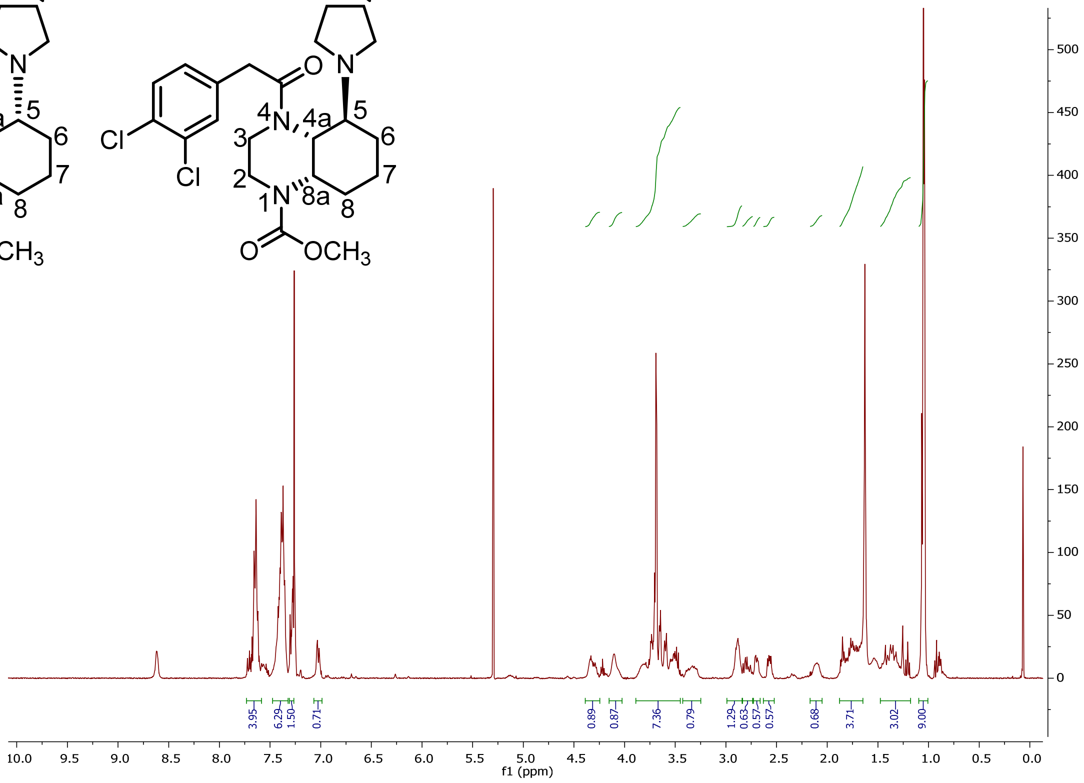

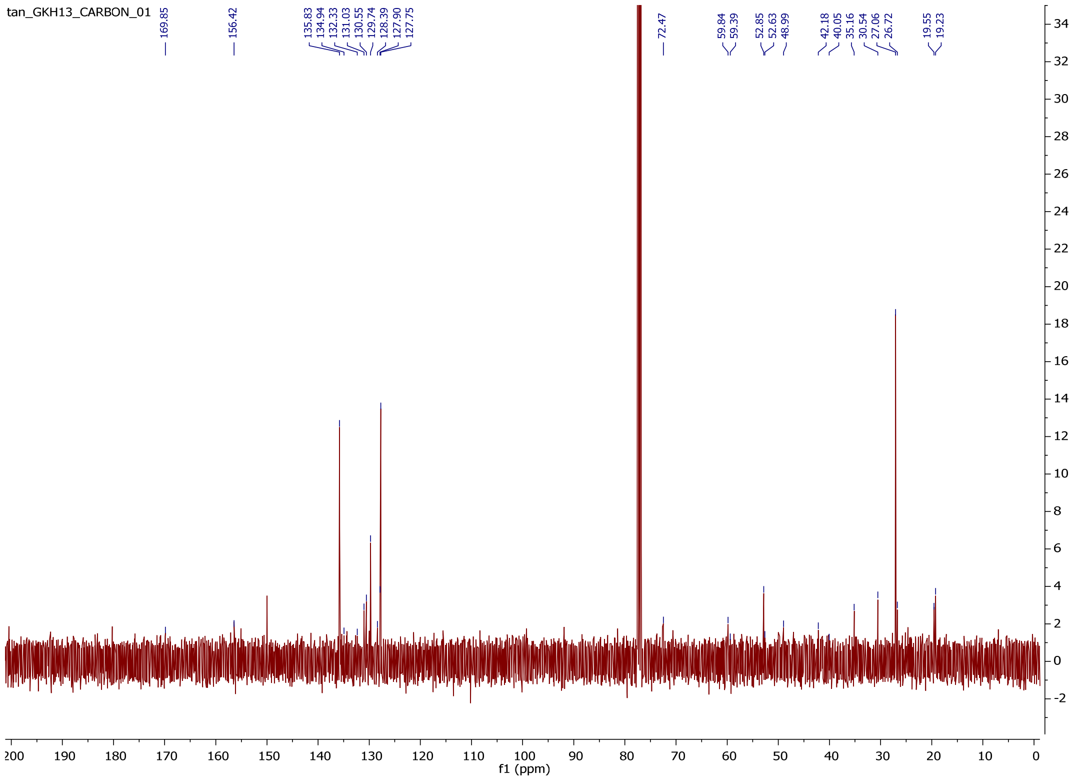

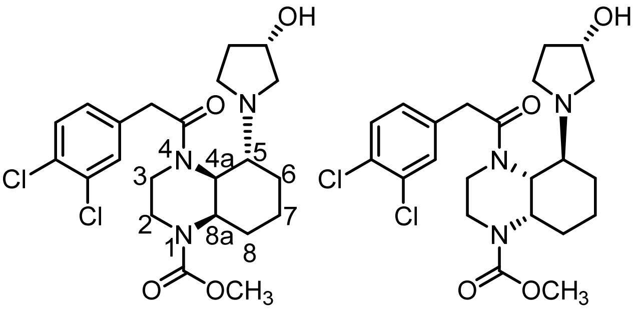
OH
O
N
4
N
4a
5
6
7
8
8a
1
N
2
3
Cl
Cl
O
OCH3
OH
O
N
N
Cl
Cl
N
O
OCH3

12c

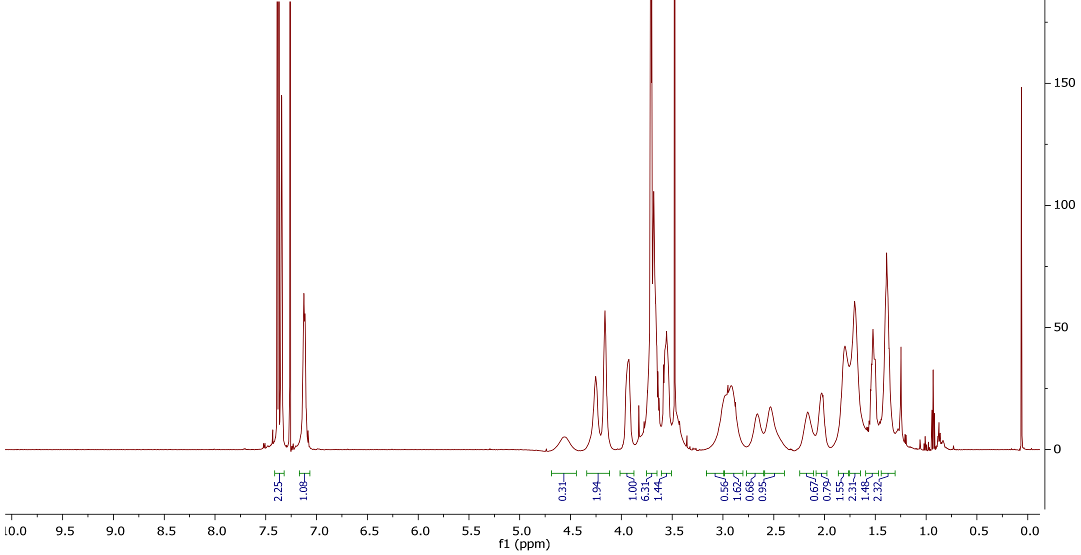
2.25
1.08
0.31
1.94
1.00
6.31
1.44
0.56
1.62
0.68
0.95
0.67
0.79
1.55
2.31
1.48
2.32
f1 (ppm)

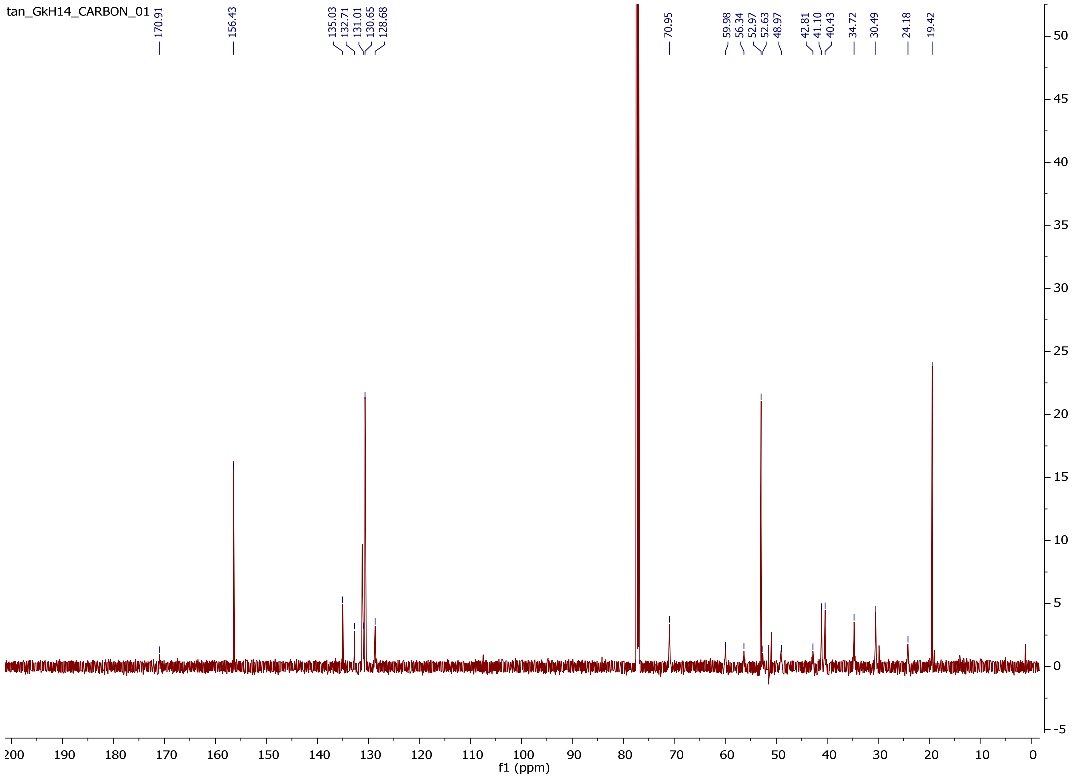
tan_GkH14_CARBON_01
170.91
156.43
135.03
132.71
131.01
130.65
128.68
70.95
59.98
56.34
52.97
52.63
48.97
42.81
41.10
40.43
34.72
30.49
24.18
19.42
f1 (ppm)

18

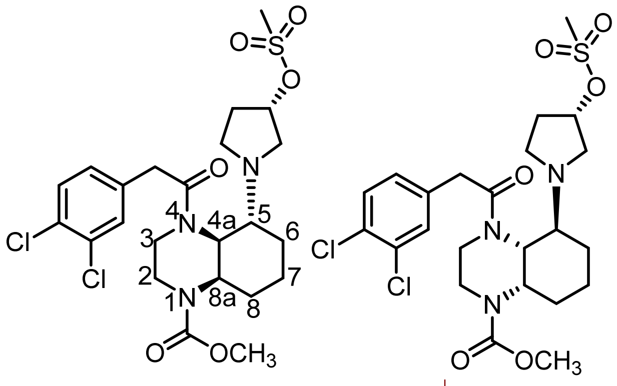
O=S=O
O
N
O
Cl
Cl
4
N
4a
5
6
3
2
7
1
N
8a
8
O
OCH$_3$

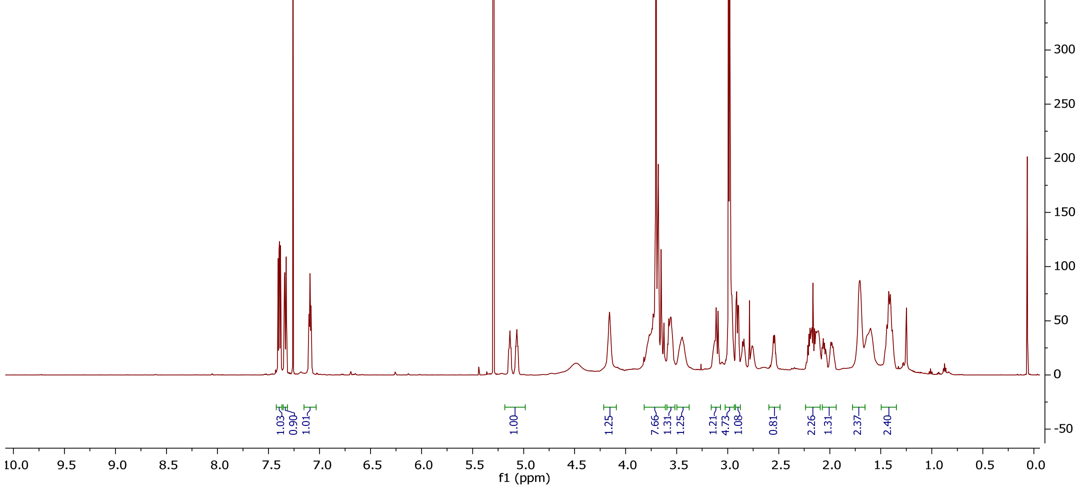
1.03
0.90
1.01
1.00
1.25
7.66
1.31
1.25
1.21
4.73
1.08
0.81
2.26
1.31
2.37
2.40
f1 (ppm)

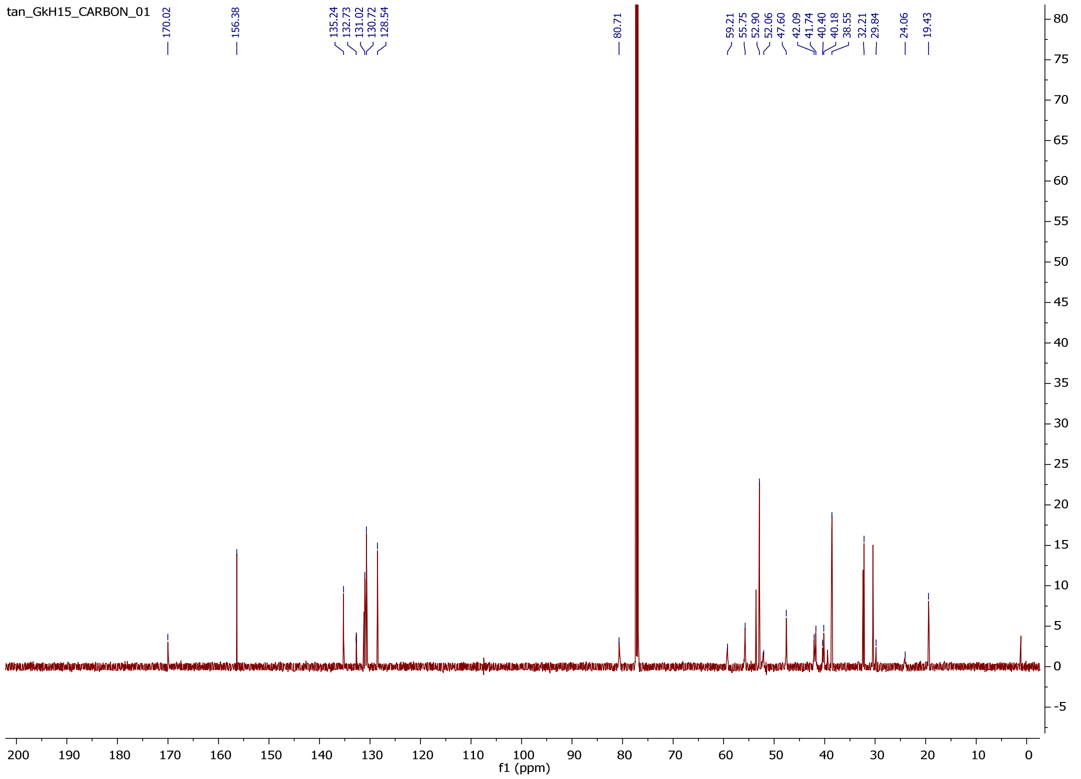
tan_GkH15_CARBON_01
170.02
156.38
135.24
132.73
131.02
130.72
128.54
80.71
59.21
55.75
52.90
52.06
47.60
42.09
41.74
40.40
40.18
38.55
32.21
29.84
24.06
19.43
f1 (ppm)

9. Purity data of test compounds

Purity by HPLC, method Pu

compound	purity
**12a**	96 %
**12b**	96 %
**12c**	99 %
**14b**	95 %
**14c**	96 %

10. HPLC chromatograms of test compounds

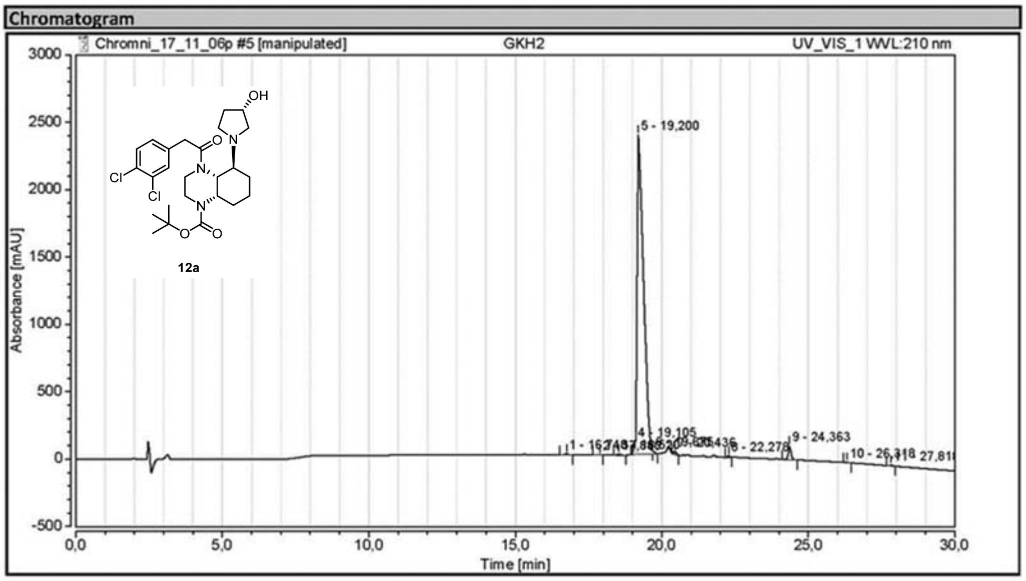

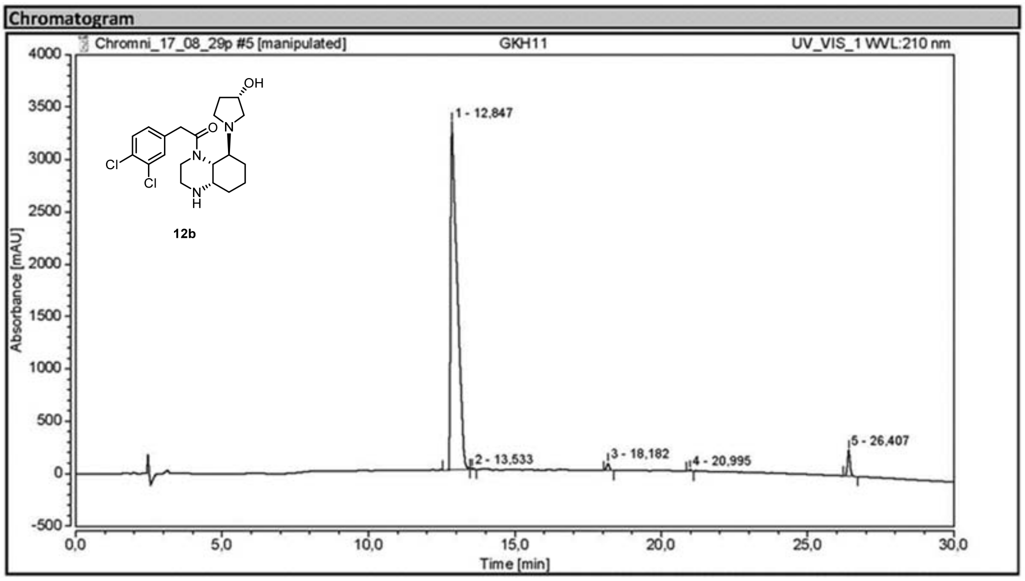

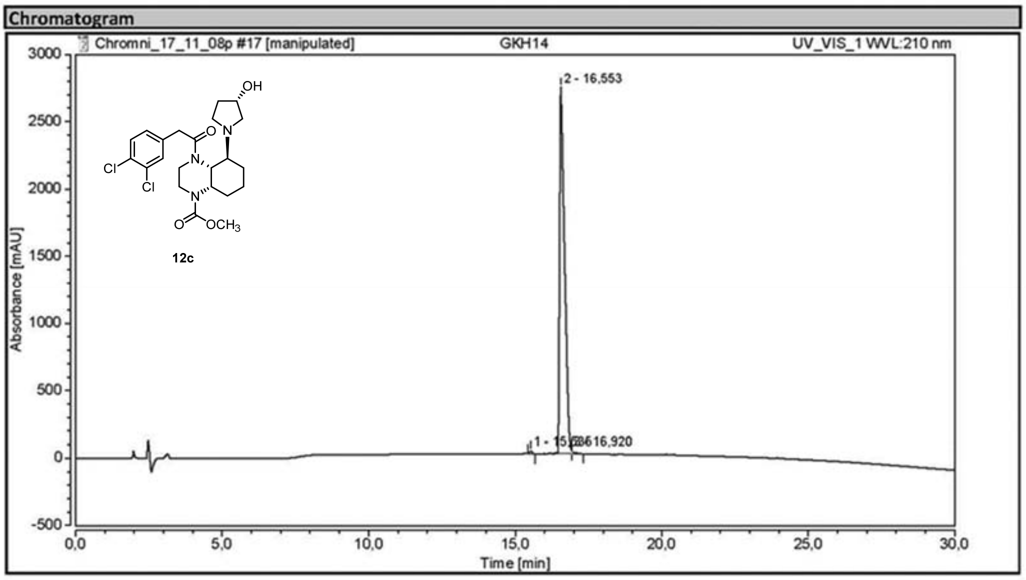
Chromatogram
Chromni_17_11_08p #17 [manipulated]
GKH14
UV_VIS_1 WVL:210 nm
2 - 16,553
OH
N
O
Cl
Cl
N
N
O
OCH3
12c
Absorbance [mAU]
Time [min]
3000
2500
2000
1500
1000
500
0
-500
0,0
5,0
10,0
15,0
20,0
25,0
30,0

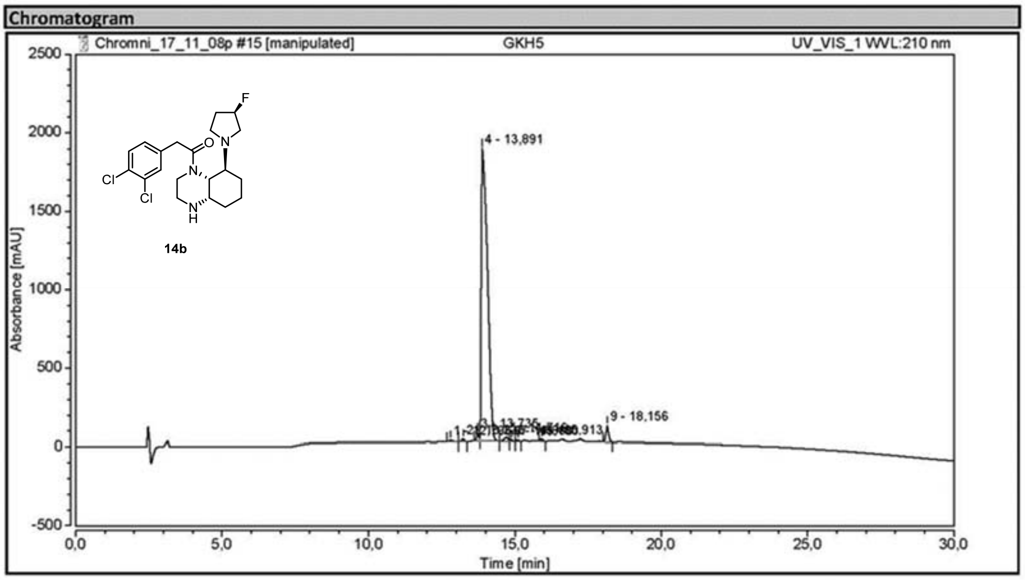
Chromatogram
Chromni_17_11_08p #15 [manipulated]
GKH5
UV_VIS_1 WVL:210 nm
4 - 13,891
9 - 18,156
F
N
O
Cl
Cl
N
NH
14b
Absorbance [mAU]
Time [min]
2500
2000
1500
1000
500
0
-500
0,0
5,0
10,0
15,0
20,0
25,0
30,0

Chromatogram

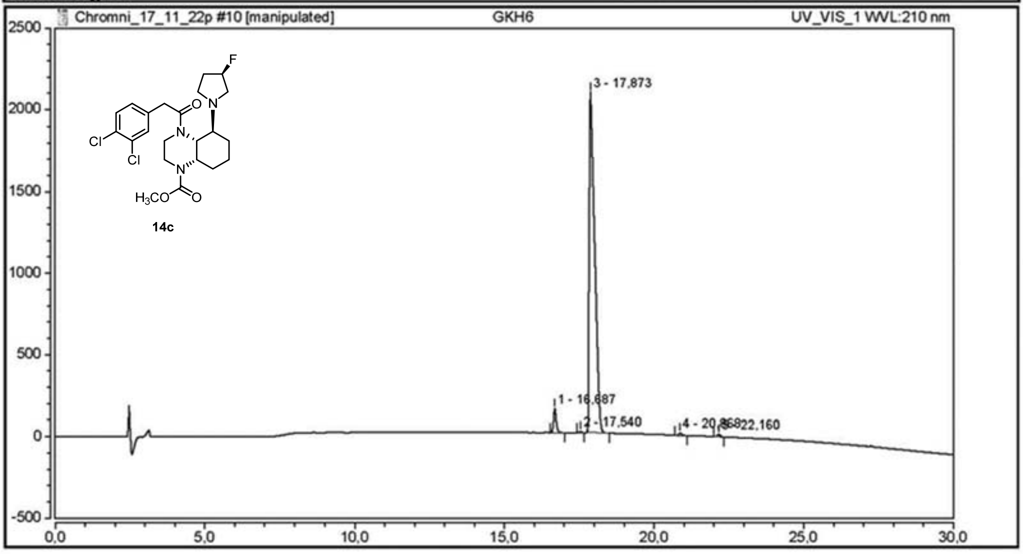

Supplement: Supplementary file 1 — Supplementary [file CMDC-15-1834-s001.pdf]
